# The Impact of Digitalization on European General Practice From the Perspective of General Practitioners: Systematic Review

**DOI:** 10.2196/83343

**Published:** 2026-08-18

**Authors:** Julia Magdalena Fuger, Lisa Niehoff, Erika Zelko

**Affiliations:** 1 Institute of General Practice, Faculty of Medicine, Johannes Kepler University, Altenbergerstrasse 69, Linz, 4020, Austria, 43 7322468 ext 8975

**Keywords:** digitalization, telehealth, general practice, primary care, eHealth, AI, systematic review

## Abstract

**Background:**

Digital health technologies, including telemedicine, electronic medical records, digital health tools (DHTs), and AI, are transforming European primary care, but evidence on how general practitioners (GPs) experience these tools remains fragmented.

**Objective:**

We aimed to synthesize European GPs’ perspectives on the adoption and implementation of digital health technologies, focusing on perceived usefulness, ease of use, implementation barriers, workload, and doctor-patient relationships.

**Methods:**

We conducted a systematic review following PRISMA (Preferred Reporting Items for Systematic Reviews and Meta-Analyses) 2020 and Synthesis Without Meta-Analysis guidance. Eight databases, PubMed, Scopus, SpringerLink, Wiley Online Library, Cochrane Library, Web of Science, Google Scholar, and Ovid, along with Elicit AI, were searched for primary studies on GPs’ perspectives in European primary care, published in English or German between January 2014 and December 2024; a search update extended coverage to October 2025. Eligible qualitative, quantitative, and mixed methods studies reported GP-perceived adoption, implementation, or impact of digital tools. Risk of bias was assessed using the Mixed Methods Appraisal Tool and JBI (Joanna Briggs Institute) critical appraisal tools. Data were synthesized using a narrative synthesis framework by Popay et al and a combined technology acceptance model and normalization process theory (NPT) effect-direction approach.

**Results:**

Of 1580 references, 65 studies including 24,994 GPs across 14 European countries (61/65, 93.8% from Western and Northern Europe) met the inclusion criteria. Studies covered AI or clinical decision support (n=7), DHTs (n=36), electronic medical records (n=4), and telemedicine (n=18). Perceived usefulness was high (56/65, 86.2% positive), whereas perceived ease of use was universally poor (0/65, 0% positive; 43/65, 66.2% negative), while actual use remained near universal (63/65, 96.9%). NPT constructs showed partial normalization: coherence 50.8% (33/65) positive; cognitive participation 4.6% (3/65) positive (22/65, 33.8%, negative); collective action 100% (65/65) partial; reflexive monitoring 66.2% (43/65) positive. Cross-cutting barriers included poor interoperability, fragmented platforms, limited training, misaligned incentives, and unreimbursed digital workload. GPs reported improved access for some patient groups but with concerns about depersonalized care and digital exclusion among older, low-literacy, rural, and socioeconomically disadvantaged patients.

**Conclusions:**

European general practice is extensively digitized but only partially normalized. GPs use digital tools because they must, not because they find them easy or workflow-enhancing. This review is, to our knowledge, the first to systematically synthesize European GPs’ experiences of digitalization across multiple technologies using a combined technology acceptance model-NPT effect-direction approach. It identifies a quantifiable “implementation paradox” in which high perceived usefulness and near-universal use coexist with poor usability and only partial workflow integration. These findings highlight the need for interoperable, user-centered systems that replace rather than add tasks, reimburse digital workload, provide structured digital-competency training, and set governance frameworks that safeguard equity and the quality of remote care in everyday general practice.

## Introduction

### Background

The digital transformation of primary care in Europe has accelerated in recent years, driven by policy imperatives, technological advancements, and the necessity for remote care during the COVID-19 pandemic [1,2]. Digitalization is reshaping general practice, with general practitioners (GPs) increasingly using electronic health records (EHRs) or electronic medical records (EMRs), telemedicine, and AI-based tools, such as clinical decision support systems (CDSS). While these technologies promise greater efficiency and access, their effects on everyday practice and professional roles vary across contexts [1,3,4]. Several reviews have examined digital health in primary care, including scoping and systematic reviews of digital health interventions and integrated care models in European primary care, as well as global reviews of telehealth, e-consultations, patient portals, and electronic CDSS tools.

Digital health technologies present both promise and paradox in general practice, with studies reporting improved patient access to care through e-consultations [5-7] and AI-driven triage tools [4,8], but their implementation is often problematic. For instance, while electronic CDSS can reduce diagnostic errors [4,9], they may simultaneously increase administrative workloads due to fragmented interfaces and alert fatigue [2,3,9,10]. Similarly, telemedicine has expanded access to underserved populations [6,11], yet GPs report disruptions to workflow continuity [12] and concerns about depersonalized care [5]. Though empowering for patients, symptom checkers and patient-operated triage tools risk misdiagnosis and generate additional consultation requests, thus amplifying GP workloads [8,13].

Integrating digital tools into primary care workflows has introduced significant challenges to workflows and the workforce. Pre–COVID-19 studies highlight that eHealth tools, such as EHRs, often failed to reduce administrative burdens, instead creating “double documentation” tasks [2]. Postpandemic, complex integrated care models supported by digital interventions have further strained GPs, requiring new competencies in data management and patient communication [1,14,15]. Machine learning applications that automate administrative tasks, such as coding and billing, show promise [16-18]. Still, their adoption is hindered by interoperability issues and a lack of trust in algorithmic outputs [4,18,19].

Regional disparities and competency gaps continue to shape the uneven impact of digitalization across Europe, reflecting differences in infrastructure, funding, and training [7]. Northern and Western European countries report higher adoption rates of EHRs and telemedicine, whereas Eastern European systems lag due to resource constraints [1,11]. Even in technologically advanced settings, GPs often lack the digital literacy needed to leverage AI tools effectively [16], exacerbating inequities in care quality [4,7,14]. Furthermore, ethical concerns about data privacy and algorithmic bias persist, particularly in AI-driven diagnostics, where transparency remains limited [4,9,13,17,18].

Despite growing research on patient outcomes and system efficiency, important gaps remain in the literature, particularly regarding GPs’ subjective experiences [3,20]. Several prior reviews have examined digital health in primary care, but largely from perspectives other than European GPs’ subjective experiences. Mold et al (2019) [5] synthesized evidence on patient-provider e-consultations in primary care, focusing on usage, access, and service outcomes rather than GP attitudes. Jimenez et al (2020) [14] mapped digital health competencies for primary care professionals, identifying skills and training needs but not providing an in-depth account of the day-to-day experiential consequences of digitalization. Fletcher et al (2023) [3] scoped the workload and workflow implications of electronic CDSS across high-income primary care systems, highlighting perceived time pressure, alert fatigue, and workflow disruption, but did not isolate European GPs’ wider views on digital transformation. Susanto et al (2023) [17] reviewed machine-learning-based CDSS and mainly reported effects on decision-making, care processes, and patient outcomes, while Radionova et al (2023) [13] and Gottliebsen and Petersson (2020) focused on symptom checkers and triage tools, emphasizing safety, demand, and mixed professional views rather than GPs’ broader subjective experience [8]. Sørensen et al (2023) [16] described machine learning applications and administrative automation in general practice, with attention to task support and efficiency [18], whereas Badr et al (2024) [21] examined digital health and inequalities at the population level, emphasizing structural and equity dimensions over frontline GP experience.

Other reviews have targeted specific domains such as telemedicine use barriers (Tabaeeian et al, 2024) [11], access systems in general practice (Eccles et al, 2024) [20], integrated digital care models in Europe (Mezzalira et al, 2024) [1], the impact of eHealth on general practice workload (Keuper et al, 2024) [2], and AI-based or conventional CDSS in primary care (Gomez-Cabello et al, 2024 [4]; Meunier et al, 2023 [9]; Susanto et al, 2023 [17]), typically prioritizing effectiveness, usage, or mixed professional samples rather than European GPs as a distinct group. Finally, Tun et al (2025) [19] and related work on trust in AI-CDSS, as well as Ambrosi et al (2025) [22] on digital interventions for chronic conditions, address acceptability and outcomes but not systematically the cumulative, everyday implications of digitalization for European GPs’ workload, workflow, autonomy, and doctor-patient relationships. As far as we are aware, no existing review has yet provided a focused synthesis of European GPs’ subjective experiences across digitalization. This review offers a dedicated synthesis of European GPs’ experiences across a broad range of digital tools (EHRs, telemedicine, digital health applications [DHAs] such as health portals, triage, and symptom-checker tools, and AI-based systems).

Therefore, the objective of this systematic review is to synthesize European GPs’ subjective experiences of digital health technologies between 2014 and 2024, using a combined effect-direction approach to characterize adoption and normalization processes and the “implementation paradox” in everyday general practice.

### Study Aims and Objectives

This systematic review explores GPs’ perspectives on digital health integration, focusing on the adoption trends of telemedicine, AI decision support, patient portals, and digital therapeutics; GPs’ experiences with benefits, workflow changes, and patient interactions; impacts on workload, efficiency, and ethical concerns (eg, data privacy and equity); barriers to implementation (technical, organizational, and patient-related); and policy recommendations for equitable, sustainable adoption. By synthesizing these insights, this review aims to support evidence-based policy and practice decisions for health care providers, policymakers, and digital health innovators.

## Methods

### Study Design

This systematic literature review was conducted following the PRISMA (Preferred Reporting Items for Systematic Reviews and Meta-Analyses) 2020 guidelines. All PRISMA checklists and the Synthesis Without Meta-Analysis reporting checklist [23] are available in Checklists 1 and 2 [23,24]. The review aimed to synthesize studies that explore GPs’ perspectives on digitalization in European primary care settings. The review protocol was registered retrospectively with the OSF (Open Science Framework; KE25C) on May 31, 2025 [25], after the initial search, but before data extraction, data synthesis, and analysis.

### Eligibility Criteria

We screened for papers that met the following criteria:

- Regarding population, concerning GPs or family physicians in European (European Union member states, United Kingdom, Norway, and Switzerland) primary care settings.
- Regarding phenomenon of interest, concerning digital health technologies (eg, EHRs, telemedicine, DHAs, AI, e-prescribing, and CDSS).
- Regarding language, concerning studies published in English or German. Language restrictions were applied based on the review team’s linguistic capabilities and the predominant languages of academic publications in this field.
- Regarding study design, being published, peer-reviewed, primary research papers using qualitative, quantitative, or mixed methods.
- Regarding outcomes, concerning GP-reported attitudes, experiences, or behaviors relating to the adoption, implementation, or impact of digital tools in general practice.
- Regarding time frame, concerning studies published between January 1, 2014, and December 31, 2024. The time frame captures recent digital health advancements and the accelerating pace of digitalization in primary care.

### Search Strategy

A comprehensive search was conducted across 8 electronic databases: PubMed, Scopus, SpringerLink, Wiley Online Library, Cochrane Library, Web of Science, Google Scholar, and Ovid. In addition, we used Elicit [26,27], a browser-based AI-powered literature review assistant, to identify additional gray literature records and to export structured bibliographic and study-level data under full human supervision. During the search period (November 2024 to January 2025), we accessed Elicit [26] via a Pro subscription through a standard web browser. The interface did not expose a model identifier or software version, so we describe the tool by provider, URL, subscription tier, and time window of use. No additional sources, such as reference lists of included studies, manual journal browsing, or direct contact with authors or organizations, were used to identify studies beyond the specified databases and Elicit [26]. We did not search study registries, set up citation alerts, or perform backward or forward citation chasing beyond what is reported in Multimedia Appendix 1. The primary search was completed on January 5, 2025. A combination of free-text terms and Boolean operators was used, including: ("digital" OR "digitalization" OR "eHealth" OR "telemedicine" OR "artificial intelligence") AND ("primary care" OR "general practice" OR "general practitioners"). The search strategy was developed iteratively by JMF and was not prospectively peer-reviewed using a standardized checklist; instead, we conducted a retrospective PRESS (Peer Review of Electronic Search Strategies) 2015 assessment after the searches were completed [28]. We evaluated the full strategy against the core PRESS domains, including the translation of the review question into search concepts, the choice of subject headings and text words across databases, the use of Boolean and proximity operators, the application of limits and filters (time period, language, and study design), and the overall structure and syntax of the search strings. This assessment did not identify any major errors or omissions in these domains; only minor wording refinements were suggested, which did not materially alter the sensitivity or specificity of the original strategy. The entire search strategy and a detailed PRESS assessment table are provided in Multimedia Appendix 1 [29-33] and Checklist 3. Our search strategy was structured around the PICO (Population, Intervention, Comparator, and Outcome) elements of the review question (European GPs in primary care; digital health technologies; GP-reported experiences and impacts) and implemented using extensive synonyms and Boolean operators. Database-specific field tags (for example, PubMed [Title]/[pdat], Scopus TITLE-ABS-KEY, Cochrane ti,ab,kw, Web of Science topic fields) and subject headings were applied where available, but heterogeneous interfaces and uneven indexing for digital health meant we relied primarily on text-word searching and phrase searching rather than systematic use of MeSH/EMTREE and proximity operators. A search update was performed later on October 7, 2025, by rerunning the entire search using identical search strings and databases to identify studies published between January 1, 2025, and October 7, 2025. The updated search results are provided in Multimedia Appendix 2, but were not included in the present synthesis.

### Study Selection

The initial search yielded 1580 references. After automatic and manual deduplication in the Rayyan web application, 251 duplicate entries were removed, yielding 1329 unique papers. Title screening excluded 929 papers that did not meet the inclusion criteria. Abstracts of the remaining 400 papers were assessed, and 118 studies were selected for full-text review. Following full-text evaluation, 65 studies met all eligibility criteria and were included in the final synthesis. A total of 53 full-text papers were excluded from the review. A detailed list of these excluded studies, along with the specific reasons for their exclusion, is provided in Multimedia Appendix 3. One primary reviewer (JMF) conducted the screening process. A second reviewer (LN) independently screened 20% (266/1329) of the total records during title, abstract (80/400), and full-text screening (24/118). Interrater agreement was excellent across all screening phases: 95.86% (255/266) at title screening (Cohen κ=0.84), 95% (76/80) at abstract screening (κ=0.87), and 95.83% (23/24) at full-text screening (κ=0.92). As all disagreement rates were below the prespecified 5% threshold, no additional screening was required. Discrepancies were resolved through discussion and consensus during team meetings. The final decision on inclusion or exclusion was made by a third reviewer (EZ). Data were stored in Zotero, and screeners’ decisions were noted in the Rayyan web application.

### Data Extraction

Elicit AI [26] and JMF performed data extraction. The preset extraction sets from Elicit AI [26] were used (eg, study metadata, study design, methodologies, study participants’ sample sizes, digital health intervention type, GPs’ perceptions or outcomes, quantitative results [if available], etc) to extract study data. In the first step, training and calibration were conducted using 5 studies, where extractions were performed by Elicit AI [26] and independently by the first author. A 100% overlap of extracted information was found. JMF cross-checked all finalized data extracted by Elicit AI [26]; in case of discrepancies, the reviewer used the manually extracted data. No authors were contacted for missing or unclear data.

Extracted data included the following:

- Authors, publication year, and country of study.
- Study design and methodology.
- Participant characteristics and sample size.
- Type of digital health interventions.
- Outcome domains:
  - Regarding reported perceptions of digital health technologies, GPs’ attitudes, beliefs, and general acceptance or skepticism toward various digital tools (eg, AI, telemedicine, and EHRs).
  - Regarding impact on clinical practice, effects on diagnostic processes, treatment decisions, and overall quality of care.
  - Regarding impact on workflow efficiency, changes in administrative burden, time management, and operational processes within the practice.
  - Regarding impact on patient relationships, changes in doctor-patient communication, trust, and continuity of care.
  - Regarding perceived benefits, advantages reported by GPs (eg, improved access, enhanced information, and decision support).
  - Regarding perceived challenges or barriers, obstacles encountered (eg, increased workload, data privacy, technical issues, interoperability, training gaps, and financial concerns).
  - Regarding policy implications and recommendations, suggestions for future policy, practice, and research.

For each included study, all reported findings that were compatible with the defined outcome domains were extracted. No selective collection of results was performed within eligible domains. The complete data-extraction table for all 65 included studies, covering every extracted variable, is provided in full in Multimedia Appendix 4 [29-93], and a condensed evidence summary (author, country, and key findings for each study) is provided in Multimedia Appendix 5 [29-93]; owing to their length, both are presented as Multimedia Appendices rather than in the main text, in line with JMIR house-style guidance for oversized tables.

### Quality Assessment and Data Synthesis

Included studies were subject to critical appraisal using the Mixed Methods Appraisal Tool (MMAT) 2018 version [94]. No formal quality threshold was applied at the inclusion stage; all 65 studies contributed to the narrative synthesis irrespective of MMAT score. MMAT ratings were used descriptively to characterize overall study quality and to avoid overinterpreting isolated findings from lower-scoring studies. A total of 4 studies scored 60%, and the remaining 61 studies scored ≥80%; no study scored below 60%. We did not apply a formal GRADE (Grading of Recommendations Assessment, Development and Evaluation) approach. The papers’ quality was assessed manually by JMF and checked for disagreements by the other authors. The MMAT score table and a vote-counting analysis for the four 60% MMAT studies are reported in Multimedia Appendix 6 [29-93]. Design-specific JBI (Joanna Briggs Institute) critical appraisal tools were used to assess the risk of bias for all included studies. Qualitative studies were appraised using the JBI Qualitative Research checklist [95]; quantitative descriptive or cross-sectional studies, the JBI Analytical Cross-Sectional checklist [96]; and mixed methods studies, their qualitative and quantitative components were appraised separately using the relevant JBI tools. Study-level risk-of-bias results are reported in the Results, and a color-coded version of the appraisal is provided in Multimedia Appendix 6. Limited sensitivity analyses for respondent overlap and study quality (MMAT) are reported in Multimedia Appendix 7 as side-by-side comparison tables showing percentage distributions with and without the potentially overlapping or lower-MMAT studies. Given the marked heterogeneity of this literature, we did not undertake a formal meta-analysis. Instead, a narrative synthesis approach, following the framework of Popay et al (2006) [24], was used. Within this framework, we applied two complementary theoretical lenses. The technology acceptance model (TAM) [97] was used to analyze individual GP adoption decisions, focusing on perceived usefulness and ease of use. The normalization process theory (NPT) [98] was used to examine implementation processes, with coding for coherence, cognitive participation, collective action, and reflexive monitoring. Studies were grouped into four mutually exclusive categories (AI, digital health tools [DHTs], EHRs, and telemedicine). No changes were made to the planned groupings after protocol registration. Studies covering multiple technologies without a specific focus on AI, EMRs, or telemedicine were classified as DHTs. Each study was assigned to exactly one category, with subcategories providing additional granularity within the main classification scheme. The topic coding, along with all extracted data, is shown in Multimedia Appendix 4. Descriptive statistics, such as frequencies and percentages, were used to characterize the included studies (eg, methodological approaches, temporal trends, and geographic distribution) and to summarize quantitative findings from individual studies where available. We also created an effect-direction table to visualize heterogeneous findings from TAM and NPT analyses. We transformed study-level results into direction-of-effect categories (strong positive, positive, mixed, negative, and strong negative) using prespecified percentage thresholds, and then summarized the proportions of studies in each category. The included studies reported different but partly overlapping constructs across TAM and NPT domains, combined qualitative, quantitative, and mixed methods designs, and operationalized key outcomes using noncomparable, study-specific measurement scales, with only very small numbers of directly comparable studies per technology-country combination, often from different time periods. Under these conditions, a quantitative meta-analysis would risk producing a misleading “fruit-salad” summary [99]. No further statistical transformations of effect estimates were undertaken. JMF conducted the data synthesis, which the other authors further developed.

### Deviations From the Protocol

The review departed from the registered protocol (OSF; KE25C) in the following respects. The protocol was registered retrospectively, as noted under the Study Design section. Screening was conducted primarily by one reviewer (JMF), with a second reviewer (LN) independently screening a 20% sample at each stage and a third reviewer (EZ) making the final inclusion decisions, rather than full dual screening. In addition to the planned MMAT appraisal, design-specific JBI tools were added for a formal risk-of-bias assessment. Beyond the planned narrative and thematic synthesis, a combined TAM-NPT effect-direction approach was applied, and the prespecified rule of treating a theme as robust when it appeared in ≥30% of studies and across at least three countries was operationalized through the effect-direction thresholds reported above. Post hoc sensitivity analyses (respondent overlap; exclusion of studies scoring 60% on the MMAT) were added in response to peer review (Multimedia Appendix 7 [35,38,46,48,64,65,68]). A search update to October 2025 was conducted but not synthesized (Multimedia Appendix 2). Finally, one pan-European survey was retained in which 1 of the 31 countries surveyed was non-European (1/31, 3.2%), as the aggregated data could not be disaggregated. None of these deviations changed the review question or the eligibility criteria or altered the overall conclusions.

## Results

### Overview

The studies were clustered into topics; a complete list of all extracted parameters, including key findings for each study, is available in Multimedia Appendices 4 and 5. DHTs comprised 55.4% (36/65) [29-31,34-66] of the studies, with general information and communication technology tools prominent (18/36) [29,31,34-38,40-42,44,47,48,50,53,59,64,66], and DHAs were explicitly examined in 6 of 36 studies [54-56,61,63,65]. Telemedicine accounted for 27.7% (18/65) [32,67-83], including studies on COVID-19 impacts (4/18) [72,73,78,81], video consultations (VCs) (4/18) [68,69,76,80], and email consultations (4/18) [67,71,79,83]. AI applications comprised 10.8% (7/65) [33,84-89], and EMR or EHR studies represented 6.2% (4/65) [90-93].

### Screening Process

**Figure 1.**
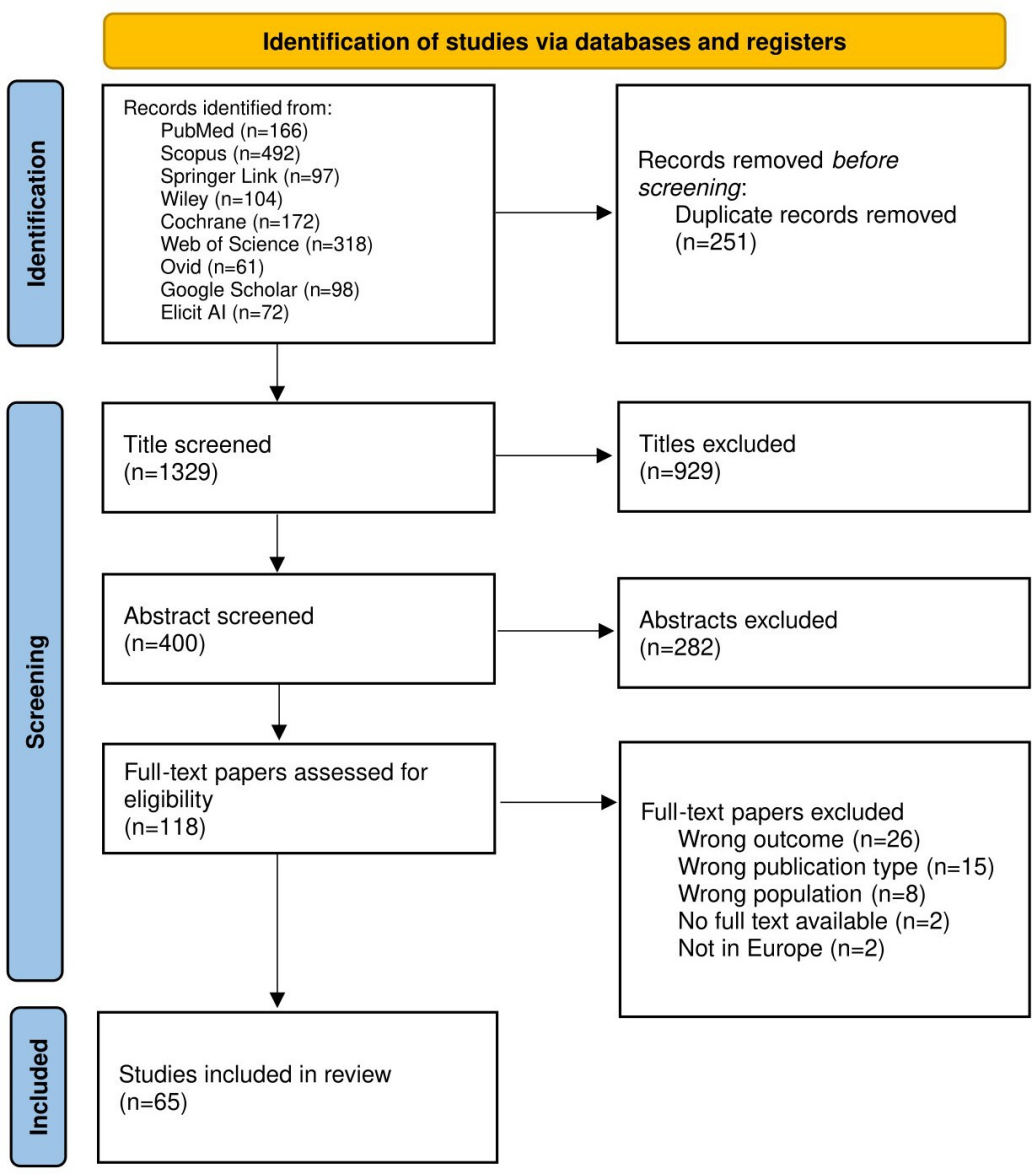
PRISMA (Preferred Reporting Items for Systematic Reviews and Meta-Analyses) flowchart [100].

The screening process is depicted in Figure 1.

### Study Characteristics

#### Sample Sizes and Participant Characteristics

The 65 studies included a total of 24,994 GPs. Sample sizes varied considerably, with a few large-scale surveys accounting for most participants. One pan-European study across 31 European countries included 9196 GPs [38], another German survey had 5868 GP respondents [65], and 4 more studies enrolled more than 1000 participants [39,46,72,89]. Most other quantitative studies surveyed several hundred GPs each, while qualitative studies typically interviewed 10‐30 GPs per study.

#### Study Design and Methods

The 65 papers comprised primarily qualitative research (n=32, 49.2%) [30-32,37,42,44,45,49,52,54,55,57-61,63,69,71,73,74,76,77,79-84,87,92,93], observational quantitative descriptive studies with a cross-sectional approach (n=17, 26.2%) [29,38-40,46,48,53,56,64,68,72,75,85,86,88-90], and mixed methods design (n=16, 24.6%) [33-36,41,43,47,50,51,62,65-67,75,78,91], with a focus on exploratory and implementation research. Methodological approaches included Likert-scale questionnaires, qualitative interviews, and mixed-method triangulation. Although all studies relied on qualitative interviews, questionnaires, or surveys, 32 studies also used a quantitative approach [29,34-36,38-41,43,46,48,50,51,53,55,56,62,64-68,70,72,75,78,81,85,86,88,89]. Thematic coding was ubiquitous, with some studies applying theoretical frameworks such as the TAM or the COM-B (Capability, Opportunity, Motivation – Behavior) change model.

#### Temporal Distribution

Most studies (52/65, 80%) were conducted between 2020 and 2024. Table 1 shows the temporal distribution.

**Table 1.** Temporal distribution of the 65 included studies by publication year.

Year	Study count, n (%)
2014	1 (1.5)
2016	1 (1.5)
2017	2 (3.1)
2018	4 (6.2)
2019	5 (7.7)
2020	6 (9.2)
2021	7 (10.8)
2022	8 (12.3)
2023	13 (20)
2024	18 (27.7)

#### Geographic Distribution

**Figure 2.**
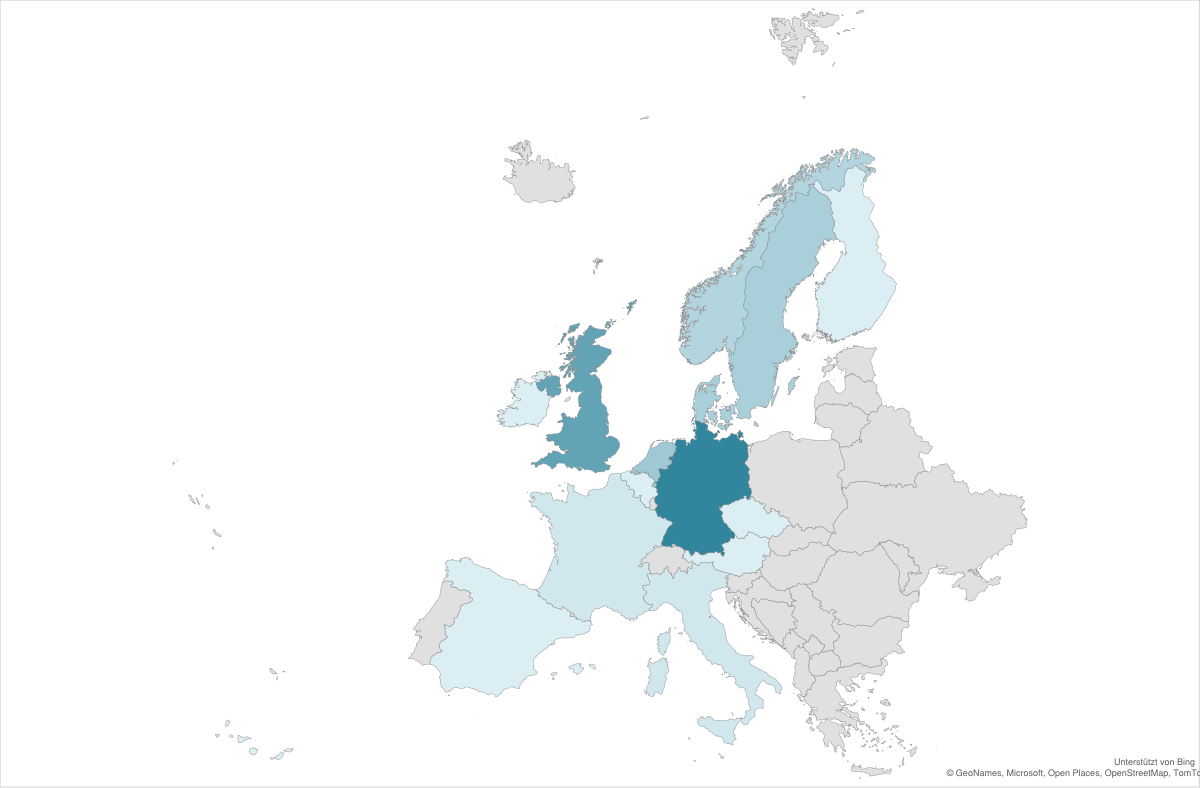
Geographic distribution of the 65 included studies across Europe. Darker colors indicate a greater number of studies (created using the Bing Maps integration in Microsoft Excel [101], which is published under limited license per the Microsoft Bing Maps Terms of Use [102]).

Studies were conducted across 14 European countries; one study from Spain was a multicountry study covering 31 European countries [38]. Geographic distribution was determined by the country where each study was conducted. For the single multicountry study (Torrent-Sellens et al, 2018 [38]), which reported only aggregated results, we attributed it to Spain (the lead institution’s country) to avoid overcounting. Table 2 and Figure 2 show the geographic distribution of studies.

**Table 2.** Geographic distribution of the included studies by country.

Country	Count, n (%)
Germany	18 (27.7)
United Kingdom	13 (20)
Netherlands	7 (10.8)
Denmark	6 (9.2)
Sweden	6 (9.2)
Norway	5 (7.7)
France	2 (3.1)
Italy	2 (3.1)
Austria	1 (1.5)
Belgium	1 (1.5)
Czech Republic	1 (1.5)
Finland	1 (1.5)
Ireland	1 (1.5)
Spain	1 (1.5)

### Risk of Bias Assessment

Appraised with the design-appropriate JBI tools described above, most of the 65 studies were at low to moderate risk of bias; only three—all short conference-proceeding papers, in which the publication format rather than the design limits methodological transparency—were rated high risk.

The most frequent limitations were incomplete reporting of researcher reflexivity in the qualitative and mixed methods studies and unclear handling of confounding and measurement validity in the cross-sectional studies. These did not invalidate any study; high-risk studies were interpreted with caution, were never the sole basis for a key conclusion, and their exclusion did not change the overall results (Multimedia Appendix 7). The full item-level appraisal for each study is shown in Tables 3-5.

**Table 3.** JBI^a^ risk-of-bias appraisal of the 31 qualitative studies. Items (JBI qualitative research checklist).

Author and year	Q1^b^	Q2^c^	Q3^d^	Q4^e^	Q5^f^	Q6^g^	Q7^h^	Q8^i^	Q9^j^	Q10^k^	Yes (%)	Risk
Hvidt et al, 2021 [71]	Y^l^	Y	Y	Y	Y	Y	U^m^	Y	Y	Y	90	LR^n^
Bondaronek et al, 2022 [49]	U	Y	Y	Y	Y	N^o^	N	Y	Y	Y	70	MR^p^
Buck et al, 2022 [84]	Y	Y	Y	Y	Y	N	N	Y	U	Y	70	MR
Butler et al, 2023 [52]	Y	Y	Y	Y	Y	Y	Y	Y	Y	Y	100	LR
Davidge et al, 2023 [92]	U	Y	Y	Y	Y	N	N	Y	Y	Y	70	MR
Entezarjou et al, 2020 [42]	Y	Y	Y	Y	Y	Y	Y	Y	Y	Y	100	LR
Fagerlund et al, 2019 [31]	U	Y	Y	Y	Y	N	N	Y	Y	Y	70	MR
Gabriels and Moerenhout, 2018 [37]	U	Y	Y	Y	Y	N	N	Y	U	Y	60	MR
Gavin et al, 2024 [57]	U	Y	Y	Y	Y	N	N	Y	Y	Y	70	MR
Malfilatre and Louvel, 2025^q^ [58]	U	Y	Y	Y	Y	U	N	Y	U	Y	60	MR
Glock et al, 2021 [44]	U	Y	Y	Y	Y	Y	Y	Y	Y	Y	90	LR
Grønning et al, 2024 [83]	Y	Y	Y	Y	Y	Y	Y	Y	Y	Y	100	LR
Jakobsen et al, 2021 [45]	U	Y	Y	Y	Y	N	N	Y	Y	Y	70	MR
Klausen and Hvidt, 2024 [79]	Y	Y	Y	Y	Y	Y	U	Y	Y	Y	90	LR
Kofod et al, 2024 [80]	Y	Y	Y	Y	Y	Y	Y	Y	Y	Y	100	LR
Kollmann et al, 2024 [81]	U	Y	Y	Y	Y	N	N	Y	Y	Y	70	MR
Lüchau et al, 2023 [76]	Y	Y	Y	Y	Y	Y	Y	Y	Y	Y	100	LR
Meurs et al, 2022 [73]	U	Y	Y	Y	Y	N	N	Y	Y	Y	70	MR
Morrissey et al, 2018 [30]	U	Y	Y	Y	Y	N	N	Y	U	Y	60	MR
Mueller et al, 2020 [69]	Y	Y	Y	Y	Y	N	N	Y	U	Y	70	MR
Nassehi and Ramvi, 2025^r^ [59]	U	Y	Y	Y	Y	N	N	Y	Y	Y	70	MR
Newbould et al, 2024 [60]	U	Y	Y	Y	Y	N	N	Y	U	Y	60	MR
Norberg et al, 2024 [32]	Y	Y	Y	Y	Y	Y	Y	Y	Y	Y	100	LR
Peeters et al, 2023 [77]	U	Y	Y	Y	Y	N	N	Y	Y	Y	70	MR
Posselt et al, 2024 [61]	U	Y	Y	Y	Y	N	N	Y	U	Y	60	MR
Schütze et al, 2023 [87]	U	Y	Y	Y	Y	N	N	Y	Y	Y	70	MR
Carlsson et al, 2023 [54]	U	Y	Y	Y	Y	N	N	Y	Y	Y	70	MR
Stamer et al, 2024 [82]	U	Y	Y	Y	Y	N	N	Y	Y	Y	70	MR
Turner et al, 2022 [74]	U	Y	Y	Y	Y	N	N	Y	Y	Y	70	MR
Turner et al, 2023 [93]	U	Y	Y	Y	Y	N	N	Y	Y	Y	70	MR
Wangler and Jansky, 2023 [55]	U	Y	Y	Y	Y	N	N	Y	U	Y	60	MR

a JBI: Joanna Briggs Institute.

b Q1: philosophy-methodology congruity.

c Q2: methodology-research question.

d Q3: methodology-data collection.

e Q4: methodology-data analysis.

f Q5: methodology-interpretation.

g Q6: researcher located culturally or theoretically.

h Q7: researcher influence addressed.

i Q8: participant voices represented.

j Q9: ethical approval or considerations.

k Q10: conclusions flow from the analysis.

l Y: yes.

m U: unclear.

n LR: low risk (≥80% yes).

o N: no.

p MR: moderate risk (60%‐79%).

q The study by Malfilatre and Louvel [58] was published and first available online on November 29, 2024, and was hence included in this systematic review.

r The study by Nassehi and Ramvi [59] was published and first available online on December 5, 2024, and was hence included in this systematic review.

**Table 4.** JBI^a^ risk-of-bias appraisal of the 16 analytical cross-sectional studies. Items (JBI analytical Cross-Sectional checklist).

Author and year	C1^b^	C2^c^	C3^d^	C4^e^	C5^f^	C6^g^	C7^h^	C8^i^	Yes (%)	Risk
Albrecht et al, 2017 [29]	Y^j^	N/A^k^	Y	Y	Y	Y	Y	Y	86	LR^l^
Blease et al, 2024 [89]	Y	N/A	Y	Y	Y	U^m^	Y	Y	71	MR^n^
Brecher et al, 2024 [56]	Y	N/A	Y	Y	Y	U	Y	Y	71	MR
Breedvelt et al, 2019 [39]	Y	N/A	Y	Y	Y	U	Y	Y	71	MR
Buhtz et al, 2019 [40]	Y	N/A	Y	Y	Y	U	Y	Y	71	MR
Cabitza et al, 2014 [90]	U	N/A	U	U	N^o^	N	U	Y	14	HR^p^
Johnsen et al, 2021 [72]	Y	N/A	Y	Y	Y	Y	Y	Y	86	LR
Keuper et al, 2021 [46]	Y	N/A	Y	Y	Y	U	Y	Y	71	MR
Kujansivu et al, 2023 [53]	Y	N/A	Y	Y	Y	U	Y	Y	71	MR
Mahlknecht et al, 2023 [86]	Y	N/A	Y	Y	Y	U	Y	Y	71	MR
Pikkemaat et al, 2021 [48]	Y	N/A	Y	Y	Y	Y	Y	Y	86	LR
Tabla et al, 2022 [85]	Y	N/A	Y	Y	U	U	Y	U	43	HR
Torrent-Sellens et al, 2018 [38]	Y	N/A	Y	Y	Y	Y	Y	Y	100	LR
Waschkau et al, 2020 [70]	Y	N/A	Y	Y	Y	U	Y	Y	71	MR
Waschkau et al, 2022 [75]	Y	N/A	Y	Y	Y	U	Y	Y	71	MR
Wewetzer et al, 2023 [88]	Y	N/A	Y	Y	Y	U	Y	Y	71	MR

a JBI: Joanna Briggs Institute.

b C1: inclusion criteria defined.

c C2: objective/standard measurement of the condition (not applicable—attitude/perception surveys).

d C3: valid, reliable exposure measurement.

e C4: valid, reliable outcome measurement.

f C5: confounders identified.

g C6: strategies to address confounders.

h C7: appropriate statistical analysis.

i C8: subjects and setting described.

j Y: yes.

k N/A: not applicable.

l LR: low risk (≥80% yes).

m U: unclear.

n MR: moderate risk (60%‐79%).

o N: no.

p HR: high risk (<60%).

**Table 5.** JBI^a^ risk-of-bias appraisal of the 18 mixed methods studies, appraised with both checklists. Yes (%) is computed across all applicable items from both checklists^b^.

Author and year	C1	C2	C3	C4	C5	C6	C7	C8	Q1	Q2	Q3	Q4	Q5	Q6	Q7	Q8	Q9	Q10	Yes (%)	Risk
Blease et al, 2019 [33]	Y	N/A	Y	Y	Y	U	Y	Y	U	Y	Y	Y	Y	N	Y	Y	Y	Y	82	LR
Blease et al, 2023 [91]	Y	N/A	Y	Y	Y	U	Y	Y	U	Y	Y	Y	Y	N	Y	Y	Y	Y	82	LR
Cowie et al, 2018 [36]	Y	N/A	Y	Y	U	U	Y	Y	U	Y	Y	Y	Y	N	N	Y	Y	Y	71	MR
Hammerton et al, 2022 [50]	Y	N/A	Y	Y	U	U	Y	Y	U	Y	Y	Y	Y	N	N	Y	Y	Y	71	MR
Johansson et al, 2020 [67]	Y	N/A	Y	Y	U	U	Y	Y	U	Y	Y	Y	Y	N	N	Y	U	Y	65	MR
Klocek et al, 2019 [41]	Y	N/A	Y	Y	Y	Y	Y	Y	U	Y	Y	Y	Y	N	N	Y	Y	Y	82	LR
Knop et al, 2021 [47]	Y	N/A	Y	Y	Y	U	Y	Y	U	Y	Y	Y	Y	N	N	Y	Y	Y	76	MR
Koch and Guhres, 2020 [68]	Y	N/A	Y	U	U	U	Y	Y	U	Y	Y	Y	Y	N	N	Y	U	Y	59	MR
Kriegel et al, 2017 [35]	U	N/A	Y	U	U	N	Y	Y	U	Y	U	U	U	N	N	Y	U	Y	35	HR
Peeters et al, 2016 [34]	Y	N/A	Y	Y	U	U	Y	Y	U	Y	Y	Y	Y	N	N	Y	U	Y	65	MR
Poss-Doering et al, 2020 [43]	Y	N/A	Y	Y	Y	Y	Y	Y	Y	Y	Y	Y	Y	Y	Y	Y	Y	Y	100	LR
Potthoff et al, 2024 [62]	Y	N/A	Y	Y	Y	Y	Y	Y	Y	Y	Y	Y	Y	N	N	Y	Y	Y	88	LR
Preiser et al, 2024 [63]	U	N/A	Y	Y	U	U	Y	Y	Y	Y	Y	Y	Y	Y	Y	Y	Y	Y	82	LR
Schendzielorz et al, 2023 [64]	Y	N/A	Y	Y	U	U	Y	Y	U	Y	Y	Y	Y	N	N	Y	Y	Y	71	MR
Tensen et al, 2023 [78]	Y	N/A	Y	Y	U	U	Y	Y	U	Y	Y	Y	Y	N	N	Y	Y	Y	71	MR
van de Vijver et al, 2022 [51]	Y	N/A	Y	Y	Y	Y	Y	Y	U	Y	Y	Y	Y	N	N	Y	Y	Y	82	LR
Wangler and Jansky, 2024 [65]	Y	N/A	Y	Y	Y	U	Y	Y	U	Y	Y	Y	Y	N	N	Y	U	Y	71	MR
Weik et al, 2024 [66]	Y	N/A	Y	Y	Y	Y	Y	Y	Y	Y	Y	Y	Y	Y	Y	Y	Y	Y	100	LR

a JBI: Joanna Briggs Institute.

b C1-C8 are the cross-sectional items and Q1-Q10 the qualitative items, as defined in Tables 3 and 4 (C2 not applicable for all). Y: yes; N: no; U: unclear; N/A: not applicable; LR, MR, and HR as in Table 3.

### Summary of Main Findings

#### Adoption Trends of Digital Health Technologies

Digital health adoption in European general practice was heterogeneous across technology types, regions, and temporal contexts. EMR or EHR adoption reached 98% (n=800 GPs) in an Italian study [90]. Beyond that, 3 UK studies explored the implementation and impact of patients’ online access to their primary care EHRs [91-93]. Despite some challenges, the move toward online record access continues, with National Health Service (NHS) England having planned to enable full prospective access for all adult patients in 2023, as reported by Davidge et al (2023) [92,103]. NHS England followed up on this policy initiative in 2023, and since late 2023, patients have been able to access their GP health record via the NHS app [104]. Yet more advanced technologies show variable uptake: only 20% of UK GPs (n=1006) reported using generative AI, primarily for documentation and diagnostic support [89]. According to UK and French studies, GPs expect AI to assist with urgent diagnoses, improve efficiency, and integrate seamlessly with existing systems [33,85], with emphasis on transparency, probabilistic differential diagnosis displays, and atypical disease presentations [87]. Digital self-tracking (eg, for hypertension in Ireland), online consultation tools in Belgium and the United Kingdom, and e-mental health interventions in the United Kingdom are increasingly recognized as beneficial for patient empowerment and self-management [30,36,37,39]. However, success depends on practical implementation and “champions” within practices to overcome adoption barriers [36,37]. Digital platforms for physical activity advice and long-term condition (LTC) management showed promise, with active users and GPs reporting significant benefits in 3 UK studies and 1 Dutch study [49,51,57,60]. However, uptake remains uneven: in 1 German study, only 59.4% of GPs (n=97) prescribed DHAs despite positive attitudes [56], and 1 Norwegian study rated a digital alcohol intervention’s use in routine practice as poor [62]. This was reflected in 2 other German studies on DHAs [55,65]. Further, 1 study revealed that 68% (n=96 GPs) viewed DHAs positively, considering them reliable and safe tools to improve doctor-patient relationships [55]. For symptom-checker apps, most GPs in 1 German study assessing the Ada app were open to health care digitization. Still, they were unfamiliar with these tools, viewing them as part of patients’ initial information-seeking processes [63]. Additionally, the CHANGE-3 study in Germany on antibiotic prescribing, which offered tablets, e-learning platforms, and public websites for educational purposes, found that health care professionals generally viewed these digital solutions as supportive of health literacy. However, the research revealed a reluctance among medical professionals in German primary care to use digital devices for health information and education [43]. Further, 1 study from Austria [35] and 1 study from Germany [29] found that, in general, GPs primarily use eHealth for communication. According to 2 German studies, telemedicine adoption is increasing, driven by the need to improve access and efficiency [74]. There is high satisfaction for telemedical applications, particularly asynchronous ones [75], and German postgraduate trainees in family medicine believed telemedicine’s potential is underused [70]. Additionally, email consultations have become significant in 2021, according to a Danish study [71]. Among Swedish studies, 1 study explored Swedish primary care staff’s experiences with a digital communication platform that used automated patient interviews and asynchronous chat [42]. While staff initially had concerns, after an adjustment period, they found the platform helpful in specific clinical contexts, particularly when combined with continuity of care. Still, they emphasized the need to consider its implementation and use carefully [42]. Further, 2 other Swedish studies showed that, despite low reported use in some contexts, GPs generally show positive attitudes toward telemedicine and DHTs [44,48]. According to 1 Norwegian study, remote consultations have significantly impacted general practice and are considered a permanent fixture in primary care, necessitating proactive management to maintain health care quality and sustainability [32]. In addition, a Dutch study by Peeters et al (2023) [77] showed that e-consultation, an asynchronous digital communication tool between GPs and hospital specialists, has emerged as a potential solution to address challenges from shifting tasks from specialized hospital care to primary care, having positive impacts on the quality of care, collaboration between health care providers, and care accessibility. In Swedish and Danish studies, VCs showed variable adoption due to differing interpretations of the technology’s advantages, and they were not perceived as more effective than physical consultations, despite positive GP attitudes [68,76]. The COVID-19 pandemic catalyzed an unprecedented digital transformation [57]. Dutch practices reported intensifying eHealth use, with telephone consultations particularly well received in low-income neighborhoods [46,81]. Sweden’s rapid adoption of the “Always Open” digital primary care system demonstrated health care systems’ capacity to adapt during a crisis, and factors contributing to successful adoption included professional ethics, financial incentives, and patient needs [54]. At the same time, concerns focused on potential conflicts between standardization and decentralized management models [54]. Two Dutch studies reported that GPs intended to continue using eHealth as extensively as, or even more extensively than, before the pandemic [73,78]. However, several other studies found that only a minority of GPs planned to maintain increased eHealth use post pandemic [46,67,74,75]. This was further exemplified by 1 Swedish study showing high intention but low actual telemedicine use (n=198, 11% regularly) [48]. Moreover, 1 French study found that the adoption of eHealth innovations depends on their alignment with doctors’ professional culture, and that, despite the potential benefits, recent digital initiatives, particularly those emerging during and after the COVID-19 crisis, face resistance from some GPs who view medicine as an “art” [58]. Geographic disparities reflect differences in infrastructure and policy, according to a pan-European study: Northern and Western European countries demonstrated greater digital maturity than Eastern European countries [38]. Adoption factors in 2 German studies included GPs’ sociodemographic characteristics, practice-related variables, and digital maturity [56,66]. Furthermore, group practices used eHealth more than solo practitioners [46], and larger practice size, higher eHealth readiness, and younger age predict greater information and communication technology use and AI openness among GPs [41,50,88]. Urban practices in Sweden and Germany reported higher confidence than their rural counterparts, who face connectivity challenges [63,68], and medical assistants play a crucial role in facilitating adoption [47].

#### GP Experiences: Benefits, Workflow Changes, and Patient Interactions

##### Perceived Benefits

GPs identified efficiency gains as the primary benefit, though experiences varied by technology maturity and context [31,34]. Enhanced patient access emerged consistently among Norwegian GPs, with improved accessibility for routine follow-ups and improved care delivery to previously underserved rural populations [32,59,72]. Additionally, digital platforms enabled 24/7 patient contact [54], reduced administrative time [44,54,63], and enhanced care continuity through digital communication channels [36,42,44,67,71]. Benefits included improved patient compliance, mobility, education, and self-efficacy [29,35,40,55]. Additionally, GPs agreed that digital consultation options reduced unnecessary in-person visits for minor complaints [34,63]. In 1 study exploring digital health interventions for hypertension, GPs recognized the benefits of these technologies, including more accurate blood pressure data and increased patient engagement in self-management [30]. In 1 Belgian study, GPs noted digital self-tracking fostered more informed patient-physician dialogues and effectively reduced provider workload [37]. Telemedicine consultations offered fewer necessary face-to-face visits, greater flexibility and accessibility, enabling tailored clinical pathways and better documentation [32,74,78]. Specifically for written consultations, studies reported positive experiences with digital patient dialogues, citing benefits such as improved patient safety through self-reported histories [67] and the ability of e-consultations to signal GP presence even in silence [79]. Moreover, 1 study found that VCs can be time-saving [68]. Interprofessional consultations were seen as learning opportunities with improved care delivery and communication efficiency [77]. EMR systems were associated with administrative time savings, improved documentation, and better patient access [90,91], while patient access to EMRs enhanced engagement, health literacy, autonomy, and convenience [90,92]. For LTCs, digital tools enabled faster clinician access and remote monitoring [60], reduced time demands, improved care quality and continuity, strengthened patient autonomy, health literacy, and self-efficacy, and reduced health care professional workload [51,82]. AI was perceived as having the potential to improve efficiency, documentation, diagnostic support, time management, administrative burden, and professional self-reflection [33,84-87,89].

##### Workflow Disruptions

Digital implementation consistently disrupted established workflows [71], requiring 6‐12 month adaptation periods [40,54,87]. The “double documentation” phenomenon affected GPs, while managing online record access created an unmeasured workload by requiring the crafting of “patient-friendly” notes [91-93]. Moreover, in some Danish studies, GPs spent less time per individual digital consultation, but handled higher volumes, resulting in no net time savings [71,76,83]. Sensory challenges emerged with telemedicine. Danish GPs described VCs as “amputated” experiences lacking tactile examination [76]. In Norwegian and other Danish studies, GPs also reported altered sensory conditions in VCs, raising concerns about missing important information and difficulties in patient assessment [32,59,72,80]. To compensate, GPs ask more questions, repeat advice, and use meta-communication [69,80]. Role redefinition occurred across teams, with medical assistants assuming expanded responsibilities [47].

##### Patient Relationship Impact

Digital technologies fundamentally altered doctor-patient dynamics [70,71]. Written communication altered power dynamics, with patients having more time to formulate questions, but with GPs experiencing increased pressure to provide detailed written responses [71,79,83]. Some GPs felt that digital tools undermined relationship-based care [34,47] and noted reduced interpersonal communication with patients [49,51,57], decreasing opportunities for rapport-building and detecting nonverbal distress cues [30], as well as concerns about the potential misinterpretation of text-based communication, inappropriate use by some patient populations [42], and increased health obsession, anxiety, and medicalization [36,37,63]. Furthermore, patient empowerment proved double-edged: digital self-tracking increased engagement but risked health obsession [37], while online record access generated “worried well” consultations with worries about patient safety, increased anxiety, and misunderstandings arising from patients viewing their records [30,91-93]. The digital divide disproportionately affected vulnerable populations, prompting practices to maintain parallel pathways [32]. While younger patients demonstrated greater trust in digital consultations, older adult patients felt less comfortable [57,74].

### Impacts on Workload, Efficiency, and Ethical Concerns

#### Workload and Efficiency Paradoxes

Digital technologies showed mixed effects on workload, often increasing burden initially before potential efficiency gains emerge. Although telemedicine consultations could save time, the overall workload frequently increased due to higher consultation volumes and asynchronous communication [30,63,74,91,93]. Additional workload arose from boundary erosion and “invisible work,” including after-hours connectivity, technical troubleshooting, algorithm review, patient-oriented language adaptation, and ongoing learning [32,62,70,73,75,79,85,91]. Alert fatigue from excessive CDSS notifications [84,87] and notification burden from patient portal messages [91,92] further increased administrative demands. Efficiency gains depended on effective integration with EHR systems [45], adequate training [39], and practice context, with greater efficiency observed in group practices [46] and digitally mature settings [66]. Patient access to EHRs also increased workload through query management and monitoring demands, thereby reducing efficiency and increasing the risk of burnout [91-93].

#### Ethical and Professional Concerns

Data privacy and security emerged as the predominant concern [33,34,41,48,49,55,70], with some GPs ranking data protection as their top priority. Algorithmic transparency worried practitioners, with concerns about “black box” algorithms [85], liability uncertainties when following AI recommendations [89], and skepticism about AI replacing core functions requiring empathy and communication [33]. Professional autonomy was perceived as threatened by standardization pressures, with Danish GPs describing themselves as “reduced to technicians” [76] and technical issues diminishing patient trust in the intervention [62]. Equity issues permeated implementation—the “inverse care law” manifested digitally, with populations most in need facing significant access barriers [57,68,74]. GPs reported moral distress when digital-only pathways excluded vulnerable patients [32], and struggled to balance efficiency gains with equitable access [62,78]. Concerns about declining clinical acumen were also raised [32], along with changes in documentation practices, including reduced candor [91]. In studies on LTC management, concerns about capturing the nuances of complex conditions and their suitability for complex patients were raised [57,60]. In 1 study, hygiene concerns regarding shared electronic devices were expressed [43]. Besides, the unclear long-term availability of digital interventions was addressed [62].

### Barriers to Implementation

Frequently mentioned barriers to implementation or adoption were the complexity of implementation and limited integration or compatibility with existing systems [34,41,43,49,57,82,84,88], poor applicability and design [57,60,77], technical and organizational obstacles [31,38,62,78,85,88], workflow adjustments and time constraints [34,49,71], costs and inadequate reimbursement [55,66,78,85,88], and training efforts [66]. Adoption decisions were also influenced by data protection as a critical barrier [50,55,70,90]. System interoperability problems were noted by a majority of UK and German GPs in 2 studies [50,66]. Another barrier mentioned was insufficient network coverage [82] and a lack of adequate broadband internet access [75]. Organizational barriers centered on a lack of skills [78], inadequate training (n=1044, 91.5%) of GPs who lacked formal digital health education [39], and change resistance, particularly in mixed-age practices [57]. Digital literacy was identified as a barrier to implementation, as it varied dramatically by age, with younger GPs showing a higher affinity for using digital tools [43,49,57,74,78,82]. In another study, staff felt that the provided training was not tailored to their individual needs and did not adequately support them in planning and understanding the intervention’s implementation [62]. Furthermore, observed barriers included perceived passive roles or uncertainty among GPs in delivering the intervention and the absence of a feedback loop [62]. Some GPs also reported a lack of comprehensive information about DHAs, with only a minority feeling competent to advise patients on their use [55]. Patient-related barriers reflected disparities in capability and preference, including digital affinity, motivation, age (younger patients demonstrated greater trust in digital consultations than older patients [60,93]), and health literacy [43,49,56,78,82]. Furthermore, systemic barriers included liability uncertainties (eg, GPs worried about telemedicine malpractice) [70,76] and evidence gaps, with many digital tools lacking robust outcomes data [41,57].

### Policy Recommendations for Sustainable Digital Health Integration

#### Infrastructure and Standards

Studies highlighted the early involvement of support staff to ensure alignment with existing workflows and professional needs [30,39,58,62,65,77]. Universal broadband access (minimum 100 Mbps) was identified as foundational [67,74] alongside mandated interoperability standards and reliable technical platforms to enable system integration [35,49,50,68]. Compatibility with existing systems and ease of use for health care providers were consistently emphasized [45,49,52,53,57,88], while gamification elements supported patient engagement [82]. Higher digital usage was observed in practices with dedicated IT support and mechanisms to address technical issues [31,39-41,43,50,51,54,57,88], suggesting that shared regional support centers could serve multiple practices efficiently [49]. Alignment with practice priorities and clear role delineation across care levels were also critical [52,65,77]. Sustained use of the Always Open platform reflected the importance of balancing standardization and flexibility and demonstrated system adaptability to local needs during crisis conditions [54]. Digital lifestyle coaching for patients with type 2 diabetes was feasible when roles were clearly defined and integration into EHRs was straightforward [45].

#### Financial Sustainability

Sustainable funding must recognize actual implementation costs—current reimbursement does not cover actual expenses [34,54-57,73,75,88]. Successful implementation required addressing cost concerns with better funding or incentives [34,45,52,54,70,73,88].

#### Workforce Development

Comprehensive digital health education must begin in medical schools to address the current gap [39,70]. Successful implementations of digital health technologies also required whole-team training on the benefits and applications of eHealth, with regular protected learning time recommended to increase adoption and confidence in using digital tools [40,41,49,54,55,57,62,65,68,77,78]. Digital champions in practice were also seen as beneficial, achieving higher adoption rates [36,49].

#### Governance and Quality

Adaptive regulatory frameworks must balance innovation with safety through proportionate, risk-based oversight [38,42,55,56,64,65,85]. Professional bodies need to establish digital consultation standards [71,76,91], collaborate with health insurance companies [82], and clarify liability when AI influences decisions [33,84,85,88,89]. Quality metrics require reimagining—traditional measures poorly capture digital care effectiveness, but clear, evidence-based benefits, especially for patient care, were seen as essential [45,50-52,57,60,62,74,77,82,83,88].

#### Equity Assurance

Digital inclusion requires proactive strategies: “digital navigators” to support vulnerable patients, considering patients’ digital literacy and tools that are adaptable to users [31,43,49,51,56,57,61], device lending programs [51], preserved nondigital pathways that maintain human contact in care delivery [31,44,46,53,59,60] and continuity-of-care relationships [31,44,59,60,72]. In addition, there is a need for condition-specific tools [30,45,52,57]. Digital consultations can be established for patients with straightforward issues, prioritizing in-person visits for new patients, complex diagnoses, and vulnerable populations [31,46,60,72,74,81]. GPs also underscore the importance of shared decision-making, with the doctor leading and the patient deciding [30,36,51,57,59,63]. Quality standards must ensure traditional access routes receive equal investment [31,44,46,60,72], while cultural adaptation ensures tools reflect diverse population needs [38,43,52,57,81]. Additionally, interventions should complement existing care delivery models and take a holistic approach to patient management [45,52,88]. For AI systems, key requirements include AI transparency (eg, probabilistic differential diagnosis displays), guaranteed data security, and comprehensible algorithms, with AI systems needing validation, accuracy, and proven reliability [84,85,87].

### TAM-NPT Effect-Direction Analysis

#### Overview

TAM captures GPs’ perceptions of digital health technologies. Perceived usefulness captures the extent to which GPs feel that a digital technology meaningfully improves their clinical work or patient care. Perceived ease of use reflects how effortless and intuitive GPs find it to learn and use a digital technology in everyday practice. Behavioral intention represents GPs’ stated willingness and plans to use a digital technology in the near future. Actual use indicates the degree to which a digital technology is in fact used in routine GP practice, beyond stated intentions. In contrast, NPT captures implementation processes based on: coherence (do GPs understand why this technology is needed?), cognitive participation (do GPs buy into using it?), collective action (can GPs do the work to implement it?), and reflexive monitoring (do GPs evaluate if it is working?). Table 6 presents the effect direction plot synthesizing TAM and NPT outcomes across all 65 studies, stratified by technology type. The full TAM-NPT percentage breakdown is provided in Tables S1 and S2 of Multimedia Appendix 7.

**Table 6.** TAM^a^-NPT^b^ effect direction plot across digital health technologies: effect direction symbols and thresholds. TAM constructs: perceived usefulness (expected benefit for GPs^c^), perceived ease of use (effort required to use the technology), behavioral intention (stated intention to use), actual use (reported use in practice). NPT constructs: coherence (understanding why the technology is needed), cognitive participation (buy-in and engagement), collective action (ability to integrate into daily work), reflexive monitoring (evaluation of whether it works in practice). Percentages show the dominant response category for each construct.

	AI/CDSS^d^ (n=7) Ø MMAT^e^=83% countries: 4	DHT^f^ (n=36) Ø MMAT=83.89% countries: 13	EMR^g^ (n=4) Ø MMAT=95% countries: 2	TM^h^ (n=18) Ø MMAT=85.56% countries: 6	Overall (n=65) Ø MMAT=87% countries: 14
TAM					
Perceived usefulness	↑↑^i^ (85.7% Y^j^)	↑↑ (83.3% Y)	↑↑ (100% Y)	↑↑ (88.9% Y)	↑↑ (86.2% Y)
Perceived ease of use	↓^k^ (57.1% N^l^)	↓↓^m^ (75% N)	↓ (50% N)	↓ (55.6% N)	↓↓ (66.2% N)
Behavioral intention	↔^n^ (85.7% P^o^)	↔ (88.9% P)	↔ (100% P)	↔ (72.2% P)	↔ (84.6% P)
Actual use	↑↑ (100% Y)	↑↑ (94.4% Y)	↑↑ (100%)	↑↑ (100% Y)	↑↑ (96.9% Y)
NPT					
Coherence	↔ (71.4% P+U^p^)	↔ (52.8% Y, 38.9% P+U)	↑^q^ (75% Y)	↔ (50% Y, 50% U)	↔ (50.8% Y)
Cognitive participation	↓↓ (71.4% N)	↔ (58.3% P)	↔ (50% P)	↓ (61.1% P+U, 33.3% N)	↔ (47.7% P)
Collective action	↔ (100% P)	↔ (100% P)	↔ (100% P)	↔ (100% P)	↔ (100% P)
Reflexive monitoring	↑ (71.4% Y)	↑ (63.9% Y)	↑↑ (100% Y)	↑ (61.1% Y)	↑ (66.2% Y)

a TAM: technology acceptance model.

b NPT: normalization process theory.

c GP: general practitioner.

d AI/CDSS: AI/clinical decision support systems.

e MMAT: Mixed Methods Appraisal Tool.

f DHT: digital health tools.

g EMR: electronic medical records.

h TM: telemedicine.

i ↑↑ strong positive: >80% yes, <10% no.

j Y: yes.

k ↓ negative: >30% no, <30% yes.

l N: no.

m ↓↓ strong negative: >60% no, <10%.

n ↔ mixed: >50% partial or unclear.

o P: partial.

p U: unclear.

q ↑ positive: 60%‐80% yes.

#### TAM-Analysis

For perceived usefulness, GPs rated the overall outcome across all technologies very positively, with few reservations. A total of 86.2% (56/65) of responses show that family doctors clearly recognize the benefits of these technologies. A further 12.3% (8/65) see at least partial benefits. There is no absolute rejection, and only 1.5% (1/65) rated it as unclear. Perceived ease of use across all technologies indicated that user-friendliness is the most significant weakness. Further, 66.2% (43/65) rated it negatively, and a further 20% (13/65) found it only partially positive. Behavioral intention indicated that, despite recognized benefits, only 4.6% (3/65) of responses stated a willingness to use digital health technologies. Additionally, 84.6% (55/65) exhibited only partial behavioral intention, whereas 10.8% (7/65) reported no intended use. Despite low ease of use and low intention, actual usage is 96.9% (63/65), with only 3.1% (2/65) reporting partial use.

#### NPT-Analysis

Overall, coherence across all technologies was rated as clear by 50.8% (33/65). Another 12.3% (8/65) showed partial understanding, 32.3% (21/65) rated it as unclear, and only 4.6% (3/65) explicitly rejected it. For cognitive participation, the results indicate that only 4.6% (3/65) of ratings are positive, whereas 47.7% (31/65) are only partially supportive of the use of digital technologies. In addition, 33.8% (22/65) negative ratings of GPs’ buy-in are observed. For collective action, 100% (65/65) of ratings indicated only partial ability of GPs to do the work required to implement digital health technologies. For reflexive monitoring, the assessments were most positive: 66.2% (43/65) judged that GPs clearly evaluate whether the implemented technologies are working in practice, and a further 33.8% (22/65) indicated that this evaluation occurs partially; no assessment suggested that such evaluation is absent or cannot be determined.

#### Cross-Construct Correlations

Analysis of TAM-NPT interactions revealed several correlations. (1) Regarding inverse usability-usefulness relationship, technologies with the highest perceived usefulness ratings paradoxically had the poorest ease-of-use scores. This inverse correlation was most pronounced in EMRs (4/4, 100%, useful and 2/4, 50%, explicitly not easy to use) and DHTs (30/36, 83.3% useful and 27/36, 75% not easy to use). (2) Regarding disconnection between use and usability, actual use (63/65, 96.9%) showed no correlation with perceived ease of use, which was 0% (0/65) unequivocally positive, suggesting that technology adoption in primary care is driven by factors other than usability. (3) Regarding implementation stage breakdown, NPT analysis revealed a progressive decline in successful implementation: regarding early stage (coherence), 50.8% (33/65) of GPs understand why technologies are needed; regarding mid stage (cognitive participation), only 4.6% (3/65) demonstrate genuine buy-in; regarding late stage (collective action), 0% (0/65) achieve full implementation; and regarding evaluation (reflexive monitoring), 66.2% (43/65) actively evaluate outcomes despite implementation challenges. The analysis also shows that, despite 96.9% (63/65) of studies reporting actual technology use, 100% (65/65) across all technology types reported only partial success in collective action, indicating incomplete workflow integration.

#### Technology-Specific Variations

AI or CDSS showed the lowest cognitive participation (5/7, 71.4%, no), indicating significant resistance to AI integration despite recognizing its usefulness (6/7, 85.7% yes). DHTs demonstrated the most severe usability challenges (27/36, 75% no for ease of use) while maintaining 94.4% (34/36) actual use, exemplifying forced adoption without user-centered design. Despite achieving 100% (4/4) perceived usefulness, EMRs demonstrated poor ease of use and the greatest negative impact on GP satisfaction, reflecting the burden of mandated documentation. Telemedicine showed the most variable patterns across constructs, with higher behavioral intention scores (2/18, 11.1% yes) than other technologies, possibly reflecting its voluntary adoption during COVID-19.

To assess the robustness of these patterns, we conducted two sensitivity analyses using side-by-side comparison tables (Tables S7-S10) in Multimedia Appendix 7: one excluding three large multicountry surveys with potential respondent overlap and one excluding four studies with MMAT scores of 60%. Across all TAM and NPT constructs, changes in category percentages remained below 2%, and effect-direction arrows were unchanged for both the affected technology subgroups and the overall sample. These findings indicate that the observed “implementation paradox” and the main effect-direction patterns are robust to plausible levels of double counting and to the exclusion of lower-MMAT studies.

#### Geographic and Contextual Patterns

Country-level analysis revealed concentrated specialization rather than diverse implementation. Germany accounted for the largest share of studies on DHTs (11/36, 30.6%). Denmark, Germany, and the Netherlands emerged as telemedicine hubs, with 27.8% (5/18) and 22.2% (4/18) of studies conducted there, respectively. The United Kingdom had the most balanced technology coverage across all four categories, and the Nordic countries (Denmark, Norway, and Sweden) showed marginally better telemedicine implementation outcomes. However, geographic interpretation is limited by the sample distribution: 93.8% (61/65) of studies originated from Western and Northern Europe, with minimal representation from Southern Europe (3/65, 4.6%) and Eastern Europe (1/65, 1.5%), thereby preventing robust regional comparisons.

Supplementary TAM-NPT tables summarizing the number of single-country studies, technology mix, and effect-direction arrows by construct for each country are provided in Tables S3-S5 in Multimedia Appendix 7. This map is explicitly intended as a heuristic, reviewer-requested overview rather than a full comparative policy analysis, and we caution that cross-country contrasts are tentative, given sparse, temporally heterogeneous, and technology-specific evidence in several countries. For the subset of studies reporting discrete, directly comparable quantitative percentages, observed ranges by outcome domain are provided in Table S6 in Multimedia Appendix 7.

## Discussion

### Principal Findings

This systematic review synthesized evidence from 65 studies involving 24,994 GPs across 14 European countries and found a consistent pattern of high perceived usefulness but low perceived ease of use for digital health technologies in general practice. In the provided overall TAM analysis, all effect directions for perceived usefulness were positive (56/65, 86.2%). In contrast, responses regarding perceived ease of use were predominantly negative (43/65, 66.2%) or neutral (22/65, 33.8%), and behavioral intention showed only a modest positive trend (3/65, 4.6%). At the same time, the overall NPT analysis indicated that although GPs often engage with digital tools, the effect directions for cognitive participation (3/65, 4.6%, positive) and collective action (65/65, 100%, partial) were frequently negative, implying low buy-in to specific tools and limited embedding in everyday work routines. These findings align with the “promise and paradox” of digitalization described in the broader primary care literature, where digital technologies are widely deployed but often increase complexity and cognitive load rather than simplifying practice [105-108]. At the same time, the overall high actual use observed (63/65, 96.9%) in this review, despite low perceived overall ease of use (22/65, 33.8%, neutral, and 43/65, 66.2%, negative) and modest behavioral intention (84.6% partial and 10.8% negative), suggests that digitalization in European general practice is now largely system-driven rather than purely clinician-driven and that it is pervasive and largely mandatory but only partially normalized from the perspective of frontline GPs. The observed pattern of high overall reflexive monitoring (43/65, 66.2%) is consistent with this interpretation.

### Adoption Patterns, Digital Maturity, and Temporal Concentration

The temporal distribution of studies (52/65, 80%, published between 2020 and 2024) demonstrates that the COVID-19 pandemic period dominates the empirical evidence base on GP experiences with digitalization. This concentration mirrors broader analyses of telehealth and digital primary care, which report accelerated digital adoption during the pandemic, followed by heterogeneous trajectories of stabilization or partial rollback [108,109]. The fact that 55.4% (36/65) of all included studies focused on generic DHTs and that only a minority addressed AI (7/65, 10.8%), EHRs (4/65, 6.2%), or telemedicine (18/65, 27.7%) suggests that, for most GPs, digitalization is experienced as a diffuse, system-level change rather than as isolated innovations. This is consistent with cross-national surveys showing that basic eHealth functions are nearly universal in European primary care, while more advanced functions and AI-based tools remain unevenly implemented [38,106,110]. Geographically, 47.7% (31/65) of the included studies originated from Germany and the United Kingdom, with additional clusters in the Netherlands (7/65, 10.8%), and Scandinavian countries (17/65, 26.2%). This pattern parallels multicountry digital maturity assessments that identify Northern and Western Europe as early adopters with higher digital readiness, while Eastern and some Southern European countries lag [105,111]. The fact that this review found relatively few studies from Eastern Europe likely reflects this underlying maturity gap and underscores that current knowledge about GP perspectives is biased toward more digitally advanced health systems [112]. Our assessments on digital maturity in general practice also found that interoperability and evaluation of best practices were the weakest dimensions, and that rural settings and smaller practices were consistently associated with lower digital maturity. These patterns are consistent with a general finding that group practices and digitally “mature” practices report more positive experiences and higher use, whereas rural, resource-constrained settings struggle with connectivity, integration, and sustained adoption [105].

### Workload Paradoxes and Efficiency Claims

Across technologies, this review found that while digital technologies may shorten individual encounters, GPs more often reported increases in workload than reductions, particularly with telemedicine, patient portals, and electronic messaging. In the effect direction analysis, most studies on workload reported neutral or adverse effects, indicating perceived time pressure, additional documentation, and “hidden” tasks such as managing patient portal messages and reconciling duplicate documentation across parallel systems. These results are consistent with a pre–COVID-19 systematic review of eHealth use in general practice, which found that digital communication and portal functions frequently increased overall workload despite potential gains in individual encounters [2]. They are also consistent with modeling work on digital-first access in UK general practice, which estimated that online and VCs would increase total GP workload by 3%‐31% in typical scenarios unless consultation length and demand are carefully managed [113]. In addition, the TAM-NPT pattern identified high perceived usefulness (56/65, 86.2%), very low ease of use (0/65, 0%), low buy-in (3/65, 4.6%), but near-universal use (63/65, 96.9%), which fits with these workload findings. It suggests that GPs often experience digitalization as an obligation embedded in regulatory, reimbursement, and organizational requirements, rather than as a voluntary efficiency gain. This interpretation aligns with broader analyses of “work as imagined vs work as done” in digital health, which emphasize the gap between policy narratives of simplification and clinicians’ experiences of increased complexity and administrative “shadow work” [108]. The dominance of negative or neutral effect directions for workflow and workload in this review adds empirical precision to the more general concerns reported in umbrella reviews of digital health technologies for health workers, which describe increases in mental workload, multitasking, and documentation burden associated with EHRs and related tools [12,15]. At the same time, a subset of studies in this review reported positive workload effects for well-integrated asynchronous tools and for specific use cases, such as chronic disease monitoring or asynchronous specialist consultations [39,51,52,57,60]. This mirrors external evidence that digital interventions can reduce workload when they replace, rather than add to, existing processes and when they are matched to appropriate clinical tasks [6,107].

### Doctor-Patient Relationship, Access, and Inequities

This review also found that digital tools improved access and continuity for specific patient groups but raised concerns about depersonalized care and uneven benefits. Numerically, most studies addressing continuity and access reported positive effects, whereas those focusing on relational quality and communication nuances more often reported neutral or adverse effects. This nuanced pattern aligns closely with qualitative studies of digital primary care in Scandinavia and elsewhere in Europe, where clinicians describe digital platforms as enhancing convenience and follow-up while limiting nonverbal communication and making complex conversations more challenging [114]. Furthermore, this review found that GPs consistently perceived older patients, people with low digital literacy, and those in rural or socioeconomically deprived areas as less likely to benefit from digital services. Throughout studies, GPs explicitly linked digital access channels to “digital queues” that are easier to navigate for younger, better-resourced patients, raising concerns about a widening inverse care law. These perceptions are strongly supported by a scoping review of digital health technologies and inequalities, which concludes that digital tools tend to reinforce existing social gradients in access and outcomes unless counterbalanced by targeted inclusion measures [21]. National-level analyses of remote consultation uptake in general practice likewise show higher digital use in less-deprived populations and lower uptake among older and more disadvantaged groups, even where services are universally available [7,115]. Against this background, the observed effect-direction patterns strengthen the argument that equity considerations must be integral to digital strategy in European general practice, rather than an afterthought.

### AI, Decision Support, and Trust

Only a small fraction of the included studies in this review focused on AI or CDSS (7/65, 10.8%). The effect-direction analysis for these technologies showed a distinctive profile: GPs frequently rated perceived usefulness as high (6/7, 85.7%) but expressed negative or neutral directions for perceived ease of use (3/7, 42.9%, neutral and 4/7, 57.1%, negative), behavioral intention (6/7, 85.7%, neutral and 1/7, 14.3%, negative), coherence (2/7, 28.6%, positive and 5/7, 71.4%, neutral), and collective action (7/7, 100%, neutral). This asymmetry mirrors findings from recent scoping and systematic reviews of AI-based CDSS in primary care, which report that while such systems can improve process measures or diagnostic accuracy in controlled settings, clinicians express persistent concerns about transparency, responsibility, and the potential for over-reliance [4]. A systematic review of the benefits and harms of algorithmic decision-making systems in health care similarly concludes that evidence for clear clinical benefit remains limited and heterogeneous [18]. In contrast, perceived risks related to bias, opacity, and workflow disruption are repeatedly documented [18].

Moreover, these results indicate difficulties in understanding when and how to use these systems, as well as in integrating them with existing decision-making practices. This pattern aligns with studies on trust in AI-based CDSS, which find that lack of explainability, misalignment with clinical reasoning, and inadequate training are significant barriers to adoption [17,19]. The numerical imbalance between high perceived usefulness and low perceived ease of use, and the coherence in this review, therefore, reinforce the interpretation that AI in primary care remains at an early stage of normalization: GPs see potential value but lack the infrastructure, governance, and experiential reassurance needed to embed these tools in routine care.

### Implementation and Training

The cross-cutting barriers identified include poor interoperability, fragmented systems, lack of training, inadequate reimbursement, and limited local support, and are described in multiple implementation-focused reviews and policy reports [9,10]. The combined TAM-NPT analysis reveals that 100% (65/65) of implementations remain partial, regardless of technology type, setting, or perceived benefits, which indicates systemic issues in how digital health is conceived, deployed, and supported in primary care settings. In combination with the high actual overall usage (63/65, 96.9%), this indicates that technologies are being adopted without full integration. Across all technologies, a lack of organizational support and training was found. GPs in the included studies described limited formal training, lack of protected time to learn new systems, and inadequate local support as key barriers to effective use. These findings resonate with European-level surveys showing that a majority of health care professionals report little or no structured digital skills training, and that available training is often perceived as insufficient or poorly aligned with practical needs [116]. They are also consistent with conceptual and policy papers arguing that digital competencies should be embedded into undergraduate and postgraduate curricula and accompanied by continuous workplace-based learning [117].

### System Conditions

Regarding system conditions, this review found fragmented platforms and poor interoperability as significant sources of frustration, which negatively affected workflow integration and perceived system quality. OECD (Organisation for Economic Co-Operation and Development) and World Health Organization reports on EHR and telehealth deployment identify similar issues, noting that lack of common standards, fragmented vendor landscapes, and unclear governance undermine both clinician acceptance and the realization of potential benefits [117-119]. The convergence between these policy-level analyses and the quantitative patterns of effect direction observed here strengthens the conclusion that many of the problems GPs report are systemic rather than individual: they arise less from resistance to innovation and more from the design, implementation, and funding of digitalization.

### Implications

By combining TAM and NPT with effect-direction analysis, this review provides a structured, quantitative overview of how European GPs experience digitalization across multiple outcome domains. The predominance of positive effect directions for perceived usefulness, contrasted with negative or neutral directions for ease of use, behavioral intention, cognitive participation, and collective action, suggests that digitalization in its current form creates a “compliance without conviction” dynamic: GPs use digital tools extensively because they must, not because these tools reliably make their work easier or their care better. External evidence on workload, burnout, inequalities, and implementation barriers supports this interpretation and indicates that similar patterns are being observed in other high-income primary care systems [2,9,21,120]. Taken together, this review and related literature suggest that the central challenge for European general practice is no longer whether to digitalize, but how to redesign systems so that digital tools genuinely support, rather than strain, frontline clinical work [111,116,117,121]. This review suggests that, for the digital transformation of general practice, digital technologies should be embedded in person-centered, team-based models of care, with iterative adaptation informed by user feedback and real-world evaluation. For GPs, this implies prioritizing (1) interoperable, user-centered systems that reduce duplicate documentation and align with existing workflows; (2) reimbursement and staffing models that recognize additional digital workload and support roles such as digital navigators or dedicated triage staff; (3) structured education on digital health and AI at undergraduate and postgraduate levels, combined with ongoing protected time for practice-based training; and (4) governance approaches that balance innovation with privacy, safety, and equity, including explicit standards for teleconsultation quality and AI use in clinical decision-making.

### Future Research

Future research should build on these quantitative patterns by (1) conducting longitudinal and experimental studies that can disentangle short-term disruption from long-term adaptation; (2) expanding the evidence base to underrepresented regions and practice settings, especially in Eastern and Southern Europe; and (3) rigorously evaluating hybrid care models that purposefully combine digital and face-to-face contacts, including their equity and economic impacts. Such work will be essential to move from documenting the paradoxes of digitalization to identifying concrete design, policy, and training strategies that allow digital tools to genuinely support GP-centered, equitable, and sustainable primary care in Europe [6,107,122].

### Strengths and Limitations

This systematic review provides a comprehensive understanding of digitalization in European general practice, supported by several key strengths. We adhered to the PRISMA 2020 guidelines and used a robust, innovative search strategy across 8 databases in combination with Elicit AI [26] to identify gray literature. A significant strength is our extensive and diverse evidence base, which synthesizes data from 24,994 GPs across 14 European countries and encompasses a rich mix of qualitative, quantitative, and mixed methods studies. The predominance of qualitative and exploratory designs is appropriate for examining emerging technologies, where understanding contextual factors and user experiences is critical. While large quantitative surveys (eg, the pan-European survey with 9196 respondents [38]) allow for the identification of broad trends, qualitative studies with smaller samples provide rich contextual understanding of the mechanisms underlying these trends. Mixed methods designs enabled both the validation of trends using quantitative data and the exploration of underlying mechanisms through qualitative inquiry, providing complementary perspectives. This methodological diversity and consistency across methods (interviews, focus groups, and content analysis) strengthen the comparability of findings, as many studies independently identified similar themes in their qualitative data, adding confidence to the common denominators and enabling triangulation of findings. The included multicountry study [38] contained data from Turkey, which falls outside our strict geographic eligibility criteria. However, this study was retained for pragmatic reasons: Turkey represented only 1 of 31 countries surveyed (approximately 1/31, 3.2%), and the aggregate data could not be disaggregated to exclude Turkey while preserving the remaining European data. As this large quantitative study strengthens the evidence base, its inclusion outweighs the minor geographic deviation. Importantly, the findings from this study align with patterns observed in studies conducted exclusively within our defined geographic scope, and its inclusion does not materially affect the review’s overall conclusions. It is also worth noting that there may be some overlap (eg, the pan-European survey [38] may include respondents who also participated in country-specific studies). To assess this, we repeated the TAM-NPT effect direction analysis after excluding the three largest DHT surveys [38,46,65]; category percentages changed by less than 2 percentage points, and the overall patterns remained unchanged, indicating robustness to plausible double-counting. We also repeated the qualitative synthesis after excluding the four studies with MMAT scores of 60%; no changes in the direction of conclusions were observed in any outcome domain, suggesting that our main findings are robust to quality restrictions as operationalized by MMAT. Given the heterogeneity of the included studies, a narrative synthesis was appropriately chosen to integrate findings, reinforcing credibility through thematic consistency across independent datasets.

Despite these strengths, our review has several limitations. A primary constraint is its Eurocentric focus, as all included studies are from Europe, thereby limiting the generalizability of the findings to global contexts, particularly the Global South. Besides, representation from Eastern European countries remains limited. Furthermore, the language restriction to English and German may have introduced language bias. Further, 6 records were excluded solely due to language (1 Spanish, 3 French, 1 Italian, and 1 Dutch study; identified in Scopus, Web of Science, and SpringerLink), which may have led to a slight underrepresentation of evidence from these countries and related themes. This pattern is summarized in Multimedia Appendix 3. Additionally, the review predominantly captures the perspectives of GPs, with patient views and those of other crucial health care stakeholders underrepresented. Furthermore, although the search was broad and informed by PRESS 2015, the limited and nonuniform use of controlled vocabulary (MeSH/EMTREE) and proximity operators across heterogeneous database interfaces may have reduced search sensitivity to some extent, despite our efforts to mitigate this through extensive synonyms and multiple databases. Additionally, the search strategy was not formally peer-reviewed, and the study selection and data extraction processes did not involve a fully independent assessment by reviewers, which may have introduced bias. Methodologically, the evidence base relies heavily on surveys and observational designs, with no randomized controlled trials, which limits the ability to infer causality. Data collected through self-reports are also susceptible to bias. Additionally, a notable limitation across the quantitative studies was low response rates typical of GP surveys, which may have introduced nonresponse bias. Finally, we used Elicit AI [26] to support literature searching and data handling. As the underlying models and software builds are not disclosed and may change over time, and because full use of our shared Elicit workspaces requires Pro-tier access, exact replication of the AI-assisted components of the search may be limited, although the core database searches are fully reproducible from the reported strings and sources.

### Conclusions

This systematic review of 65 studies, including 24,994 GPs, is, to our knowledge, the first to offer a Europe-wide synthesis of GPs’ experiences with digitalization across EHRs, telemedicine, DHTs, and AI-based tools, using a combined TAM-NPT effect-direction framework. By quantifying an implementation paradox—high perceived usefulness and near-universal use in the absence of positive usability ratings or full workflow integration—it extends earlier reviews that primarily examined patient outcomes, system-level metrics, or single digital interventions. For policy and practice, the findings argue for investing in interoperable, user-centered infrastructures, reimbursing digital workloads, embedding protected-time digital-competency training, and establishing governance standards that ensure equitable, safe, and relationally sustainable digital primary care in routine European general practice. Future research should extend beyond early-adopter countries and short-term evaluations to examine the long-term effects of digital transformation on workload, continuity of care, and health equity in diverse European primary care settings.

## Supplementary material

10.2196/83343 Multimedia Appendix 1 Search strategy, known papers, and AI parameters.

10.2196/83343 Multimedia Appendix 2 Search update 2025.

10.2196/83343 Multimedia Appendix 3 List of excluded studies and exclusion reasons.

10.2196/83343 Multimedia Appendix 4 Thematic coding, extraction template, and detailed extracted data.

10.2196/83343 Multimedia Appendix 5 Comprehensive table of evidence.

10.2196/83343 Multimedia Appendix 6 Mixed Methods Appraisal Tool scores and risk of bias assessment.

10.2196/83343 Multimedia Appendix 7 Full TAM-NPT (technology acceptance model–normalization process theory) breakdown and sensitivity analysis.

10.2196/83343 Checklist 1 PRISMA checklist.

10.2196/83343 Checklist 2 SWiM reporting items checklist.

10.2196/83343 Checklist 3 PRESS 2015 Evidence-Based Checklist.

## Abbreviations

CDSS: clinical decision support system
COM-B: Capability, Opportunity, Motivation – Behavior
DHA: digital health application
DHT: digital health tool
EHR: electronic health record
EMR: electronic medical record
GP: general practitioner
GRADE: Grading of Recommendations, Assessment, Development, and Evaluation
JBI: Joanna Briggs Institute
LTC: long-term condition
MMAT: Mixed Methods Appraisal Tool
NHS: National Health Service
NPT: normalization process theory
OECD: Organisation for Economic Co-Operation and Development
OSF: Open Science Framework
PICO: Population, Intervention, Comparator, and Outcome
PRESS: Peer Review of Electronic Search Strategies
PRISMA: Preferred Reporting Items for Systematic Reviews and Meta-Analyses
TAM: technology acceptance model
VC: video consultation
