# Supplementary material for: The Impact of Digitalization on European General Practice From the Perspective of General Practitioners: Systematic Review
Source: J Med Internet Res. 2026 Aug 18;28:e83343. doi: 10.2196/83343 (PMC13485116; doi:10.2196/83343)
Supplement: Supplementary file 1 [file jmir-v28-e83343-s001.docx]

**Search String, Known papers, AI Parameters**

A consistent query string is adapted to each database's syntax. The core Boolean logic and keywords are: ("digital" OR "digitalization" OR "eHealth" OR "telemedicine" OR "artificial intelligence") AND ("primary care" OR "general practice" OR "general practitioner" OR "family doctor")

**PubMed**

((((general practice[Title]) OR (primary care[Title])) AND (digital*[Title]) AND (2014:2024[pdat]))) AND (("Albania" OR "Andorra" OR "Armenia" OR "Austria" OR "Azerbaijan" OR "Belarus" OR "Belgium" OR "Bosnia and Herzegovina" OR "Bulgaria" OR "Croatia" OR "Cyprus" OR "Czech Republic" OR "Denmark" OR "Estonia" OR "Finland" OR "France" OR "Georgia" OR "Germany" OR "Greece" OR "Hungary" OR "Iceland" OR "Ireland" OR "Italy" OR "Kazakhstan" OR "Kosovo" OR "Latvia" OR "Liechtenstein" OR "Lithuania" OR "Luxembourg" OR "Malta" OR "Moldova" OR "Monaco" OR "Montenegro" OR "Netherlands" OR "North Macedonia" OR "Norway" OR "Poland" OR "Portugal" OR "Romania" OR "Russia" OR "San Marino" OR "Serbia" OR "Slovakia" OR "Slovenia" OR "Spain" OR "Sweden" OR "Switzerland" OR "Turkey" OR "Ukraine" OR "United Kingdom" OR "Vatican City"))

**Wiley**

Keyword search result for [Publication Title: general practice] AND [All: digital] AND [Earliest: (01/01/2014 TO 31/12/2024)]

**Scopus**

TITLE-ABS ("digital health" OR ehealth OR "digital technology" OR "health technology") AND TITLE-ABS ("primary care" OR "general practice" OR "family medicine") AND TITLE-ABS (Europe OR "European Union" OR Albania OR Andorra OR Armenia OR Austria OR Azerbaijan OR Belarus OR Belgium OR "Bosnia and Herzegovina" OR Bulgaria OR Croatia OR Cyprus OR "Czech Republic" OR Denmark OR Estonia OR Finland OR France OR Georgia OR Germany OR Greece OR Hungary OR Iceland OR Ireland OR Italy OR Kazakhstan OR Kosovo OR Latvia OR Liechtenstein OR Lithuania OR Luxembourg OR Malta OR Moldova OR Monaco OR Montenegro OR Netherlands OR "North Macedonia" OR Norway OR Poland OR Portugal OR Romania OR Russia OR "San Marino" OR Serbia OR Slovakia OR Slovenia OR Spain OR Sweden OR Switzerland OR Turkey OR Ukraine OR "United Kingdom" OR "Vatican City") AND PUBYEAR > 2013 AND PUBYEAR < 2025 AND TITLE-ABS (quantitative OR outcomes OR effectiveness OR impact OR evaluation)

**Cochrane**

("digital health" OR eHealth OR "digital technology" OR "health technology") AND ("primary care" OR "general practice" OR "family medicine") AND (Europe OR "European Union" OR Albania OR Andorra OR Armenia OR Austria OR Azerbaijan OR Belarus OR Belgium OR "Bosnia and Herzegovina" OR Bulgaria OR Croatia OR Cyprus OR "Czech Republic" OR Denmark OR Estonia OR Finland OR France OR Georgia OR Germany OR Greece OR Hungary OR Iceland OR Ireland OR Italy OR Kazakhstan OR Kosovo OR Latvia OR Liechtenstein OR Lithuania OR Luxembourg OR Malta OR Moldova OR Monaco OR Montenegro OR Netherlands OR "North Macedonia" OR Norway OR Poland OR Portugal OR Romania OR Russia OR "San Marino" OR Serbia OR Slovakia OR Slovenia OR Spain OR Sweden OR Switzerland OR Turkey OR Ukraine OR "United Kingdom" OR "Vatican City") AND (quantitative OR outcomes OR effectiveness OR impact OR evaluation):ti,ab,kw with Publication Year from 2014 to 2024, with Cochrane Library publication date Between Jan 2014 and Jan 2025, in Trials (Word variations have been searched)

**Web of Science**

("digital health" OR eHealth OR "digital technology" OR "health technology") AND ("primary care" OR "general practice" OR "family medicine") AND (Europe OR "European Union") (All Fields), Index Date 2014-01-01 to 2025-12-31

**Ovid**

("digital health" OR eHealth OR "digital technology" OR "health technology") AND ("primary care" OR "general practice" OR "family medicine") AND (Europe OR "European Union" OR Albania OR Andorra OR Armenia OR Austria OR Azerbaijan OR Belarus OR Belgium OR "Bosnia and Herzegovina" OR Bulgaria OR Croatia OR Cyprus OR "Czech Republic" OR Denmark OR Estonia OR Finland OR France OR Georgia OR Germany OR Greece OR Hungary OR Iceland OR Ireland OR Italy OR Kazakhstan OR Kosovo OR Latvia OR Liechtenstein OR Lithuania OR Luxembourg OR Malta OR Moldova OR Monaco OR Montenegro OR Netherlands OR "North Macedonia" OR Norway OR Poland OR Portugal OR Romania OR Russia OR "San Marino" OR Serbia OR Slovakia OR Slovenia OR Spain OR Sweden OR Switzerland OR Turkey OR Ukraine OR "United Kingdom" OR "Vatican City") AND (quantitative OR outcomes OR effectiveness OR impact OR evaluation) {Including Limited Related Terms}; Index Date 2014-01-01 to 2024-12-31

**SpringerLink**

("digital health" OR eHealth OR "digital technology" OR "health technology") AND ("primary care" OR "general practice" OR "family medicine") AND (Europe OR "European Union") AND (quantitative OR outcomes OR effectiveness OR impact OR evaluation); Index Date 2014-01-01 to 2024-12-31

**Google Scholar**

allintitle: Europe AND "digital health" OR "eHealth" OR "digital technology" OR "health technology" OR "primary care" OR "general practice" OR "family medicine" AND “quantitative” OR “outcomes” OR “effectiveness” OR “impact” OR “evaluation”, Index Date 2014-2025

**Known Papers**

| **Title** | **Author and Year** | **DOI** |
| --- | --- | --- |
| GPs' perspectives on eHealth use in the Czech Republic: a cross-sectional mixed design survey study | Klocek et al., 2019 [31] | 10.3399/bjgpopen19X1  01655 |
| Digital Media for Primary Health Care in Austria | Kriegel et al., 2017 [29] | 10.3233/978-1-61499-759-7-282 |
| Determinants of the implementation of eHealth-based long-term follow-up care for young cancer survivors: a qualitative study | Stamer et al., 2024 [32] | 10.1186/s12885-024-12910-6 |
| Modeling and Predicting Outcomes of eHealth Usage by European Physicians: Multidimensional Approach from a Survey of 9196 General Practitioners | Torrent-Sellens et al., 2018 [30] | 10.2196/jmir.9253 |
| General Practitioners' Attitudes Toward Artificial Intelligence-Enabled Systems: Interview Study | Buck et al., 2022 [33] | 10.2196/28916 |

**Elicit AI** [1]

Elicit AI was used to identify additional literature and help manage search results, suggest potentially relevant records, and semi‑automatically extract bibliographic and study‑level information into structured tables. Elicit AI does not access unpublished data; it operates on search results retrieved from the specified databases and exports structured tables that have been manually checked and corrected by the authors. All screening decisions, corrections, and final data used for synthesis were made and verified by the authors.

Question-String:
How is digitalization impacting general practitioners in European countries?
What are general practitioners' expectations of digitalization in medicine and of AI support in general practice?

**Elicit AI Implementation**

Gray literature in our study refers to relevant papers identified through Elicit AI that did not appear in our systematic database searches (PubMed, Scopus, SpringerLink, Wiley Online Library, Cochrane Library, Web of Science, Google Scholar, and Ovid). These papers met our inclusion criteria but would have been missed using traditional database searches alone.

The validation markers comprised five relevant studies used to validate our search strategy (see the “Known Papers” above).

Gray literature was defined as potentially relevant peer-reviewed, published original study material not retrieved via the eight structured database searches; theses, dissertations, newsletters, blogs, conference proceedings, preprints, and technical/implementation reports were not considered.

**Replicable Elicit AI Workflow**

**Search Process:**

1. **Platform:** Elicit AI Pro version (https://elicit.com/)
2. **Research Questions (entered into the Elicit AI search field):** "How is digitalization impacting general practitioners in European countries?" “What are general practitioners' expectations of digitalization in medicine and of AI support in general practice?”
3. **Search Settings:**
   - Database scope: All available sources in Elicit (not limited to PubMed or clinical trials)
   - Language Filter: English/German
   - Population and phenomenon of interest aligned with the main eligibility criteria (GPs in European primary care, digital health technologies)
   - Date range: 2014-2024
   - Tool and dates: Elicit AI was used via the browser-based web interface; the last Elicit-supported search and export was performed on 5 January 2025, concurrent with the final database update.
4. **Iterative Loading:** Papers were loaded iteratively for each research question (10 at a time) until approximately 40 papers were identified for each research question, stopping when titles no longer appeared relevant. Manually applied: Excluded systematic reviews and general reviews 🡪 selection of 72 papers from Elicit AI.

**Data extraction with Elicit AI**

For data extraction, the predefined Elicit AI parameter list was utilized.

Database search results from PubMed, Scopus, etc., were imported into Elicit; Elicit then generated ranked study lists and structured tables, which were exported as CSV and merged into the main extraction sheet.

**Elicit AI Parameter List**

Column presets

- Summary of discussion
- Main findings
- Summary
- Study design
- Participant count
- Region

Added Columns

- Methodology
- Intervention
- Outcome measured
- Intervention effects
- Summary of introduction
- Summary of discussion
- Study design
- Study objectives
- Theoretical framework
- Research question
- Dataset
- Independent variables
- Dependent variables
- Measured variables
- Statistical techniques
- Population characteristic

**Quality Assurance**

- Initial calibration: Five studies were extracted by both Elicit AI and the first author (JF) independently, achieving 100% overlap.
- JF cross-checked all Elicit AI extractions.
- Discrepancies were resolved using manually extracted data.
- The extraction format included three components for each data point:
  - Elicit AI provided QUOTES (direct text from papers), REASONING (extraction logic), and CONFIDENCE scores for all extracted parameters.

**Limitations Note:** The complete Elicit AI search results require a Pro version license to replicate. The following links to Elicit AI research results and data extraction are available below, but require an appropriate subscription for full access. Note that the link provides only a partial overview of the extracted data, as the data had to be retrieved in multiple requests due to Elicit AI's data-handling capacity at that time.

How is digitalization impacting general practitioners in European countries? https://elicit.com/notebook/c3287992-2b8b-4312-b0e1-051d6c14ba80

What are general practitioners' expectations of digitalization in medicine and of AI support in general practice? https://elicit.com/notebook/008485dd-d3c7-4c35-9490-e21bf337dbec

Data extraction sample: <https://elicit.com/review/ca8f3798-2fc8-4c97-a2ac-e46eddf49d04>

**References**

31. Klocek A, Šmahelová M, Knapová L, Elavsky S. GPs’ perspectives on eHealth use in the Czech Republic: a crosssectional mixed-design survey study. BJGP Open. Oct 2019;3(3):bjgpopen19X101655. [doi: 10.3399/ bjgpopen19X101655] [Medline: 31344683]

29. Kriegel J, Tuttle-Weidinger L, Reckwitz L. Digital Media for Primary Health Care in Austria. Stud Health Technol Inform. 2017;236:282-289. [doi: 10.3233/978-1-61499-759-7-282] [Medline: 28508808]

32. Stamer T, Traulsen P, Rieken J, Schmahl T, Menrath I, Steinhäuser J. Determinants of the implementation of eHealthbased long-term follow-up care for young cancer survivors: a qualitative study. BMC Cancer. Sep 18, 2024;24(1):1159. [doi: 10.1186/s12885-024-12910-6] [Medline: 39294671]

30. Torrent-Sellens J, Díaz-Chao Á, Soler-Ramos I, Saigí-Rubió F. Modeling and predicting outcomes of eHealth usage by European physicians: multidimensional approach from a survey of 9196 general practitioners. J Med Internet Res. Oct 22, 2018;20(10):e279. [doi: 10.2196/jmir.9253] [Medline: 30348628]

33. Buck C, Doctor E, Hennrich J, Jöhnk J, Eymann T. General practitioners’ attitudes toward artificial intelligence-enabled systems: interview study. J Med Internet Res. Jan 27, 2022;24(1):e28916. [doi: 10.2196/28916] [Medline: 35084342]
