# Supplementary material for: The Impact of Digitalization on European General Practice From the Perspective of General Practitioners: Systematic Review
Source: J Med Internet Res. 2026 Aug 18;28:e83343. doi: 10.2196/83343 (PMC13485116; doi:10.2196/83343)
Supplement: Supplementary file 2 [file jmir-v28-e83343-s002.docx]

**Search update (01. January 2025 – 07. October 2025)**

**PRISMA 2020 flow diagram for updated systematic reviews**

**Previous studies**

**Identification of new studies via databases and registers**

Records identified from*:

Pub Med (n = 8)

Scopus (n = 9)

Springer Link (n = 1)
Wiley (n = 0)
Cochrane (n = 19)
Web of Science (n = 7)

Ovid (n = 62)
Google Scholar (n = 29)

Studies included in previous version of review (n = 65)

Records removed *before screening*:

Duplicate records removed (n = 22)

**Identification**

Total studies included in review

(n = 73)

Full-text articles assessed for eligibility

(n = 14)

Abstract screened

(n = 47)

Title screened

(n = 113)

Titles excluded**

(n = 66)

Abstracts excluded

(n = 33)

**Screening**

Reports excluded:

Wrong publication type (n = 2)

Wrong population (n = 3)

Not peer-reviewed (n = 1)

New studies included in review

(n = 8)

**Included**

*Consider, if feasible to do so, reporting the number of records identified from each database or register searched (rather than the total number across all databases/registers).

**If automation tools were used, indicate how many records were excluded by a human and how many were excluded by automation tools.

Source: Page MJ, et al. BMJ 2021;372:n71. doi: 10.1136/bmj.n71.

This work is licensed under CC BY 4.0. To view a copy of this license, visit https://creativecommons.org/licenses/by/4.0/

**Evidence Table**

| **Authors** | **Country** | **Key Findings** |
| --- | --- | --- |
| Bishop et al., 2025 [1] | United Kingdom | - Main outcomes: Practitioners were keen to reflect on and enhance communication skills; willing to undertake digital training (EMPathicO). - Significant observations: Some practitioners would have declined if video-recording consultations was mandatory; practitioners found EMPathicO brief, relevant, and engaging; minor feasibility issues identified including need for incentivizing participation.  -Recommendations: Proceed to full trial of EMPathicO effects on patient outcomes if video-recording is optional, not mandatory; develop and test sophisticated questionnaire structures when planning primary care patient surveys. |
| Ficapal-Cusí et al., 2025 [2] | Spain | - Main outcomes: Moderate technostress levels overall (mean=2.93±0.60) among 587 healthcare professionals (423 physicians, 164 nurses); no significant differences between physicians and nurses; older professionals experienced significantly higher technostress. - Significant observations: Key stressors included techno-overload and techno-uncertainty; familiarity with digital health tools and voluntary usage associated with lower technostress; technical difficulties and lack of perceived usability increased stress levels.  -Recommendations: Implement targeted strategies, including training and technical support, to mitigate stress; promote well-being among healthcare workers; ensure digital health tools are implemented with consideration for age-related differences. |
| Greenhalgh et al., 2025 [3] | United Kingdom | - Main outcomes: Wide variation in digital maturity among UK general practices; practices transitioned from remote-by-default services to hybrid models combining remote and in-person care; large size, slack resources, high absorptive capacity, strong leadership, and good intrapractice relationships favored innovation. - Significant observations: When technologies fitted poorly with tasks and routines or embedding efforts were unsuccessful, inefficiencies and “techno-stress” resulted; continuity of care became harder to achieve; concerns were raised about over-investigation, over-prescribing, and compromises to preventive medicine; widening disparities of access between advantaged and disadvantaged patients.  -Recommendations: Offer tailored support based on digital maturity typology; explore strategically traditional practices in deprived settings; consider local strategic choices when assessing low use of digital technologies. |
| Greenhalgh et al., 2025 [4] | United Kingdom | - Main outcomes: UK general practices settled into a hybrid model combining remote/digital and in-person care, with wide variation in digital maturity across 12 practices. Remote and digital services brought both efficiency gains for some tasks and significant complexity and workload for others. - Significant observations: Digitally enabled patients often found remote access convenient, whereas multiply disadvantaged groups experienced digital exclusion and reduced access. Remote pathways sometimes increased workload, generated techno‑stress, fragmented continuity of care, and widened inequities, but these effects were not uniform across practices.  -Recommendations: Support practices according to their digital maturity, distinguishing warranted “strategically traditional” models from those needing resources and training. National and regional bodies should address safety, equity, continuity, training, infrastructure, and patient support when scaling remote and digital services. |
| Jendly et al., 2025 [5] | OECD countries (Canada, France, Germany, Netherlands, Sweden, UK, Switzerland, New Zealand, Australia, Japan) | - Main outcomes: Across 10 OECD countries, digital health use was high but varied, with the Netherlands and UK showing among the highest digital health scores and Germany and Switzerland among the lowest. Approximately one‑third of physicians reported burnout overall; however, digital health use was not associated with burnout, stress, or work–life balance in adjusted analyses. - Significant observations: In European countries (France, Germany, Netherlands, Sweden, UK, Switzerland), burnout prevalence and digital health scores varied substantially, yet higher digital use did not predict higher burnout. Higher digital health scores, however, were associated with greater dissatisfaction with daily workload, suggesting that digital tools may increase perceived workload or be adopted in already high‑pressure settings.  -Recommendations: Policymakers should not assume digital health is a primary driver of physician burnout but should ensure that digital tools are designed and implemented to reduce administrative burden and workload. Further research is needed on how to optimize digital health so that it alleviates, rather than exacerbates, workload dissatisfaction among primary care physicians. |
| Sanavro et al., 2025 [6] | Netherlands | - Main outcomes: 4.4% estimated increase in proportion of appropriate referrals among general practitioner (GPs) practices with access to the Prisma digital consultation platform (OR 1.22, 95% CI 1.01-1.46, P=0.037); per-protocol analysis showed smaller 2.2% difference (non-significant). - Significant observations: Platform had modest positive effect on appropriateness of referrals for orthopaedic review; effect was consistent until 39 weeks of exposure then attenuated; surgical yield was 10.5% in control vs 13.1% in intervention condition.  -Recommendations: On a larger scale, the platform could contribute to more sustainable access to specialist care; conduct qualitative research to understand how perceived knowledge and skills relate to platform use; consider using platform for training purposes. |
| Schäfer et al., 2025 [7] | Germany | - Main outcomes: Focus groups with 32 GPs and 33 older patients showed that the gp‑multitool.de web application, based on the German multimorbidity guideline, is generally usable and relevant for shared prioritisation of treatments, structured assessments, and medication review. Iterative feedback led to substantial refinements of assessments, wording, scales, and functions to better fit routine primary care and patient capabilities. - Significant observations: GPs wished to avoid time-consuming or clinically unresolvable items, while patients highlighted clarity, comprehensibility, and not being overtaxed cognitively. Some GPs were concerned about data security, whereas most patients were unconcerned and willing to share data; both groups identified minor usability issues and device-related glitches.  -Recommendations: Use focus groups and iterative co-design to tailor digital guideline tools to end-user needs, simplifying assessments and interfaces, and aligning content with problems GPs can actually address. Pilot and evaluate such tools in everyday care, and address technical integration (e.g., with practice systems) and support for older or less digitally skilled patients. |
| Würtenberger et al., 2025 [8] | Germany | - Main outcomes: Medium levels of technostress perception among 114 German GPs; substantial levels of technostress inhibitors present; GPs reported occasional burnout symptoms, moderate job satisfaction, and generally good health status. - Significant observations: Using ICT leads to heightened stress, increased burnout symptoms, and reduced job satisfaction; technostress creators include techno-overload, techno-complexity, techno-uncertainty, techno-invasion, and techno-insecurity; literacy facilitation and technical support can mitigate negative psychological effects.  -Recommendations: Implement preventive strategies to mitigate stress and improve well-being; provide technical support and literacy facilitation; enhance employee engagement in understanding digital technologies. |

1.	Bishop F, Howick J, Vennik J, et al. Feasibility trial of a new digital training package to enhance primary care practitioners’ communication of clinical empathy and realistic optimism. Bongelli R, ed. *PLOS One*. 2025;20(7):e0324649. doi:10.1371/journal.pone.0324649. PMID:40679977

2.	Ficapal-Cusí P, Torrent-Sellens J, Yuguero O. Technostress among healthcare professionals in Spain: a cross-sectional survey on the impact of digital health tools. *BMJ Open*. 2025;15(9):e103783. doi:10.1136/bmjopen-2025-103783. ISSN:2044-6055, 2044-6055

3.	Greenhalgh T, Clarke A, Byng R, et al. After the disruptive innovation: how remote and digital services were embedded, blended and abandoned in UK general practice – longitudinal study. *Health Soc Care Deliv Res*. Published online June 2025:1-37. doi:10.3310/KRWS4334. ISSN:2755-0079

4.	Greenhalgh T, Alvarez Nishio A, Clarke A, et al. Remote and digital services in UK general practice 2021–2023: the remote by default 2 longitudinal qualitative study synopsis. *Health Soc Care Deliv Res*. 2025;(31):1-49. doi:10.3310/QQTT4411. ISSN:2755-0079

5.	Jendly M, Santschi V, Tancredi S, Von Wyl V, Chiolero A. Primary care physician digital health profile and burnout: an international cross-sectional study. *Eur J Public Health*. 2025;35(6):1162-1169. doi:10.1093/eurpub/ckaf106. ISSN:1101-1262, 1464-360X

6.	Sanavro SM, Van Der Worp H, Schers H, et al. Impact of an interdisciplinary digital consultation platform on general practitioner referrals for musculoskeletal symptoms: a stepped wedge cluster randomized trial. *Fam Pract*. 2025;42(5):cmaf071. doi:10.1093/fampra/cmaf071. ISSN:0263-2136, 1460-2229

7.	Schäfer I, Menzel A, Lezius S, et al. Effects of a digital tool implementing the german S3 clinical practice guideline for multimorbidity in primary care (gp-multitool.de): study protocol for a cluster-randomised controlled trial. *BMJ Open*. 2025;15(8):e102086. doi:10.1136/bmjopen-2025-102086. PMID:40754332

8.	Würtenberger A, Groneberg DA, Mache S. Digital stress perception and associations with work- and health-related outcomes among general practitioners in Germany: a quantitative study. *BMC Health Serv Res*. 2025;25(1):535-548. doi:10.1186/s12913-025-12653-5. ISSN:1472-6963

**Abbreviations**

GP: General practitioner

ICT: Information and communication technology

OECD: Organisation for Economic Co-operation and Development

PRISMA: Preferred Reporting Items for Systematic Reviews and Meta-Analyses

UK: United Kingdom
