# Supplementary material for: The Impact of Digitalization on European General Practice From the Perspective of General Practitioners: Systematic Review
Source: J Med Internet Res. 2026 Aug 18;28:e83343. doi: 10.2196/83343 (PMC13485116; doi:10.2196/83343)
Supplement: Supplementary file 3 [file jmir-v28-e83343-s003.docx]

**Exclusion Studies**

| **No.** | **Author/Year** | **Title** | **Reason Code** |
| --- | --- | --- | --- |
| 1 | Abel et al., 2024 | Current experience and future potential of facilitating access to digital NHS primary care services in England: the di-facto mixed-methods study | 1 |
| 2 | Ardielli, 2021 | Implementation of eHealth applications by primary care physicians in the european union member states | 1 |
| 3 | Ardielli, 2021 | Use of eHealth tools by primary care physicians according to the provided healthcare in the Czech Republic | 1, 3 |
| 4 | Assing Hvidt et al., 2023 | Low adoption of video consultations in post–COVID-19 general practice in northern Europe: barriers to use and potential action points | 2 |
| 5 | Boers et al., 2020 | SERIES: eHealth in primary care. Part 2: exploring the ethical implications of its application in primary care practice | 1, 2 |
| 6 | Botan et al., 2023 | Examiner perceptions of the MRCGP recorded consultation assessment for general practice licensing during COVID-19: cross-sectional study | 1 |
| 7 | Brennan et al., 2015 | National health models and the adoption of E-health and E-prescribing in primary care – new evidence from Europe | 1 |
| 8 | Brünn et al., 2022 | Use of an electronic medication management support system in patients with polypharmacy in general practice: a quantitative process evaluation of the AdAM trial | 1 |
| 9 | Clausen et al., 2023 | Digital solutions for decision support in general practice – a rapid review focused on systems developed for the universal healthcare setting in Denmark | 1 |
| 10 | Cooper and Heath, 2024 | The digital front door: demise of the ‘therapeutic history’? | 4, 1, 2 |
| 11 | Dakin et al., 2024 | Access and triage in contemporary general practice: a novel theory of digital candidacy | 1, 3 |
| 12 | De Rosis and Seghieri, 2015 | Basic ICT adoption and use by general practitioners: an analysis of primary care systems in 31 european countries | 1 |
| 13 | Egea et al., 2014 | E-health services adoption by european general practitioners | 6 |
| 14 | Glock et al., 2024 | eVisits to primary care and subsequent health care contacts: a register-based study | 1, 3 |
| 15 | Gomez-Cabello et al., 2024 | Artificial-intelligence-based clinical decision support systems in primary care: a scoping review of current clinical implementations | 1, 2 |
| 16 | Gottliebsen and Petersson, 2020 | Limited evidence of benefits of patient operated intelligent primary care triage tools: findings of a literature review | 1 |
| 17 | Harris et al., 2020 | Self-management support activities in primary care: a qualitative study to compare provision across common health problems | 1 |
| 18 | Heyer, 2024 | This is how digital helpers relieve the burden on you and your practice team. A general practitioner reports | 2 |
| 19 |  | Digital medicine and e-health in primary care. Selected abstracts from the virtual 90th EGPRN conference, 16–17 october 2020 | 4 |
| 20 | Jepsen et al., 2022 | Healthcare in the hand: patients’ use of handheld technology in video consultations with their general practitioner | 1, 2 |
| 21 | Kasteleyn et al., 2021 | SERIES: eHealth in primary care. Part 5: a critical appraisal of five widely used eHealth applications for primary care – opportunities and challenges | 2 |
| 22 | Keuper et al., 2024 | The impact of eHealth use on general practice workload in the pre-COVID-19 era: a systematic review | 3, 2 |
| 23 | Kremers, 2024 | eHealth questionnaire and chat tools in the general practice a research on the use of digital tools in dutch general practices and its impacts on healthcare professionals | 2 |
| 24 | Ladds et al., 2023 | The impact of remote care approaches on continuity in primary care: a mixed-studies systematic review | 1, 3 |
| 25 | Li et al., 2022 | General practitioners’ perceptions of using virtual primary care during the COVID-19 pandemic: an international cross-sectional survey study | 3 |
| 26 | Lüchau et al., 2024 | The invisible work of video consultation use in danish general practice: an ethnographic study | 1 |
| 27 | Majekodunmi, 2021 | Artificial intelligence: current and future impact on general practice | 2 |
| 28 | Mariotti et al., 2022 | Consensus among clinicians on referrals’ priority and use of digital decision-making support systems | 1 |
| 29 | McMillan et al., 2022 | Implications of the changes to patient online records access in english primary care | 2 |
| 30 | Mold et al., 2019 | Electronic consultation in primary care between providers and patients: systematic review | 5 |
| 31 | Morrow et al., 2017 | Exploring the perspectives of clinical professionals and support staff on implementing supported self-management for asthma in UK general practice: an IMP2ART qualitative study | 1 |
| 32 | Nazir et al., 2023 | Digital transformation at hall green health – a model for modern general practice | 1, 3 |
| 33 | Neve et al., 2020 | Digital health in primary care: risks and recommendations | 3 |
| 34 | Nguyen et al., 2024 | Communication during telemedicine consultations in general practice: perspectives from general practitioners and their patients | 3 |
| 35 | Pagliari, 2021 | Digitally-enabled primary care: past, present and prospects | 1 |
| 36 | Painter et al., 2023 | Confidence in AI in general practice | 2 |
| 37 | Payne et al., 2024 | What are the challenges to quality in modern, hybrid general practice? A multi-site longitudinal study | 3 |
| 38 | Piera-Jiménez et al., 2024 | Strengthening primary health care in Europe with digital solutions | 1 |
| 39 | Radionova et al., 2023 | Impacts of symptom checkers for laypersons’ self-diagnosis on physicians in primary care: scoping review | 2 |
| 40 | Salisbury et al., 2020 | The impact of digital-first consultations on workload in general practice: modeling study | 2 |
| 41 | Söling et al., 2020 | From sensitization to adoption? A qualitative study of the implementation of a digitally supported intervention for clinical decision making in polypharmacy | 1 |
| 42 | Sørensen et al., 2023 | Machine learning in general practice: scoping review of administrative task support and automation | 5 |
| 43 | Summerton and Candsdale, 2019 | Artificial intelligence and diagnosis in general practice | 2 |
| 44 | Tabaeeian et al., 2024 | A systematic review of telemedicine systems use barriers: primary health care providers' perspective | 5 |
| 45 | Teixeira et al., 2023 | Digital maturity and its determinants in general practice: a cross-sectional study in 20 countries | 3 |
| 46 | Walley et al., 2024 | Use of telemedicine in general practice in Europe since the COVID-19 pandemic: a scoping review of patient and practitioner perspectives | 2 |
| 47 | Wanderås et al., 2023 | Video consultation in general practice: a scoping review on use, experiences, and clinical decisions | 1, 2, 3 |
| 48 | Wangler and Jansky, 2022 | Welche potenziale und mehrwerte bieten DiGA für die hausärztliche versorgung? – ergebnisse einer befragung von hausärzt*innen in deutschland | 2 |
| 49 | Weik et al., 2024 | Big 5 personality traits and individual- and practice-related characteristics as influencing factors of digital maturity in general practices: quantitative web-based survey study | 1 |
| 50 | White et al., 2024 | Tailoring communication practices to support effective delivery of telehealth in general practice | 2 |
| 51 | Willemsen et al., 2022 | A digital platform to support communication and organization in the general practice: evaluation of healthcare usage and costs using claims data of a health insurer | 1 |
| 52 | Willemsen, 2024 | Patient and healthcare professional perspectives on a digital first general practice: an evaluation study | 2 |
| 53 | Ziebland et al., 2021 | Power, paradox and pessimism: on the unintended consequences of digital health technologies in primary care | 2 |

**Reasons**

1. The paper doesn’t concern the perspectives of GPs.
2. Wrong publication type: The paper is not primary research, or is already included in a systematic review/ meta-analysis, or an economic study already included in this review.
3. Wrong population: The paper does not mainly focus on GPs.
4. There was no full-text available (e.g., only an abstract or preprint).
5. The study doesn’t focus on Europe.
6. The study data is too old or was conducted in a year < 2014.

| **Excluded due to language exclusion criteria/Database (before screening)** | | |
| --- | --- | --- |
| **Database** | **Excluded studies** | **Languages** |
| PubMed | 0 |  |
| Wiley | 0 |  |
| Scopus | 1 | 1xSpanish |
| Cochrane | 0 |  |
| Web of Science | 2 | 2xFrench |
| Ovid | 0 |  |
| Springer Link | 3 | 1xItalian, 1xDutch, 1xFrench |
| Google Scholar | 0 |  |

**Abbreviations**

AI: Artificial intelligence

EGPRN: European General Practice Research Network

GP: General practitioner

ICT: Information and communication technology

MRCGP: Membership of the Royal College of General Practitioners

NHS: National Health Service

UK: United Kingdom
