# Supplementary material for: The Impact of Digitalization on European General Practice From the Perspective of General Practitioners: Systematic Review
Source: J Med Internet Res. 2026 Aug 18;28:e83343. doi: 10.2196/83343 (PMC13485116; doi:10.2196/83343)
Supplement: Supplementary file 4 [file jmir-v28-e83343-s005.docx]

**Evidence Table**

| **Authors** | **Country** | **Key Findings** |
| --- | --- | --- |
| **Artificial Intelligence** | | |
| Blease et al., 2019 [33] | United Kingdom | - Main outcomes: GPs are generally skeptical about artificial intelligence (AI) replacing core functions in general practice, especially in areas that require empathy and communication. - Significant observations: AI is perceived as potentially beneficial for reducing administrative burdens; however, concerns about general practitioners’ (GPs) burnout and workload are prevalent. Recommendations: Further qualitative research is needed on the impact of AI across medical specialties, with consideration given to patient perspectives. |
| Buck et al., 2022 [84] | Germany | - Main outcomes: Identification of five categories influencing GPs' attitudes toward AI-enabled systems: concerns, expectations, environmental influences, individual characteristics, and minimum requirements. - Significant observations: Concerns about AI replacing human roles; expectations of improved diagnostic quality; importance of time efficiency and diagnostic quality as minimum requirements. - Recommendations: Improve AI literacy among GPs; design user-centered AI systems that meet GPs' minimum requirements; focus on promoting positive attitudes toward AI systems. |
| Tabla et al., 2022 [85] | France | - Main outcomes: GPs desire AI modules that assist in urgent diagnosis, integrate with electronic health records (EHRs), save time, and personalize care. - Significant observations: GPs value interoperability and task delegation but are wary of automatic actions without their input. - Recommendations: AI development should prioritize user needs and practical workflow integration. |
| Mahlknecht et al., 2023 [86] | Italy | - Main outcomes: Positive reception among patients; mixed satisfaction among GPs; no significant impact on visit duration. - Significant observations: Concerns about clinical quality and reliability; necessity of physical exams; potential for improved usability and diagnostic accuracy. - Recommendations: Further development needed; explore role in self-management and reducing unnecessary visits; require clinical guidance for integration. |
| Schütze et al., 2023 [87] | Germany | - Main outcomes: Development of an AI-based clinical decision support (CDSS) tailored for GPs to address diagnostic uncertainty. - Significant observations: Identification of user requirements: data entry, result review, communication with colleagues, integration with electronic records. - Recommendations: The System should focus on common diseases with atypical symptoms, not just rare diseases. Importance of integrating CDSS with existing systems to avoid workflow disruption. Involvement of GPs in the design process to ensure the system meets needs and is feasible. |
| Wewetzer et al., 2023 [88] | Germany | - Main outcomes: Financial concerns are the primary barrier to implementing AI-based screening tools in general practice. Technical factors, such as device practicability and integration, are crucial for successful adoption. - Significant observations: Younger physicians are more open to using AI-assisted devices. The general attitude towards AI is moderately positive, but financial concerns remain a significant barrier to its widespread adoption. - Recommendations: Address financial barriers, ensure easy integration into existing systems, and consider interventions adapted to the needs of physicians and patients. |
| Blease et al., 2024 [89] | United Kingdom | - Main outcomes: 20% of GPs use generative AI in practice, primarily for documentation and differential diagnosis. - Significant observations: AI is valued for administrative tasks and clinical reasoning, but lacks guidance and clear policies. - Recommendations: Educate physicians on the benefits and risks of AI; conduct further research on the safe integration of AI. |
| **Digital Health Technologies** | | |
| Peeters et al., 2016 [34] | Netherlands | - Main outcomes: A significant number of GPs offer basic eHealth services, but face barriers such as technical issues and a lack of financial compensation. - Significant observations: GPs recognize the potential benefits of eHealth but are concerned about efficiency and control. There is a gap between the availability of eHealth services and patient awareness. - Recommendations: Increase awareness among patients and GPs, use successful models from other countries, and have GPs promote eHealth services. |
| Albrecht et al., 2017 [29] | Germany | - Main outcomes: High ownership of mobile devices among GPs and patients; significant use by GPs in professional contexts; limited patient-GP interaction via apps. - Significant observations: Age and education influence patient use; concerns about data safety and trustworthiness; a digital divide based on sociodemographic factors. - Recommendations: Enhance compatibility between apps and GP systems, address data protection issues, and improve patient-GP communication regarding the use of apps. |
| Kriegel et al., 2017 [35] | Austria | - Main outcomes: Digital media are used conservatively in primary care, primarily for documentation and communication. There is potential for increased digital networking with health professionals. - Significant observations: Current use includes medical information systems and email communication. Challenges include legal issues and a lack of coordination. - Recommendations: Focus on user requirements and functionalities that add value. Digital media should support coordination and communication in patient care. |
| Cowie et al., 2018 [36] | Scotland | - Main outcomes: eConsult provided an alternative method for accessing GP services, but did not fully meet expectations for promoting self-help. - Significant observations: The presence of a 'champion' facilitates successful integration; barriers include lack of engagement and insufficient support. - Recommendations: Ensure staff engagement, strategic implementation, address marketing alignment, and system integration challenges. |
| Gabriels and Moerenhout, 2018 [37] | Belgium | - Main outcomes: Digital self-tracking is emerging but not yet standard in general practice. It offers potential for patient empowerment and self-management. - Significant observations: Concerns exist about patients' ability to interpret data and potential anxiety. There is a gap between data collection and behavioral change. - Recommendations: Physician training in data interpretation and improving patient digital literacy. |
| Morrissey et al., 2018 [30] | Ireland | - Main outcomes: GPs see potential benefits in digital health technologies (DHTs) for more accurate data and patient engagement, but are concerned about increased workload and responsibility. - Significant observations: Public engagement with technology is high, especially among younger generations. GPs need to be informed about apps before recommending them. Concerns about data management and healthcare use are prevalent among the 'worried well'. - Recommendations: Address workload concerns, ensure data quality, and provide clear guidelines for app use. |
| Torrent-Sellens et al., 2018 [38] | Europe | - Main outcomes: Younger and female GPs, who have positive attitudes towards information and communication technology (ICT), self-employment, and stable or increasing patient numbers, are associated with favorable eHealth outcomes. - Significant observations: The ease of use and flexibility of eHealth systems are crucial for adoption; demographic factors, such as age and gender, influence eHealth usage. Recommendations: Develop specific policies for various contexts and conduct further research on eHealth usage across healthcare systems. |
| Breedvelt et al., 2019 [39] | United Kingdom | - Main outcomes: 72% of GPs use e-mental health interventions; guided interventions are preferred but mostly offered unguided. - Significant observations: Lack of training (91.5% untrained), significant barriers (infrastructure, privacy, awareness). - Recommendations: Increase training for GPs, address implementation barriers, and focus on guided interventions. |
| Buhtz et al., 2019 [40] | Germany | - Main outcomes: High receptiveness among GPs towards DHTs, but low knowledge and training. - Significant observations: Concerns about technology replacing human interaction; other parties influencing technology use more than GPs. - Recommendations: Increase training and support for GPs to enhance their ability to integrate DHTs into practice. |
| Fagerlund et al., 2019 [31] | Norway | - Main outcomes: Digital health services are growing in use; GPs are generally positive about them; benefits include reduced phone load and increased efficiency. - Significant observations: Text-based e-consultations are still limited; benefits for patients include increased flexibility and autonomy; however, concerns exist for certain groups who require traditional alternatives. - Recommendations: More standardized routines and evidence of effectiveness are needed for large-scale adoption. |
| Klocek et al., 2019 [41] | Czech Republic | - Main outcomes: ICT usage in general practice is predicted by eHealth readiness, age, and practice size. - Significant observations: Barriers to mHealth include lack of time, technical support, patient interest, and GP prejudice. Younger GPs and those with larger practices are more likely to use ICT. - Recommendations: System-level changes and educational programs are needed to enhance eHealth adoption. Focus on improving GP understanding of eHealth benefits for patient interaction and care quality. |
| Entezarjou et al., 2020 [42] | Sweden | - Main outcomes: Digital communication is perceived as applicable after adjustment, especially in the context of continuity of care; asynchronous chat facilitates clinical decision-making. - Significant observations: Ambivalence towards digital tools; challenges include information overload and loss of communication nuances, making them unsuitable for all queries. - Recommendations: Explore specific clinical contexts for effective digital communication. |
| Poss-Doering et al., 2020 [43] | Germany | - Main outcomes: Digital tools were supportive for health literacy but faced challenges in integration due to low technology affinity and compatibility issues with existing routines. - Significant observations: Limited uptake of digital interventions; concerns about hygiene and appropriateness for older patients; unanticipated barriers like time constraints. - Recommendations: Address barriers in detail, develop tailored interventions, and consider professional values and patient demographics. |
| Glock et al., 2021 [44] | Sweden | - Main outcomes: Positive attitudes towards telemedicine, but low usage due to barriers such as loss of personal contact and inadequate technology. - Significant observations: Concerns about patient safety, increased workload, and lack of added value from telemedicine. - Recommendations: Evaluate telemedicine tools for clinical usefulness, patient safety, and workload impact; involve end users in evaluations; retain benefits of personal contact. |
| Jakobsen et al., 2021 [45] | Denmark | - Main outcomes: Digital lifestyle coaching is feasible if GPs and primary care nurses understand their roles and the intervention content. Positive attitudes are more likely to exist when interventions are easy to use and provide clear feedback. - Significant observations: Access to real-life data helps set realistic goals. Integration into existing systems is crucial. Lack of digital culture makes eHealth interventions seem inconvenient. - Recommendations: Ensure flexibility, ease of use, integration into EHRs, and support collaboration among healthcare providers. |
| Keuper et al., 2021 [46] | Netherlands | - Main outcomes: Significant increase in e-health usage during COVID-19, especially video consultations (VCs); 75% of practices started or intensified e-health use. - Significant observations: Only a minority intend to continue this usage post-pandemic; group practices used e-health more than solo or duo practices. Recommendations: Address barriers to sustained e-health use and explore factors that encourage continued adoption. |
| Knop et al., 2021 [47] | Germany | - Main outcomes: Digital mediation of delegation processes is complex but promising for improving primary care. Medical assistants (MAs) play a crucial role in facilitating the adoption of digital technologies. Digital technologies, such as VCs and telemedical devices, can enhance patient-centered care and improve efficiency. - Significant observations: The integration of digital technologies can improve healthcare quality, safety, and patient satisfaction. Challenges include ensuring the accuracy of technology and addressing biases in the adoption of technology. - Recommendations: Recommendations include developing compatible technologies and equipping MAs with the necessary skills. |
| Pikkemaat et al., 2021 [48] | Sweden | - Main outcomes: Low actual use but high intention to use telemedicine among Swedish primary care physicians; significant predictors of intention include attitudes and perceived behavioral control. - Significant observations: Perceived behavioral control is the strongest predictor; social norms are weak predictors; low response rate is a limitation. - Recommendations: Focus on empowering physicians' self-efficacy to increase adoption; further research is needed post-COVID-19 to assess changes in intentions. |
| Bondaronek et al., 2022 [49] | United Kingdom | - Main outcomes: Identification of barriers and facilitators to using digital tools in primary care for delivering physical activity advice. - Significant observations: Mixed views on usability and utility; importance of skills, training, efficiency, integration, and simplicity. - Recommendations: Integrate digital tools into existing workflows to ensure simplicity and efficiency, and provide technical support and training. |
| Hammerton et al., 2022 [50] | United Kingdom | - Main outcomes: Significant variation in digital readiness across technologies; electronic patient records have the highest competency scores. - Significant observations: Younger staff and those with less tenure are more digitally competent. Rurality, smaller practice size, and older patient populations are linked to lower readiness. Barriers include IT infrastructure and information governance issues. - Recommendations: Use dedicated digital implementation teams; enhance collaboration among stakeholders; allocate more resources for training and support; address IT infrastructure and usability issues. |
| Van De Vijver et al., 2022 [51] | Netherlands | - Main outcomes: The recommendation rate for the platform increased significantly from 35% among non-users to 76.3% among active users.  - Significant observations: The platform effectively reduced provider workload, improved care quality and accessibility, and supported patient empowerment through the use of self-management tools. Overall usability was high, although some users reported technical issues and a reduction in human interaction.  Recommendations: Enhance human contact features and address technical shortcomings to improve the user experience. Future evaluations should include populations with lower digital and health literacy to ensure broader accessibility and impact. |
| Butler et al., 2023 [52] | United Kingdom | - Main outcomes: Digital interventions, such as SuMMiT-D, have the potential to enhance diabetes care by addressing unmet needs. Compatibility with existing systems, ease of use, and incentivization are crucial for implementation. - Significant observations: Interventions should align with general practice priorities, including holistic care and cultural relevance. Technology can help patients manage information overload. Concerns exist about focusing solely on medication adherence without broader diabetes management. - Recommendations: Resource availability and external support mechanisms are necessary for successful implementation. Simplifying healthcare staff roles in the intervention is important to reduce the burden. Addressing language and cultural barriers is essential. Incentivization is key for sustaining the intervention. Seamless integration into existing routines is needed. |
| Kujansivu et al., 2023 [53] | Finland | - Main outcomes: Digital portals and calculators are used daily; telephones are preferred for remote consultations. - Significant observations: GPs feel competent in information security; there is a positive attitude towards digitalization, but limited use of new tools. - Recommendations for future implementation: Increase eHealth training; involve GPs in tool development to ensure practical integration. |
| Solberg Carlsson et al., 2023 [54] | Sweden | - Main outcomes: Rapid adoption of DHTs was facilitated by decentralized management and local decision-making. Positive experiences with digital systems were reported, with intentions to continue using them. - Significant observations: Balancing standardization with flexibility is crucial for effective implementation. The NASSS framework facilitates the analysis of digital technology implementation. - Recommendations: Recommendations include learning from pandemic experiences and standardizing digital care pathways while maintaining flexibility. |
| Wangler and Jansky, 2023 [55] | Germany | - Main outcomes: GPs see DHAs as valuable for increasing patient motivation and compliance; however, there is limited willingness to use them extensively. - Significant observations: DHAs are perceived as safer and more reliable than conventional health apps; there is a need for better usability, gamification, and training. - Recommendations: Include DHAs in evidence-based guidelines; provide more training programs and reliable information sources; improve usability and gamification features. |
| Brecher et al., 2024 [56] | Germany | - Main outcomes: 59.4% of family doctors (FDs) have prescribed digital health applications (DiGA); 61.1% have a positive attitude towards them; 40.6% have never prescribed DiGA. - Significant observations: Younger and male FDs, as well as those in urban areas, exhibit higher digital affinity and a greater willingness to use DiGA. Patient-related factors, such as digital affinity and health literacy, influence prescribing behavior. - Recommendations: Increase FDs' digital affinity through targeted education and reliable information sources; consider patient-related factors in prescribing decisions. |
| Gavin et al., 2024 [57] | United Kingdom | - Main outcomes: Digital tools for long-term conditions (LTCs) are not well implemented; lack of condition-specificity and usability are significant barriers. - Significant observations: COVID-19 has improved the perception of digital tools; usability, acceptability, and evidence base are crucial for adoption. Recommendations: Ensure digital tools are evidence-based, integrated into existing IT systems, and supported by professional networks and the voluntary sector. |
| Ghis Malfilatre and Louvel, 2024 [58] | France | - Main outcomes: Digital tools are introduced to enhance GPs' capacity and recognition; adoption depends on alignment with professional culture. - Significant observations: Three phases of digitalization identified; empowerment through digital tools; varied adoption based on cultural alignment. - Recommendations: Study the impact of digital tools on professional culture and address normative tensions in technology adoption. |
| Nassehi and Ramvi, 2024 [59] | Norway | - Main outcomes: DHTs, such as Secure Digital Messaging and EHRs, enhance efficiency and patient interaction management, but challenge personal engagement. - Significant observations: GPs emphasize the importance of integrating digital tools to support relational and ethical caregiving. Digital tools increase accessibility but can delay treatment if not promptly addressed. Patients may overly rely on data from consumer devices. - Recommendations: Ongoing evaluation of digital tools is necessary to ensure they align with ethical care practices. The challenge is to maintain the quality of GP-patient relationships while using digital tools. |
| Newbould et al., 2024 [60] | United Kingdom | - Main outcomes: Digital First Primary Care can improve access and speed of consultations, but may compromise continuity of care and clinician-patient relationships. - Significant observations: Clinicians prefer face-to-face interactions for complex cases; however, concerns exist about the increased workload and patient literacy challenges. - Recommendations: Digital First Primary Care should complement face-to-face consultations; further research is needed to incorporate patient perspectives and comprehensively evaluate the impacts. |
| Posselt et al., 2024 [61] | Germany | - Main outcomes: DiGAs are frequently prescribed by GPs, especially for mental health conditions like depression. - Significant observations: There is a discrepancy between GPs' and patients' perspectives on the use of digital therapeutics, with GPs viewing them as educational tools. Patients prefer to receive information about digital therapeutics from healthcare professionals. Strengthening digital health literacy is crucial and should be addressed during consultations. Health professionals often misjudge patients' digital health literacy, which can hinder effective DiGA use. Recommendations: GPs require additional time to engage in necessary training for DiGA implementation. |
| Potthoff et al., 2024 [62] | Norway | - Main outcomes: The digital alcohol intervention "Endre" was not fully embedded in practice due to low normalization scores and several barriers. - Significant observations: Barriers included a lack of tailored support, passive staff involvement, diminished trust, and the absence of feedback loops. The COVID-19 pandemic also limited implementation. - Recommendations: Involve support staff in the development and implementation process, enhance feedback systems, and integrate digital solutions with features such as dashboards for monitoring patient progress. |
| Preiser et al., 2024 [63] | Germany | - Main outcomes: GPs are open to DHTs but have limited experience with symptom checkers. They see potential benefits in reducing administrative tasks, but also potential drawbacks if these tools increase workload or patient anxiety. - Significant observations: Symptom checkers are not yet widely used in German general practice. GPs view them as supplementary tools rather than replacements for professional diagnosis. - Recommendations: Ensure symptom checkers are integrated in ways that reduce workload and enhance patient care without disrupting the doctor-patient relationship. |
| Schendzielorz et al., 2024 [64] | Germany | - Main outcomes: Emphasis on data protection and maintaining physician-patient relationships; increased digitalization in communication and documentation. - Significant observations: Concerns about digital technologies replacing core medical activities; need for digital competencies in medical education. - Recommendations: Integrate digital competencies into medical training; ensure digital technologies complement core medical activities. |
| Wangler and Jansky, 2024 [65] | Germany | - Main outcomes: GPs generally view DHAs positively, noting benefits in patient compliance, mobility, and education. However, actual prescription rates are low. - Significant observations: There is a lack of confidence among GPs in advising on DHAs due to insufficient training and information. Usability improvements and structured training are needed. - Recommendations: Enhance usability, provide comprehensive training, improve information sources, and address legal and practical concerns to integrate DHAs into primary care better. |
| Weik et al., 2024 [66] | Germany | - Main outcomes: Moderate barriers to digital health adoption identified; significant predictors include practice-related characteristics and digital maturity. - Significant observations: GPs wish for improved interoperability, technical support, and usability; insufficient technical infrastructure is a minor barrier. Recommendations: Enhance design compatibility with existing workflows, provide ongoing training, and reconsider reimbursement schemes. |
| **Medical records** | | |
| Cabitza et al., 2014 [90] | Italy | - Main outcomes: High adoption of electronic medical records (EMRs) among GPs (98%). Positive Net Promoter Score (+13%) indicates general satisfaction but highlights areas for improvement. - Significant observations: Satisfaction with advanced features (mobility, data sharing) but dissatisfaction with core functionalities. EMRs improve documentation and the appropriateness of work, but they also increase stress levels, and there is a preference for increasing email communication with patients over phone or social media. - Recommendations: Focus on improving core functionalities and prioritizing features such as data importing and backup. |
| Blease et al., 2023 [91] | United Kingdom | - Main outcomes: GPs expressed concerns about increased workload, patient anxiety, changes in documentation practices, and legal risks associated with online record access (ORA). - Significant observations: GPs anticipate ORA will lead to extra work and reduced efficiency. Concerns about patient safety and increased litigation were also noted. - Recommendations: Conduct ongoing qualitative research post-implementation to assess actual impacts and address GPs' concerns. |
| Davidge et al., 2023 [92] | United Kingdom | - Main outcomes: Recognition of ORA's potential to improve patient engagement and health literacy; concerns about data protection, patient safety, and increased workload. - Significant observations: Potential cultural shift in primary care; changes in staff-patient relationships; concerns about vulnerable patient safety. - Recommendations: The need for staff training, support, and resources to address staff concerns regarding the safe and equitable implementation of ORA. |
| Turner et al., 2023 [93] | United Kingdom | - Main outcomes: Online access led to unintended consequences that negatively impacted patient understanding and increased GP workload. - Significant observations: Online access changed GPs' documentation practices and required additional work to manage and monitor access. Patients found it convenient, but it did not necessarily improve care efficiency. - Recommendations: Practices require support and resources to manage unintended consequences effectively. Mitigation strategies include offering consultations to potentially distressed patients and reducing the use of jargon in records. |
| **Telemedicine** | | |
| Johansson et al., 2020 [67] | Sweden | - Main outcomes: GPs had generally positive experiences with digital written patient dialogues, with good technical functionality and satisfaction with asynchronous communication. - Significant observations: Benefits included flexibility, reliability, IT safety, and effective use of photos. Concerns included predetermined symptom lists and the digital treatment of certain symptoms. - Recommendations: Further development of digital systems is necessary, accompanied by additional studies to refine the technology and assess its broader impacts on healthcare. |
| Koch and Guhres, 2020 [68] | Sweden | - Main outcomes: VCs are perceived as time-saving and flexible, but not necessarily more effective than physical consultations. Physicians have a positive attitude towards them. - Significant observations: Challenges include the need for reliable technology, effective patient triage, and limited access to decision-making information compared to physical consultations. - Recommendations: Introduce (semi)-automatic triage systems, ensure technical platform reliability, and incorporate digital skills into medical education. Younger physicians should gain experience with physical consultations before moving on to other areas. |
| Mueller et al., 2020 [69] | Germany | - Main outcomes: DHTs influence patient activation, treatment processes, patient differentiation, and patient-physician interaction. - Significant observations: Proactive patient information-seeking is beneficial, but it can also lead to misinformation, which can cause insecurity. Digital tools can reduce effort but also pose reliability challenges. Patient differentiation affects technology use. Reduced personal contact is a concern. - Recommendations: Broader research is needed to understand patient perspectives and test hypotheses related to the use of DHT. |
| Waschkau et al., 2020 [70] | Germany | - Main outcomes: The potential of telemedicine is underutilized; data safety is a major barrier; interest in telemedicine training is high. - Significant observations: Telemedicine could change doctor-patient relationships; it may support rural practice; skepticism exists about cost-effectiveness. - Recommendations: Introduce telemedicine education early; address data safety and efficacy concerns; leverage trainees as agents for implementation. |
| Hvidt et al., 2021 [71] | Denmark | - Main outcomes: Email consultations are a key development in DHT, affecting professional boundaries and the doctor-patient relationship. They are part of a dynamic socio-technical network, not just a tool for simple transactions. - Significant observations: Implementation has led to new expectations and roles, which can sometimes cause friction. Boundary setting is crucial in GP-patient communications, influenced by professional discourses. Some GPs resist imposed changes, showing agency in the adoption process. Successful practices emerge from negotiations and boundary setting within relationships. - Recommendations: Understanding multilevel interactions is essential, with recommendations for co-design involving all stakeholders for future implementations. |
| Johnsen et al., 2021 [72] | Norway | - Main outcomes: VCs were considered suitable in about half of the cases compared to face-to-face consultations. They were particularly suitable for mental health issues, administrative tasks, and chronic problems requiring follow-up. - Significant observations: The suitability of VCs is strongly associated with relational and episodic continuity. VCs are less suitable for acute and severe health issues. - Recommendations: GPs estimate that 20% of consultations could be conducted via video in a non-pandemic future. Continuity of care should be maintained to enhance the suitability of VCs. |
| Meurs et al., 2022 [73] | Netherlands | - Main outcomes: Increased use of e-health technologies during COVID-19; recognition of benefits by GPs; intention to continue use post-pandemic. - Significant observations: Need for integration into GP care; importance of user-friendliness and digital skills; challenges with IT system integration and administrative burdens. - Recommendations: Improve integration with IT systems; provide technological and financial support; conduct research on patient group needs and digital skills. |
| Turner et al., 2022 [74] | United Kingdom | - Main outcomes: Online consultations are best for simple transactions, but do not necessarily improve access to care or practice efficiency. - Significant observations: Unintended consequences include restricted patient access, increased workload, and staff dissatisfaction. These are partly due to implementation processes. - Recommendations: Recognize unintended consequences to maximize benefits and minimize harms. Include patient voices in process changes. |
| Waschkau et al., 2022 [75] | Germany | - Main outcomes: High satisfaction with telemedical applications, especially asynchronous ones. Asynchronous telemedical applications (TAs) are easier to implement and integrate into practice workflows. - Significant observations: A lack of broadband internet in rural areas is a significant barrier. Asynchronous TAs could improve access to care in rural areas with limited internet access. - Recommendations: Using existing, stable technology for successful implementation. Ensure broadband internet availability, use market-ready applications, provide adequate training, and have a technical support plan. |
| Lüchau et al., 2023 [76] | Denmark | - Main outcomes: Identification of four frames influencing GP adoption of VCs; varied adoption based on perceived advantages. - Significant observations: VCs are seen as conflicting with professional values, a crisis tool, a future technology, or a means to improve work conditions. - Recommendations: Focus on understanding GPs' interpretive frames, improve communication between politicians and GPs, and consider organizational changes in implementation strategies. |
| Peeters et al., 2023 [77] | Netherlands | - Main outcomes: Positive effects on quality of care and collaboration between GPs and specialists. Improved accessibility and efficiency of care. - Significant observations: Enhanced GP-patient relationship due to specialist-backed advice. Educational value for GPs, helping fill knowledge gaps. Asynchronous communication improves efficiency. - Recommendations: Recommendations for improvements in applicability, communication, and training. Use e-consultations to avoid unnecessary hospital referrals. Consider using e-consultations for training purposes and to identify knowledge gaps. |
| Tensen et al., 2023 [78] | Netherlands | - Main outcomes: GPs were generally satisfied with digital dermatology services but faced barriers such as limited photography skills and equipment issues. - Significant observations: Training is needed to improve equipment use and image quality; most GPs would continue to use the platform. - Recommendations: Enhance training for GPs, improve platform guidance for patient photography, and address equipment and interface issues. |
| Grønning et al., 2024 [83] | Denmark | - Main outcomes: Email consultations are intended to increase efficiency, but often lead to GPs spending more time on them, disrupting their work routines. - Significant observations: Email consultations transform power relations between GPs and patients, introduce new time sensibilities, and blur traditional boundaries. - Recommendations: Better integration of email consultations into GPs' workflows is needed, along with strategies to manage the time pressures they introduce. |
| Klausen and Hvidt, 2024 [79] | Denmark | - Main outcomes: E-consultations are a significant part of GP-patient communication, offering both opportunities for increased access and challenges in managing expectations and boundaries. - Significant observations: E-consultations foster a culture of non-stop connectivity, present ethical challenges in boundary setting, and can create a sense of GP presence even in silence. Recommendations: Further research is needed to understand the lived experiences of patients and GPs, with a focus on ethical considerations and sustainable digital care practices. |
| Kofod et al., 2024 [80] | Denmark | - Main outcomes: GPs experience challenges in perceiving non-verbal cues and maintaining interpersonal contact during VCs. They compensate by asking more questions, repeating information, and using meta-communication. - Significant observations: GPs find VCs sufficient for specific purposes but prefer them for less sensitive topics or with patients they already know. They use triage to select suitable consultations for VCs. - Recommendations: Use VCs for less sensitive consultations and with patients who have an established relationship with the GP. Focus on compensatory strategies, such as asking more questions and using meta-communication, to maintain effective communication. |
| Kollmann et al., 2024 [81] | Netherlands | - Main outcomes: Remote care, especially telephone consultations, was generally accessible for patients from low-income neighborhoods. - Significant observations: Video calling was underutilized due to unfamiliarity among patients. Remote care was deemed suitable for minor ailments but not for serious health issues. There was a lack of consensus among healthcare professionals on the appropriateness of remote care for certain complaints. Nurse practitioners and mental health professionals had a negative attitude towards remote care. - Recommendations: Integrate remote care into future healthcare systems, considering both patient and professional opinions, and develop guidelines for its appropriate use. |
| Norberg et al., 2024 [32] | Norway | - Main outcomes: Remote consultations empower GPs to design effective clinical pathways and increase workday flexibility. - Significant observations: Remote consultations may reduce patient tolerance for minor ailments, challenge GPs' gatekeeping roles, and introduce ethical dilemmas. - Recommendations: Proactive management of digitalization changes is essential, and maintaining continuity in doctor-patient relationships enhances the effectiveness of remote consultations. |
| Stamer et al., 2024 [82,] | Germany | - Main outcomes: eHealth services can effectively maintain continuity of care and improve health literacy. eHealth supports self-efficacy and patient empowerment. - Significant observations: Cooperation with health insurance companies and the incorporation of gamification elements are beneficial for eHealth implementation. Barriers include poor interface compatibility, insufficient network coverage, and a lack of digital literacy. Continuous access to care, including 24/7 contact, is beneficial. - Recommendations: Address technical barriers and enhance digital literacy. |

*Table 1: Overview of the 65 studies included in the systematic review, categorized by topic (AI, DHTs, Medical Records, and Telemedicine). Each entry includes author(s), publication year, country of study, and a summary of key findings and recommendations, highlighting trends, challenges, and implementation factors in digital health adoption across general practice settings.*

* AI: Artificial intelligence

* CDSS: Clinical decision support

* DHT: Digital health technology

* DiGA: Digitale Gesundheitsanwendung/digital health applications

* EMR: Electronic medical record

* HER: Electronic health record

* FD: Family doctor

* GP: General practitioner
* ICT: Information and Communication Technology

* LTC: long-term condition
* MA: Medical assistant

* NASSS: Non-adoption, Abandonment, and challenges to the Scale-up, Spread and Sustainability

* SDM: Secure Digital Messaging

* TA: Telemedical application

* ORA: Online Record Access
* VC: Video consultation

**References**

33.	Blease C, Kaptchuk TJ, Bernstein MH, Mandl KD, Halamka JD, DesRoches CM. Artificial intelligence and the future of primary care: exploratory qualitative study of UK general practitioners’ views. *J Med Internet Res*. 2019;21(3):e12802. doi:10.2196/12802. ISSN:1438-8871

84.	Buck C, Doctor E, Hennrich J, Jöhnk J, Eymann T. General practitioners’ attitudes toward artificial intelligence–enabled systems: interview study. *J Med Internet Res*. 2022;24(1):e28916. doi:10.2196/28916. PMID:35084342

85.	Tabla S, Calafiore M, Legrand B, et al. Artificial intelligence and clinical decision support systems or automated interpreters: what characteristics are expected by French general practitioners? In: Otero P, Scott P, Martin SZ, Huesing E, eds. *Studies in Health Technology and Informatics*. Studies in Health Technology and Informatics. IOS Press; 2022:887--891. doi:10.3233/SHTI220207. PMID:35673146. ISBN:978-1-64368-264-8

91.	Mahlknecht A, Engl A, Piccoliori G, Wiedermann CJ. Supporting primary care through symptom checking artificial intelligence: a study of patient and physician attitudes in italian general practice. *BMC Prim Care*. 2023;24(1):174-191. doi:10.1186/s12875-023-02143-0. PMID:37661285

87.	Schütze D, Holtz S, Neff MC, et al. Requirements analysis for an AI-based clinical decision support system for general practitioners: a user-centered design process. *BMC Med Inform Decis Mak*. 2023;23(1):144-153. doi:10.1186/s12911-023-02245-w. PMID:37525175

88.	Wewetzer L, Held LA, Goetz K, Steinhäuser J. Determinants of the implementation of artificial intelligence-based screening for diabetic retinopathy—a cross-sectional study with general practitioners in Germany. *Digit Health*. 2023;9:20552076231176644. doi:10.1177/20552076231176644. PMID:37274367

89.	Blease CR, Locher C, Gaab J, Hägglund M, Mandl KD. Generative artificial intelligence in primary care: an online survey of UK general practitioners. *BMJ Health Care Inform*. 2024;31(1):e101102. doi:10.1136/bmjhci-2024-101102. ISSN:2632-1009

34.	Peeters JM, Krijgsman JW, Brabers AE, Jong JDD, Friele RD. Use and uptake of eHealth in general practice: a cross-sectional survey and focus group study among health care users and general practitioners. *JMIR Med Inform*. 2016;4(2):e11. doi:10.2196/medinform.4515. ISSN:2291-9694

29.	Albrecht UV, Afshar K, Illiger K, et al. Expectancy, usage and acceptance by general practitioners and patients: exploratory results from a study in the german outpatient sector. *Digit Health*. 2017;3:2055207617695135. doi:10.1177/2055207617695135. ISSN:2055-2076, 2055-2076

35. Kriegel J, Tuttle-Weidinger L, Reckwitz L. Digital media for primary health care in Austria. Stud Health Technol Inform. 2017;236:282-289. [doi: 10.3233/978-1-61499-759-7-282] [Medline: 28508808]

36.	Cowie J, Calveley E, Bowers G, Bowers J. Evaluation of a digital consultation and self-care advice tool in primary care: a multi-methods study. *Int J Environ Res Public Health*. 2018;15(5):896-918. doi:10.3390/ijerph15050896. PMID:29724040

37.	Gabriels K, Moerenhout T. Exploring entertainment medicine and professionalization of self-care: interview study among doctors on the potential effects of digital self-tracking. *J Med Internet Res*. 2018;20(1):e10. doi:10.2196/jmir.8040. ISSN:1438-8871

30.	Morrissey EC, Glynn LG, Casey M, Walsh JC, Molloy GJ. New self-management technologies for the treatment of hypertension: general practitioners’ perspectives. *Fam Pract*. 2018;35(3):318-322. doi:10.1093/fampra/cmx100. ISSN:0263-2136, 1460-2229

38.	Torrent-Sellens J, Díaz-Chao Á, Soler-Ramos I, Saigí-Rubió F. Modeling and predicting outcomes of eHealth usage by European physicians: multidimensional approach from a survey of 9196 general practitioners. *J Med Internet Res*. 2018;20(10):e279. doi:10.2196/jmir.9253. ISSN:1438-8871

39.	Breedvelt JJ, Zamperoni V, Kessler D, et al. GPs’ attitudes towards digital technologies for depression: an online survey in primary care. *Br J Gen Pract*. 2018;69(680):e164-e170. doi:10.3399/bjgp18X700721. PMID:30559111

40.	Buhtz C, Paulicke D, Schwarz K, Jahn P, Stoevesandt D, Frese T. Receptiveness of GPs In the south of saxony-anhalt, germany to obtaining training on technical assistance systems for caregiving: a cross-sectional study. *Clin Interv Aging*. 2019;Volume 14:1649-1656. doi:10.2147/CIA.S218367. PMID:31571844

31.	Fagerlund AJ, Holm IM, Zanaboni P. General practitioners’ perceptions towards the use of digital health services for citizens in primary care: a qualitative interview study. *BMJ Open*. 2019;9(5):e028251. doi:10.1136/bmjopen-2018-028251. PMID:31061056

41.	Klocek A, Šmahelová M, Knapová L, Elavsky S. GPs’ perspectives on eHealth use in the Czech Republic: a cross-sectional mixed-design survey study. *BJGP Open*. 2019;3(3):bjgpopen19X101655. doi:10.3399/bjgpopen19X101655. ISSN:2398-3795

42.	Entezarjou A, Bolmsjö BB, Calling S, Midlöv P, Milos Nymberg V. Experiences of digital communication with automated patient interviews and asynchronous chat in swedish primary care: a qualitative study. *BMJ Open*. 2020;10(7):e036585. doi:10.1136/bmjopen-2019-036585. ISSN:2044-6055, 2044-6055

43.	Poss-Doering R, Kuehn L, Kamradt M, Glassen K, Wensing M. Applying digital information delivery to convert habits of antibiotic use in primary care in Germany: mixed-methods study. *J Med Internet Res*. 2020;22(10):e18200. doi:10.2196/18200. PMID:32960773

44.	Glock H, Milos Nymberg V, Borgström Bolmsjö B, et al. Attitudes, barriers, and concerns regarding telemedicine among swedish primary care physicians: a qualitative study. *Int J Gen Med*. 2021;Volume 14:9237-9246. doi:10.2147/IJGM.S334782. PMID:34880663

45.	Jakobsen PR, Christensen JR, Nielsen JB, et al. Identification of important factors affecting use of digital individualised coaching and treatment of type 2 diabetes in general practice: a qualitative feasibility study. *Int J Environ Res Public Health*. 2021;18(8):3924. doi:10.3390/ijerph18083924. PMID:33917999

46.	Keuper J, Batenburg R, Verheij R, Van Tuyl L. Use of E-health in dutch general practice during the COVID-19 pandemic. *Int J Environ Res Public Health*. 2021;18(23):12479. doi:10.3390/ijerph182312479. PMID:34886204

47.	Knop M, Mueller M, Niehaves B. Investigating the use of telemedicine for digitally mediated delegation in team-based primary care: mixed methods study. *J Med Internet Res*. 2021;23(8):e28151. doi:10.2196/28151. ISSN:1438-8871

48.	Pikkemaat M, Thulesius H, Milos Nymberg V. Swedish primary care physicians’ intentions to use telemedicine: a survey using a new questionnaire – physician attitudes and intentions to use telemedicine (PAIT). *Int J Gen Med*. 2021;Volume 14:3445-3455. doi:10.2147/IJGM.S319497. PMID:34295177

49.	Bondaronek P, Dicken SJ, Singh Jennings S, Mallion V, Stefanidou C. Barriers to and facilitators of the use of digital tools in primary care to deliver physical activity advice: semistructured interviews and thematic analysis. *JMIR Hum Factors*. 2022;9(3):e35070. doi:10.2196/35070. ISSN:2292-9495

50.	Hammerton M, Benson T, Sibley A. Readiness for five digital technologies in general practice: perceptions of staff in one part of southern England. *BMJ Open Qual*. 2022;11(2):e001865. doi:10.1136/bmjoq-2022-001865. ISSN:2399-6641

51.	Van De Vijver S, Hummel D, Van Dijk AH, et al. Evaluation of a digital self-management platform for patients with chronic illness in primary care: qualitative study of stakeholders’ perspectives. *JMIR Form Res*. 2022;6(8):e38424. doi:10.2196/38424. ISSN:2561-326X

52.	Butler K, Bartlett YK, Newhouse N, et al. Implementing a text message-based intervention to support type 2 diabetes medication adherence in primary care: a qualitative study with general practice staff. *BMC Health Serv Res*. 2023;23(1):614-623. doi:10.1186/s12913-023-09571-9. PMID:37301867

53.	Kujansivu K, Tolvanen E, Kautto M, Koskela TH. The use of digital tools by general practitioners in finnish public health centres. *Finn J EHealth EWelfare*. 2023;15(1):40-51-40-51. doi:10.23996/fjhw.122703. ISSN:1798-0798

54.	Solberg Carlsson K, Øvretveit J, Ohrling M. Rapid implementation of remote digital primary care in Stockholm and implications for further system-wide implementation: practitioner’s and manager’s experience of the always open mobile application. *Scand J Prim Health Care*. 2023;41(3):232-246. doi:10.1080/02813432.2023.2229387. PMID:37470469

55.	Wangler J, Jansky M. Two years of approved digital health applications in Germany – perspectives and experiences of general practitioners with an affinity for their use. *Eur J Gen Pract*. 2023;29(1):2186396. doi:10.1080/13814788.2023.2186396. PMID:36919621

56.	Brecher AL, Herrgen J, Mueller T, et al. DiGA in primary care and the influence of patient-related factors – results of a survey of family doctors. In: Hayn D, Pfeifer B, Schreier G, Baumgartner M, eds. *Studies in Health Technology and Informatics*. Vol 313. 1st ed. IOS Press; 2024:55-61. doi:10.3233/SHTI240012. PMID:38682505. ISBN:978-1-64368-516-8

57.	Gavin JP, Clarkson P, Muckelt PE, et al. Healthcare professional and commissioners’ perspectives on the factors facilitating and hindering the implementation of digital tools for self-management of long-term conditions within UK healthcare pathways. Fuller SS, ed. *PLOS ONE*. 2024;19(8):e0307493. doi:10.1371/journal.pone.0307493. ISSN:1932-6203

58.	Ghis Malfilatre M, Louvel S. A medical profession towards empowerment? The uncertain future of digitalization in private general practice. *Soc Sci Med*. 2024;365(ut9, 8303205):117575. doi:10.1016/j.socscimed.2024.117575. ISSN:02779536

59.	Nassehi D, Ramvi E. Care in the era of digital health: experiences from norwegian general practitioners. *Scand J Prim Health Care*. 2024;43(2):338-346. doi:10.1080/02813432.2024.2437417. PMID:39639473

60.	Newbould J, Hocking L, Sidhu M, Daniel K. Digital first primary care for those with multiple long-term conditions: a rapid review of the views of stakeholders. *Health Soc Care Deliv Res*. 2024;12(21):1-68. doi:10.3310/AWBT4827. PMID:39056123

61.	Posselt J, Lander J, Dierks ML. Digitale gesundheitsanwendungen in der hausärztlichen versorgung: eine diskussionsgrundlage zur förderung informierter nutzungsentscheidungen. *Prävent Gesundheitsförderung*. 2024;19(4):483-489. doi:10.1007/s11553-024-01126-y. ISSN:1861-6755, 1861-6763

62.	Potthoff S, Brendryen H, Bosnic H, Njå ALM, Finch T, Lid TG. Recommendations for implementing digital alcohol interventions in primary care: lessons learned from a norwegian feasibility study. *Front Health Serv*. 2024;4:1343568. doi:10.3389/frhs.2024.1343568. ISSN:2813-0146

63.	Preiser C, Radionova N, Ög E, et al. The doctors, their patients, and the symptom checker app: qualitative interview study with general practitioners in Germany. *JMIR Hum Factors*. 2024;11:e57360. doi:10.2196/57360. ISSN:2292-9495

64.	Schendzielorz J, Harre K, Tarara M, Oess S, Holmberg C. Digitale technologien in der allgemeinmedizin: aktueller stand und zukünftige anforderungen. *Z Für Allg*. 2024;100(1):21-29. doi:10.1007/s44266-023-00145-w. ISSN:1433-6251, 1439-9229

65.	Wangler J, Jansky M. How can primary care benefit from digital health applications? – a quantitative, explorative survey on attitudes and experiences of general practitioners in Germany. *BMC Digit Health*. 2024;2(1):14-28. doi:10.1186/s44247-024-00068-x. ISSN:2731-684X

66.	Weik L, Fehring L, Mortsiefer A, Meister S. Understanding inherent influencing factors to digital health adoption in general practices through a mixed-methods analysis. *Npj Digit Med*. 2024;7(1):47-62. doi:10.1038/s41746-024-01049-0. PMID:38413767

90.	Cabitza, Del Zotti, Misericordia. Electronic records for general practice - where we are, where we should head to improve them: In: *Proceedings of the {HEALTHINF} 2014 - Proceedings of the International Conference on Health Informatics*. SCITEPRESS - Science and and Technology Publications; 2014:535-542. doi:10.5220/0004924805350542. ISBN:978-989-758-010-9

91.	Blease C, Torous J, Dong Z, et al. Patient online record access in english primary care: qualitative survey study of general practitioners’ views. *J Med Internet Res*. 2023;25:1-13. doi:10.2196/43496. PMID:36811939

92.	Davidge G, Brown L, Lyons M, et al. Primary care staff’s views and experience of patients’ online access to their electronic health record: a qualitative exploration. *Br J Gen Pract*. 2023;73(731):e418-e426. doi:10.3399/BJGP.2022.0436. PMID:37068967

93.	Turner A, Morris R, McDonagh L, et al. Unintended consequences of patient online access to health records: a qualitative study in UK primary care. *Br J Gen Pract*. 2022;73(726):e67-e74. doi:10.3399/BJGP.2021.0720. PMID:36316163

67.	Johansson A, Larsson M, Ivarsson B. General practitioners’ experiences of digital written patient dialogues: a pilot study using a mixed method. *J Prim Care Community Health*. 2020;11:2150132720909656. doi:10.1177/2150132720909656. ISSN:2150-1319, 2150-1327

68. Koch S, Guhres M. Physicians’ experiences of patient-initiated online consultations in primary care using direct-to-consumer technology. Studies in Health Technology and Informatics. Jun 16, 2020;270:643-647. [doi: 10.3233/ SHTI200239] [Medline: 32570462]

69.	Müller M, Knop M, Ressing C, et al. Constituting factors of a digitally influenced relationship between patients and primary care physicians in rural areas. In: *Proceedings of the Annual Hawaii International Conference on System Sciences*. Hawaii International Conference on System Sciences; 2020. doi:10.24251/HICSS.2020.447

70.	Waschkau A, Flägel K, Goetz K, Steinhäuser J. Evaluation of attitudes towards telemedicine as a basis for successful implementation: a cross-sectional survey among postgraduate trainees in family medicine in Germany. *Z Für Evidenz Fortbild Qual Im Gesundheitswesen*. 2020;156-157:75-81. doi:10.1016/j.zefq.2020.07.001. PMID:32859557

71.	Hvidt EA, Grønning A, Nisbeth Brøgger M, Møller JE, Fage-Butler A. Multilevel structures and human agency in relation to email consultations: a strong structuration theory analysis of the danish general practice setting. *Soc Sci Med*. 2021;282:114155. doi:10.1016/j.socscimed.2021.114155. ISSN:02779536

72.	Johnsen TM, Norberg BL, Kristiansen E, et al. Suitability of video consultations during the COVID-19 pandemic lockdown: cross-sectional survey among norwegian general practitioners. *J Med Internet Res*. 2021;23(2):e26433. doi:10.2196/26433. ISSN:1438-8871

73.	Meurs M, Keuper J, Sankatsing V, Batenburg R, Van Tuyl L. “Get used to the fact that some of the care is really going to take place in a different way”: general practitioners’ experiences with E-health during the COVID-19 pandemic. *Int J Environ Res Public Health*. 2022;19(9):5120. doi:10.3390/ijerph19095120. PMID:35564519

74.	Turner A, Morris R, Rakhra D, et al. Unintended consequences of online consultations: a qualitative study in UK primary care. *Br J Gen Pract*. 2021;72(715):e128-e137. doi:10.3399/BJGP.2021.0426. PMID:34903520

75.	Waschkau A, Traulsen P, Steinhäuser J. Evaluation of synchronous and asynchronous telemedical applications in primary care in rural regions of northern Germany—results and lessons learned from a pilot study. *Int J Environ Res Public Health*. 2022;19(22):14860. doi:10.3390/ijerph192214860. ISSN:1660-4601

76.	Lüchau EC, Atherton H, Olesen F, Søndergaard J, Hvidt EA. Interpreting technology: use and non-use of doctor-patient video consultations in danish general practice. *Soc Sci Med*. 2023;334:116215. doi:10.1016/j.socscimed.2023.116215. ISSN:02779536

77.	Peeters K, Giroldi E, Veldhuizen L, Abdelaziz K, Muris D, Cals J. General practitioner use of e-consultation to consult hospital specialists: interview study to obtain physician’s perceptions about digital interprofessional communication. *J Med Internet Res*. 2023;25:e40318. doi:10.2196/40318. PMID:36976626

78.	Tensen E, Kuziemsky C, Jaspers MW, Peute LW. General practitioners’ perspectives about remote dermatology care during the COVID-19 pandemic in the Netherlands: questionnaire-based study. *JMIR Dermatol*. 2023;6(1):e46682. doi:10.2196/46682. ISSN:2562-0959

83.	Grønning A, Simonsen LM, Lüchau EC, Hvidt EA, Klausen M. My time, your time, our time. Older patients’ and GPs’ time sensibilities around email consultations. *Health Sociol Rev*. 2024;33(1):43-58. doi:10.1080/14461242.2024.2316742. ISSN:1446-1242, 1839-3551

79.	Klausen M, Hvidt EA. E-consultation as existential media: exploring doctor-patient ‘digital thrownness’ in danish general practice. *Sociol Health Illn*. 2024;46(8):1849-1863. doi:10.1111/1467-9566.13823. PMID:39088382

80.	Kofod FG, Hvidt EA, Arreskov AB, Guassora AD. Interpersonal contact and altered sensory conditions in video consultation – a qualitative interview study in danish general practice. *Scand J Prim Health Care*. 2024;42(4):650-658. doi:10.1080/02813432.2024.2376744. ISSN:0281-3432, 1502-7724

81.	Kollmann J, Sana S, Magnée T, et al. Patients’ and professionals’ experiences with remote care during COVID-19: a qualitative study in general practices in low-income neighborhoods. *Prim Health Care Res Dev*. 2024;25:e32. doi:10.1017/S1463423624000240. PMID:38826073

32.	Norberg BL, Austad B, Kristiansen E, Zanaboni P, Getz LO. The impact and wider implications of remote consultations for general practice in Norway: qualitative study among norwegian contract general practitioners. *JMIR Form Res*. 2024;8:e63068. doi:10.2196/63068. ISSN:2561-326X

82.	Stamer T, Traulsen P, Rieken J, Schmahl T, Menrath I, Steinhäuser J. Determinants of the implementation of eHealth-based long-term follow-up care for young cancer survivors: a qualitative study. *BMC Cancer*. 2024;24(1):1159. doi:10.1186/s12885-024-12910-6. PMID:39294671

**Abbreviations**

AI: Artificial intelligence

CDSS: Clinical decision support system

DHA: Digital health application

DHT: Digital health tool

EHR: Electronic health record

EMR: Electronic medical record

GP: General practitioner

ICT: Information and communication technology

LTC: Long-term condition

TM: Telemedicine

UK: United Kingdom

VC: Video consultation
