# Supplementary material for: The Impact of Digitalization on European General Practice From the Perspective of General Practitioners: Systematic Review
Source: J Med Internet Res. 2026 Aug 18;28:e83343. doi: 10.2196/83343 (PMC13485116; doi:10.2196/83343)
Supplement: Supplementary file 5 [file jmir-v28-e83343-s006.docx]

**MMAT Scores and Risk of Bias Assessment**

**MMAT Scores**

| **Reference** | **Research Question** | | **Methodology** | **MMAT %** |
| --- | --- | --- | --- | --- |
|  | Clear RQ? | Objectives? |  |  |
| Albrecht et al., 2017 [29] | Y | Y | 4 - Quantitative descriptive | 80 |
| Hvidt et al., 2021 [71] | Y | Y | 1 - Qualitative | 100 |
| Blease et al., 2019 [33] | Y | Y | 1 - Qualitative | 80 |
| Blease et al., 2023 [91] | Y | Y | 1 - Qualitative | 100 |
| Blease et al., 2024 [89] | Y | Y | 4 - Quantitative descriptive | 80 |
| Bondaronek et al., 2022 [49] | Y | Y | 1 - Qualitative | 100 |
| Brecher et al., 2024 [56] | Y | Y | 4 - Quantitative descriptive | 80 |
| Breedvelt et al., 2019 [39] | Y | Y | 4 - Quantitative descriptive | 80 |
| Buck et al., 2022 [84] | Y | Y | 1 - Qualitative | 100 |
| Buhtz et al., 2019 [40] | Y | Y | 4 - Quantitative descriptive | 80 |
| Butler et al., 2023 [52] | Y | Y | 1 - Qualitative | 100 |
| Cabitza et al., 2014 [90] | Y | Y | 4 - Quantitative descriptive | 80 |
| Cowie et al., 2018 [36] | Y | Y | 5 - Mixed methods | 80 |
| Davidge et al., 2023 [92] | Y | Y | 1 - Qualitative studies | 100 |
| Entezarjou et al., 2020 [42] | Y | Y | 1 - Qualitative studies | 100 |
| Fagerlund et al., 2019 [31] | Y | Y | 1 - Qualitative studies | 100 |
| Gabriels and Moerenhout, 2018 [37] | Y | Y | 1 - Qualitative | 100 |
| Gavin et al., 2024 [57] | Y | Y | 1 - Qualitative | 80 |
| Ghis Malfilatre and Louvel, 2025 [58] | Y | Y | 1 - Qualitative | 100 |
| Glock et al., 2021 [44] | Y | Y | 1 - Qualitative | 80 |
| Grønning et al., 2024 [83] | Y | Y | 1 - Qualitative | 100 |
| Hammerton et al., 2022 [50] | Y | Y | 5 - Mixed methods | 80 |
| Jakobsen et al., 2021 [45] | Y | Y | 1 - Qualitative | 80 |
| Johansson et al., 2020 [67] | Y | Y | 5 - Mixed methods | 80 |
| Johnsen et al., 2021 [72] | Y | Y | 4 - Quantitative descriptive | 80 |
| Keuper et al., 2021 [46] | Y | Y | 4 - Quantitative descriptive | 80 |
| Klausen and Hvidt, 2024 [79] | Y | Y | 1 - Qualitative | 100 |
| Klocek et al., 2019 [41] | Y | Y | 5 - Mixed methods | 80 |
| Knop et al., 2021 [47] | Y | Y | 5 - Mixed methods | 80 |
| Koch and Guhres, 2020 [68] | Y | Y | 4 - Quantitative descriptive | 60 |
| Kofod et al., 2024 [80] | Y | Y | 1 - Qualitative | 100 |
| Kollmann et al., 2024 [81] | Y | Y | 1 - Qualitative | 80 |
| Kriegel et al., 2017 [35] | C | C | 4 - Quantitative descriptive | 60 |
| Kujansivu et al., 2023 [53] | Y | Y | 4 - Quantitative descriptive | 80 |
| Lüchau et al., 2023 [76] | Y | Y | 1 - Qualitative studies | 100 |
| Mahlknecht et al., 2023 [86] | Y | Y | 4 - Quantitative descriptive | 80 |
| Meurs et al., 2022 [73] | Y | Y | 1 - Qualitative | 80 |
| Morrissey et al., 2018 [30] | Y | Y | 1 - Qualitative | 100 |
| Mueller et al., 2020 [69] | Y | Y | 1 - Qualitative | 80 |
| Nassehi and Ramvi, 2024 [59] | Y | Y | 1 - Qualitative | 100 |
| Newbould et al., 2024 [60] | Y | Y | 1 - Qualitative studies | 80 |
| Norberg et al., 2024 [32] | Y | Y | 1 - Qualitative | 80 |
| Peeters et al., 2016 [34] | Y | Y | 5 - Mixed methods | 80 |
| Peeters et al., 2023 [77] | Y | Y | 1 - Qualitative | 80 |
| Pikkemaat et al., 2021 [48] | Y | Y | 4 - Quantitative descriptive | 60 |
| Poss-Doering et al., 2020 [43] | Y | Y | 5 - Mixed methods studies | 80 |
| Posselt et al., 2024 [61] | Y | Y | 1 - Qualitative | 80 |
| Potthoff et al., 2024 [62] | Y | Y | 1 - Qualitative | 80 |
| Preiser et al., 2024 [63] | Y | Y | 1 - Qualitative | 80 |
| Schendzielorz et al., 2023 [64] | Y | Y | 4 - Quantitative descriptive | 60 |
| Schütze et al., 2023 [87] | Y | Y | 1 - Qualitative | 80 |
| Solberg Carlsson et al., 2023 [54] | Y | Y | 1 - Qualitative studies | 100 |
| Stamer et al., 2024 [82] | Y | Y | 1 - Qualitative studies | 80 |
| Tabla et al., 2022 [85] | Y | Y | 4 - Quantitative descriptive | 80 |
| Tensen et al., 2023 [78] | Y | Y | 5 - Mixed methods | 80 |
| Torrent-Sellens et al., 2018 [38] | Y | Y | 4 - Quantitative descriptive | 80 |
| Turner et al., 2022 [74] | Y | Y | 1 - Qualitative | 100 |
| Turner et al., 2023 [93] | Y | Y | 1 - Qualitative | 100 |
| Van De Vijver et al., 2022 [51] | Y | Y | 1 - Qualitative | 80 |
| Wangler and Jansky, 2023 [55] | Y | Y | 1 - Qualitative | 80 |
| Wangler and Jansky, 2024 [65] | Y | Y | 4 - Quantitative descriptive studies | 80 |
| Waschkau et al., 2020 [70] | Y | Y | 4 - Quantitative descriptive studies | 80 |
| Waschkau et al., 2022 [75] | Y | Y | 4 - Quantitative descriptive | 80 |
| Weik et al., 2024 [66] | Y | Y | 5 - Mixed methods studies | 100 |
| Wewetzer et al., 2023 [88] | Y | Y | 4 - Quantitative descriptive | 80 |

**MMAT Key:**

Y: Yes;

N: No;

C: Can’t tell

R: Review

**Study Characteristics Table**

| **Study Type** | | **Reference/ Code** |
| --- | --- | --- |
| **Qualitative** | | |
| **1.** | Case studies/management/ review of medical records/ messaging analysis/ Interviews/ Focus groups **(n= 36)** | [1–36] |
| **Quantitative RCT** | | |
| **2.** | Randomised Control Trials (RCT) **(n=0)** |  |
| **Quantitative, non-randomised** | | |
| **3.** | Quasi-experimental (trial and survey) (open and controlled studies, sequential control design)/ Cohort studies (retrospective, prospective, and longitudinal)/ Cross-sectional/ analysis including various modes of delivery; telephone, online). Retrospective case control studies. Sequential control design. **(n=0)** |  |
| **Quantitative descriptive studies** | | |
| **4** | Quantitative, descriptive study (pilot study reporting service use and frequencies, survey/ analysis, descriptive vignette study, quantitative analysis of medical records/ secure messaging, retrospective survey) **(n=20)** | [37–56] |
| **Mixed Methods** | | |
| **5.** | Mixed Methods (interviews, focus groups, and quantitative analysis of based system utilisation data/ survey data) **(n= 9)** | [57–65] |
| **Reviews** | | |
|  | Interpretative/ Literature Reviews **(n=0)** |  |

**Vote counting analysis**

| **60% MMAT Study** | **Major Claims** | **Supporting Studies (≥80% quality)** | **Strength** |
| --- | --- | --- | --- |
| Koch and Guhres, 2020 [68] | Video consultations time-saving | 17 studies | Strong |
|  | Positive physician attitudes | 50 studies | Overwhelming |
|  | Implementation challenges | 60 studies | Universal |
|  | Technology reliability needed | 58 studies | Universal |
| Kriegel et al., 2017 [35] | Conservative digital media use | 25 studies | Moderate-Strong |
|  | Benefits for coordination | 26 studies | Moderate-Strong |
|  | Legal/structural barriers | 60 studies | Universal |
| Pikkemaat et al., 2021 [48] | Use-intention gap | 47 studies | Strong |
|  | Attitudes predict adoption | 60 studies | Universal |
| Schendzielorz et al., 2024 [64] | Data protection concerns | 60 studies | Universal |
|  | Role replacement fears | 37 studies | Moderate-Strong |
|  | Technology should complement | 41 studies | Strong |

**Risk of Bias Assessment - JBI**

Risk of bias was assessed for all 65 included studies using the appropriate JBI critical appraisal tools: the JBI Checklist for Analytical Cross-Sectional Studies (8 items) for 16 studies, the JBI Checklist for Qualitative Research (10 items) for 31 studies, and both tools in combination for 18 mixed-methods studies. For cross-sectional and mixed-methods studies, Item 2 (standard criteria for measuring a condition) was rated as not applicable across all studies because the research question concerned attitudes and perceptions rather than clinical diagnosis.

Overall, the majority of studies were judged to have low to moderate risk of bias. Three studies were rated with high risk (<60% YES). Those were all short conference proceedings papers, in which methodological transparency is structurally constrained by the format rather than reflecting fundamental design flaws.

The most common limitations across study types were as follows. In qualitative and mixed-methods studies, the predominant weakness was limited reporting of researcher reflexivity and positionality (JBI Items 6 and 7), with the majority of studies receiving No or Unclear on these criteria. Ethical approval was occasionally not explicitly reported, particularly in shorter papers. In cross-sectional and survey-based studies, the most frequent concerns were unclear identification and handling of confounding factors (Items 5 and 6) and limited reporting of measurement validity for exposure and outcome variables. Response bias inherent to convenience sampling was acknowledged in most studies but rarely formally addressed through sensitivity analyses or weighting.

These patterns are consistent with the methodological norms of implementation and perception research in primary care, where large-scale randomized or controlled designs are neither feasible nor appropriate for the research questions being addressed. The identified limitations do not invalidate the inclusion of any study, as the core findings — GP attitudes, experiences, and perceptions of digital health tools — remain interpretable within the stated study designs. Findings from high-risk studies were interpreted with additional caution and were not used as the sole basis for any key conclusion. Excluding these studies did not change the overall results in the TAM and NPT analyses.

Table: JBI Risk-of-Bias Assessment – Qualitative Research

*JBI Critical Appraisal Checklist for Qualitative Research*

***Q1:*** *Philosophical perspective & methodology congruent |* ***Q2:*** *Methodology & research question congruent |* ***Q3:*** *Methodology & data collection congruent |* ***Q4:*** *Methodology & data representation/analysis congruent |* ***Q5:*** *Methodology & interpretation congruent |* ***Q6:*** *Researcher located culturally/theoretically |* ***Q7:*** *Researcher influence addressed |* ***Q8:*** *Participant voices adequately represented |* ***Q9:*** *Ethical approval/considerations |* ***Q10:*** *Conclusions flow from analysis*

*Risk thresholds: Low risk ≥ 80% YES | Moderate risk 60–79% YES | High risk < 60% YES*

*YES = Criterion clearly met based on full text review | NO = Criterion clearly not met | UNCLEAR = Insufficient information to judge | NA = Not applicable*

*• Q6 (researcher located culturally/theoretically) & Q7 (researcher influence addressed) were most often absent — the majority of studies do not include an explicit reflexivity or positionality statement.*

*• Q1 (philosophical perspective) was 'Unclear' for most studies, as many did not explicitly name an epistemological framework; methodology was inferred from methods described.*

| **Author & Year** | **Q1** | **Q2** | **Q3** | **Q4** | **Q5** | **Q6** | **Q7** | **Q8** | **Q9** | **Q10** | **YES (%)** | **Risk Level** |
| --- | --- | --- | --- | --- | --- | --- | --- | --- | --- | --- | --- | --- |
| Hvidt et al., 2021 [71] | Yes | Yes | Yes | Yes | Yes | Yes | Unclear | Yes | Yes | Yes | **90%** | **Low risk** |
| Bondaronek et al., 2022 [49] | Unclear | Yes | Yes | Yes | Yes | No | No | Yes | Yes | Yes | 70% | Moderate risk |
| Buck et al., 2022 [84] | Yes | Yes | Yes | Yes | Yes | No | No | Yes | Unclear | Yes | **70%** | **Moderate risk** |
| Butler et al., 2023 [52] | Yes | Yes | Yes | Yes | Yes | Yes | Yes | Yes | Yes | Yes | **100%** | **Low risk** |
| Davidge et al., 2023 [92] | Unclear | Yes | Yes | Yes | Yes | No | No | Yes | Yes | Yes | **70%** | **Moderate risk** |
| Entezarjou et al., 2020 [42] | Yes | Yes | Yes | Yes | Yes | Yes | Yes | Yes | Yes | Yes | **100%** | **Low risk** |
| Fagerlund et al., 2019 [31] | Unclear | Yes | Yes | Yes | Yes | No | No | Yes | Yes | Yes | **70%** | **Moderate risk** |
| Gabriels & Moerenhout, 2018 [37] | Unclear | Yes | Yes | Yes | Yes | No | No | Yes | Unclear | Yes | **60%** | **Moderate risk** |
| Gavin et al., 2024 [57] | Unclear | Yes | Yes | Yes | Yes | No | No | Yes | Yes | Yes | **70%** | **Moderate risk** |
| Malfilatre and Louvel, 2025 [58] | Unclear | Yes | Yes | Yes | Yes | Unclear | No | Yes | Unclear | Yes | **60%** | **Moderate risk** |
| Glock et al., 2021 [44] | Unclear | Yes | Yes | Yes | Yes | Yes | Yes | Yes | Yes | Yes | **90%** | **Low risk** |
| Grønning et al., 2024 [83] | Yes | Yes | Yes | Yes | Yes | Yes | Yes | Yes | Yes | Yes | **100%** | **Low risk** |
| Jakobsen et al., 2021 [45] | Unclear | Yes | Yes | Yes | Yes | No | No | Yes | Yes | Yes | **70%** | **Moderate risk** |
| Klausen & Hvidt, 2024 [79] | Yes | Yes | Yes | Yes | Yes | Yes | Unclear | Yes | Yes | Yes | **90%** | **Low risk** |
| Kofod et al., 2024 [80] | Yes | Yes | Yes | Yes | Yes | Yes | Yes | Yes | Yes | Yes | **100%** | **Low risk** |
| Kollmann et al., 2024 [81] | Unclear | Yes | Yes | Yes | Yes | No | No | Yes | Yes | Yes | **70%** | **Moderate risk** |
| Lüchau et al., 2023 [76] | Yes | Yes | Yes | Yes | Yes | Yes | Yes | Yes | Yes | Yes | **100%** | **Low risk** |
| Meurs et al., 2022 [73] | Unclear | Yes | Yes | Yes | Yes | No | No | Yes | Yes | Yes | **70%** | **Moderate risk** |
| Morrissey et al., 2018 [30] | Unclear | Yes | Yes | Yes | Yes | No | No | Yes | Unclear | Yes | **60%** | **Moderate risk** |
| Mueller et al., 2020 [69] | Yes | Yes | Yes | Yes | Yes | No | No | Yes | Unclear | Yes | **70%** | **Moderate risk** |
| Nassehi and Ramvi, 2024 [59] | Unclear | Yes | Yes | Yes | Yes | No | No | Yes | Yes | Yes | **70%** | **Moderate risk** |
| Newbould et al., 2024 [60] | Unclear | Yes | Yes | Yes | Yes | No | No | Yes | Unclear | Yes | **60%** | **Moderate risk** |
| Norberg et al., 2024 [32] | Yes | Yes | Yes | Yes | Yes | Yes | Yes | Yes | Yes | Yes | **100%** | **Low risk** |
| Peeters et al., 2023 [77] | Unclear | Yes | Yes | Yes | Yes | No | No | Yes | Yes | Yes | **70%** | **Moderate risk** |
| Posselt et al., 2024 [63] | Unclear | Yes | Yes | Yes | Yes | No | No | Yes | Unclear | Yes | **60%** | **Moderate risk** |
| Schütze et al., 2023 [87] | Unclear | Yes | Yes | Yes | Yes | No | No | Yes | Yes | Yes | **70%** | **Moderate risk** |
| Solberg Carlsson et al., 2023 [54] | Unclear | Yes | Yes | Yes | Yes | No | No | Yes | Yes | Yes | **70%** | **Moderate risk** |
| Stamer et al., 2024 [82] | Unclear | Yes | Yes | Yes | Yes | No | No | Yes | Yes | Yes | **70%** | **Moderate risk** |
| Turner et al., 2022 [74] | Unclear | Yes | Yes | Yes | Yes | No | No | Yes | Yes | Yes | **70%** | **Moderate risk** |
| Turner et al., 2023 [93] | Unclear | Yes | Yes | Yes | Yes | No | No | Yes | Yes | Yes | **70%** | **Moderate risk** |
| Wangler and Jansky, 2023  [55] | Unclear | Yes | Yes | Yes | Yes | No | No | Yes | Unclear | Yes | **60%** | **Moderate risk** |

Table: JBI Risk-of-Bias Assessment – Analytical Cross-Sectional Studies

*JBI Critical Appraisal Checklist for Analytical Cross-Sectional Studies*

***Q1****: Inclusion criteria clearly defined? |* ***Q2****: Objective/standard criteria for measurement of the condition? (NA for all — attitude surveys) |* ***Q3****: Exposure measured in a valid and reliable way? |* ***Q4****: Outcomes measured in a valid and reliable way? |* ***Q5****: Confounding factors identified? |* ***Q6****: Strategies to deal with confounders stated? |* ***Q7****: Appropriate statistical analysis used? |* ***Q8****: Study subjects and setting described in detail?*

*Risk thresholds: Low risk ≥ 80% YES | Moderate risk 60–79% YES | High risk < 60% YES*

*YES = Criterion clearly met based on full text review | NO = Criterion clearly not met | UNCLEAR = Insufficient information to judge | NA = Not applicable*

*• All studies are attitude/perception surveys of GPs — there is no medical "condition" being diagnosed. Therefore Q2 = NA for all studies, reducing the denominator to 7 applicable questions per study.*

| **Author & Year** | **Q1** | **Q2** | **Q3** | **Q4** | **Q5** | **Q6** | **Q7** | **Q8** | **YES %** | **Risk Level** |
| --- | --- | --- | --- | --- | --- | --- | --- | --- | --- | --- |
| Albrecht et al., 2017 [29] | Yes | NA | Yes | Yes | Yes | Yes | Yes | Yes | **86%** | **Low risk** |
| Blease et al., 2024 [89] | Yes | NA | Yes | Yes | Yes | Unclear | Yes | Yes | **71%** | **Moderate risk** |
| Brecher et al., 2024 [53] | Yes | NA | Yes | Yes | Yes | Unclear | Yes | Yes | **71%** | **Moderate risk** |
| Breedvelt et al., 2019 [39] | Yes | NA | Yes | Yes | Yes | Unclear | Yes | Yes | **71%** | **Moderate risk** |
| Buhtz et al., 2019 [40] | Yes | NA | Yes | Yes | Yes | Unclear | Yes | Yes | **71%** | **Moderate risk** |
| Cabitza et al., 2014 [90] | Unclear | NA | Unclear | Unclear | No | No | Unclear | Yes | **14%** | **High risk** |
| Johnsen et al., 2021 [72] | Yes | NA | Yes | Yes | Yes | Yes | Yes | Yes | **86%** | **Low risk** |
| Keuper et al., 2021 [46] | Yes | NA | Yes | Yes | Yes | Unclear | Yes | Yes | **71%** | **Moderate risk** |
| Kujansivu et al., 2023 [53] | Yes | NA | Yes | Yes | Yes | Unclear | Yes | Yes | **71%** | **Moderate risk** |
| Mahlknecht et al., 2023 [86] | Yes | NA | Yes | Yes | Yes | Unclear | Yes | Yes | **71%** | **Moderate risk** |
| Pikkemaat et al., 2021 [48] | Yes | NA | Yes | Yes | Yes | Yes | Yes | Yes | **86%** | **Low risk** |
| Tabla et al., 2022 [85] | Yes | NA | Yes | Yes | Unclear | Unclear | Yes | Unclear | **43%** | **High risk** |
| Torrent-Sellens et al., 2018 [38] | Yes | NA | Yes | Yes | Yes | Yes | Yes | Yes | **100%** | **Low risk** |
| Waschkau et al., 2020 [70] | Yes | NA | Yes | Yes | Yes | Unclear | Yes | Yes | **71%** | **Moderate risk** |
| Waschkau et al., 2022 [75] | Yes | NA | Yes | Yes | Yes | Unclear | Yes | Yes | **71%** | **Moderate risk** |
| Wewetzer et al., 2023 [88] | Yes | NA | Yes | Yes | Yes | Unclear | Yes | Yes | **71%** | **Moderate risk** |

Table: JBI Risk-of-Bias Assessment — Mixed Methods Studies
*Both tools applied: JBI Analytical Cross-Sectional (Q1–Q8) + JBI Qualitative Research (Q1q–Q10q)
Risk thresholds: Low risk ≥ 80% YES | Moderate risk 60–79% YES | High risk < 60% YES
YES = Criterion clearly met based on full text review | NO = Criterion clearly not met | UNCLEAR = Insufficient information to judge | NA = Not applicable*
*CS-Q2 = NA for all studies (attitude/perception surveys, no clinical condition). YES% calculated over all non-NA answers across both tools combined.*

| **Author & Year** | **CS-Q1** | **CS-Q2** | **CS-Q3** | **CS-Q4** | **CS-Q5** | **CS-Q6** | **CS-Q7** | **CS-Q8** | **Q1q** | **Q2q** | **Q3q** | **Q4q** | **Q5q** | **Q6q** | **Q7q** | **Q8q** | **Q9q** | **Q10q** | **YES %** | **Risk Level** |
| --- | --- | --- | --- | --- | --- | --- | --- | --- | --- | --- | --- | --- | --- | --- | --- | --- | --- | --- | --- | --- |
| Blease et al., 2019 [33] | Yes | NA | Yes | Yes | Yes | Unclear | Yes | Yes | Unclear | Yes | Yes | Yes | Yes | No | Yes | Yes | Yes | Yes | **82%** | **Low risk** |
| Blease et al., 2023 [91] | Yes | NA | Yes | Yes | Yes | Unclear | Yes | Yes | Unclear | Yes | Yes | Yes | Yes | No | Yes | Yes | Yes | Yes | **82%** | **Low risk** |
| Cowie et al., 2018 [36] | Yes | NA | Yes | Yes | Unclear | Unclear | Yes | Yes | Unclear | Yes | Yes | Yes | Yes | No | No | Yes | Yes | Yes | **71%** | **Moderate risk** |
| Hammerton et al., 2022 [50] | Yes | NA | Yes | Yes | Unclear | Unclear | Yes | Yes | Unclear | Yes | Yes | Yes | Yes | No | No | Yes | Yes | Yes | **71%** | **Moderate risk** |
| Johansson et al., 2020 [67] | Yes | NA | Yes | Yes | Unclear | Unclear | Yes | Yes | Unclear | Yes | Yes | Yes | Yes | No | No | Yes | Unclear | Yes | **65%** | **Moderate risk** |
| Klocek et al., 2019 [41] | Yes | NA | Yes | Yes | Yes | Yes | Yes | Yes | Unclear | Yes | Yes | Yes | Yes | No | No | Yes | Yes | Yes | **82%** | **Low risk** |
| Knop et al., 2021 [47] | Yes | NA | Yes | Yes | Yes | Unclear | Yes | Yes | Unclear | Yes | Yes | Yes | Yes | No | No | Yes | Yes | Yes | **76%** | **Moderate risk** |
| Koch and Guhres, 2020 [68] | Yes | NA | Yes | Unclear | Unclear | Unclear | Yes | Yes | Unclear | Yes | Yes | Yes | Yes | No | No | Yes | Unclear | Yes | **59%** | **Moderate risk** |
| Kriegel et al., 2017 [35] | Unclear | NA | Yes | Unclear | Unclear | No | Yes | Yes | Unclear | Yes | Unclear | Unclear | Unclear | No | No | Yes | Unclear | Yes | **35%** | **High risk** |
| Peeters et al., 2016 [34] | Yes | NA | Yes | Yes | Unclear | Unclear | Yes | Yes | Unclear | Yes | Yes | Yes | Yes | No | No | Yes | Unclear | Yes | **65%** | **Moderate risk** |
| Poss-Doering et al., 2020 [43] | Yes | NA | Yes | Yes | Yes | Yes | Yes | Yes | Yes | Yes | Yes | Yes | Yes | Yes | Yes | Yes | Yes | Yes | **100%** | **Low risk** |
| Potthoff et al., 2024 [62] | Yes | NA | Yes | Yes | Yes | Yes | Yes | Yes | Yes | Yes | Yes | Yes | Yes | No | No | Yes | Yes | Yes | **88%** | **Low risk** |
| Preiser et al., 2024 [63] | Unclear | NA | Yes | Yes | Unclear | Unclear | Yes | Yes | Yes | Yes | Yes | Yes | Yes | Yes | Yes | Yes | Yes | Yes | **82%** | **Low risk** |
| Schendzielorz et al., 2023 [64] | Yes | NA | Yes | Yes | Unclear | Unclear | Yes | Yes | Unclear | Yes | Yes | Yes | Yes | No | No | Yes | Yes | Yes | **71%** | **Moderate risk** |
| Tensen et al., 2023 [38] | Yes | NA | Yes | Yes | Unclear | Unclear | Yes | Yes | Unclear | Yes | Yes | Yes | Yes | No | No | Yes | Yes | Yes | **71%** | **Moderate risk** |
| Van De Vijver et al., 2022 [51] | Yes | NA | Yes | Yes | Yes | Yes | Yes | Yes | Unclear | Yes | Yes | Yes | Yes | No | No | Yes | Yes | Yes | **82%** | **Low risk** |
| Wangler & Jansky, 2024 [65] | Yes | NA | Yes | Yes | Yes | Unclear | Yes | Yes | Unclear | Yes | Yes | Yes | Yes | No | No | Yes | Unclear | Yes | **71%** | **Moderate risk** |
| Weik et al., 2024 [66] | Yes | NA | Yes | Yes | Yes | Yes | Yes | Yes | Yes | Yes | Yes | Yes | Yes | Yes | Yes | Yes | Yes | Yes | **100%** | **Low risk** |

**Abbreviations**

GP: General practitioner

JBI: Joanna Briggs Institute

MMAT: Mixed Methods Appraisal Tool

NPT: Normalization Process Theory

RCT: Randomized controlled trial

TAM: Technology Acceptance Model

**References**

29. Albrecht UV, Afshar K, Illiger K, et al. Expectancy, usage and acceptance by general practitioners and patients: exploratory results from a study in the German outpatient sector. Digit Health. 2017;3:2055207617695135. [doi: 10. 1177/2055207617695135] [Medline: 29942582]

71. Hvidt EA, Grønning A, Brøgger MN, Møller JE, Fage-Butler A. Multilevel structures and human agency in relation to email consultations: a strong structuration theory analysis of the Danish general practice setting. Soc Sci Med. Aug 2021;282:114155. [doi: 10.1016/j.socscimed.2021.114155] [Medline: 34174578]

33. Blease C, Kaptchuk TJ, Bernstein MH, Mandl KD, Halamka JD, DesRoches CM. Artificial intelligence and the future of primary care: exploratory qualitative study of UK general practitioners’ views. J Med Internet Res. Mar 20, 2019;21(3):e12802. [doi: 10.2196/12802] [Medline: 30892270]

91. Blease C, Torous J, Dong Z, et al. Patient online record access in English primary care: qualitative survey study of general practitioners’ views. J Med Internet Res. 2023;25:e43496. [doi: 10.2196/43496] [Medline: 36811939]

89. Blease CR, Locher C, Gaab J, Hägglund M, Mandl KD. Generative artificial intelligence in primary care: an online survey of UK general practitioners. BMJ Health Care Inform. Aug 2024;31(1):e101102. [doi: 10.1136/bmjhci-2024- 101102] [Medline: 39288998]

49. Bondaronek P, Dicken SJ, Singh Jennings S, Mallion V, Stefanidou C. Barriers to and facilitators of the use of digital tools in primary care to deliver physical activity advice: semistructured interviews and thematic analysis. JMIR Hum Factors. Aug 30, 2022;9(3):e35070. [doi: 10.2196/35070] [Medline: 36040764]

56. Brecher AL, Herrgen J, Mueller T. DiGA in primary care and the influence of patient-related factors - results of a survey of family doctors. Hayn D, Pfeifer B, Schreier G, Baumgartner M, editors. Studies in Health Technology and Informatics. Apr 26, 2024;313:55-61. [doi: 10.3233/SHTI240012] [Medline: 38682505]

39. Breedvelt JJ, Zamperoni V, Kessler D, et al. GPs’ attitudes towards digital technologies for depression: an online survey in primary care. Br J Gen Pract. Mar 2019;69(680):e164-e170. [doi: 10.3399/bjgp18X700721] [Medline: 30559111]

84. Buck C, Doctor E, Hennrich J, Jöhnk J, Eymann T. General practitioners’ attitudes toward artificial intelligence-enabled systems: interview study. J Med Internet Res. Jan 27, 2022;24(1):e28916. [doi: 10.2196/28916] [Medline: 35084342]

40. Buhtz C, Paulicke D, Schwarz K, Jahn P, Stoevesandt D, Frese T. Receptiveness of GPs in the south of Saxony-Anhalt, Germany to obtaining training on technical assistance systems for caregiving: a cross-sectional study. Clin Interv Aging. 2019;14:1649-1656. [doi: 10.2147/CIA.S218367] [Medline: 31571844]

52. Butler K, Bartlett YK, Newhouse N, et al. Implementing a text message-based intervention to support type 2 diabetes medication adherence in primary care: a qualitative study with general practice staff. BMC Health Serv Res. Jun 10, 2023;23(1):614-623. [doi: 10.1186/s12913-023-09571-9] [Medline: 37301867]

90. Cabitza F, Misericordia P, Del Zotti F. Electronic records for general practice - where we are, where we should head to improve them. Presented at: International Conference on Health Informatics; Mar 3-4, 2014:535-542; ESEO, Angers, Loire Valley, France. [doi: 10.5220/0004924805350542]

36. Cowie J, Calveley E, Bowers G, Bowers J. Evaluation of a digital consultation and self-care advice tool in primary care: a multi-methods study. Int J Environ Res Public Health. May 2, 2018;15(5):896-918. [doi: 10.3390/ijerph15050896] [Medline: 29724040]

92. Davidge G, Brown L, Lyons M, et al. Primary care staff’s views and experience of patients’ online access to their electronic health record: a qualitative exploration. Br J Gen Pract. Jun 2023;73(731):e418-e426. [doi: 10.3399/BJGP. 2022.0436] [Medline: 37068967]

42. Entezarjou A, Bolmsjö BB, Calling S, Midlöv P, Milos Nymberg V. Experiences of digital communication with automated patient interviews and asynchronous chat in Swedish primary care: a qualitative study. BMJ Open. Jul 23, 2020;10(7):e036585. [doi: 10.1136/bmjopen-2019-036585] [Medline: 32709650]

31. Fagerlund AJ, Holm IM, Zanaboni P. General practitioners’ perceptions towards the use of digital health services for citizens in primary care: a qualitative interview study. BMJ Open. May 5, 2019;9(5):e028251. [doi: 10.1136/bmjopen2018-028251] [Medline: 31061056]

37. Gabriels K, Moerenhout T. Exploring entertainment medicine and professionalization of self-care: interview study among doctors on the potential effects of digital self-tracking. J Med Internet Res. Jan 12, 2018;20(1):e10. [doi: 10. 2196/jmir.8040] [Medline: 29330140]

57. Gavin JP, Clarkson P, Muckelt PE, et al. Healthcare professional and commissioners’ perspectives on the factors facilitating and hindering the implementation of digital tools for self-management of long-term conditions within UK healthcare pathways. PLOS ONE. 2024;19(8):e0307493. [doi: 10.1371/journal.pone.0307493] [Medline: 39178238]

58. Ghis Malfilatre M, Louvel S. A medical profession towards empowerment? The uncertain future of digitalization in private general practice. Soc Sci Med. Jan 2025;365:117575. [doi: 10.1016/j.socscimed.2024.117575] [Medline: 39673876]

44. Glock H, Milos Nymberg V, Borgström Bolmsjö B, et al. Attitudes, barriers, and concerns regarding telemedicine among swedish primary care physicians: a qualitative study. IJGM. 2021;14:9237-9246. [doi: 10.2147/IJGM.S334782] [Medline: 34880663]

83. Grønning A, Simonsen LM, Lüchau EC, Hvidt EA, Klausen M. My time, your time, our time. Older patients’ and GPs’ time sensibilities around email consultations. Health Sociol Rev. Mar 2024;33(1):43-58. [doi: 10.1080/14461242.2024. 2316742] [Medline: 38385438]

50. Hammerton M, Benson T, Sibley A. Readiness for five digital technologies in general practice: perceptions of staff in one part of southern England. BMJ Open Qual. Jun 2022;11(2):e001865. [doi: 10.1136/bmjoq-2022-001865] [Medline: 35768171]

45. Jakobsen PR, Christensen JR, Nielsen JB, et al. Identification of important factors affecting use of digital individualised coaching and treatment of type 2 diabetes in general practice: a qualitative feasibility study. Int J Environ Res Public Health. Apr 8, 2021;18(8):3924. [doi: 10.3390/ijerph18083924] [Medline: 33917999]

67. Johansson A, Larsson M, Ivarsson B. General practitioners’ experiences of digital written patient dialogues: a pilot study using a mixed method. J Prim Care Community Health. 2020;11:2150132720909656. [doi: 10.1177/ 2150132720909656] [Medline: 32133905]

72. Johnsen TM, Norberg BL, Kristiansen E, et al. Suitability of video consultations during the COVID-19 pandemic lockdown: cross-sectional survey among Norwegian general practitioners. J Med Internet Res. Feb 8, 2021;23(2):e26433. [doi: 10.2196/26433] [Medline: 33465037]

46. Keuper J, Batenburg R, Verheij R, van Tuyl L. Use of e-Health in Dutch general practice during the COVID-19 pandemic. Int J Environ Res Public Health. Nov 26, 2021;18(23):12479. [doi: 10.3390/ijerph182312479] [Medline: 34886204]

79. Klausen M, Hvidt EA. E‐consultation as existential media: exploring doctor‐patient ‘digital thrownness’ in Danish general practice. Sociol Health Illness. Nov 2024;46(8):1849-1863. [doi: 10.1111/1467-9566.13823]

41. Klocek A, Šmahelová M, Knapová L, Elavsky S. GPs’ perspectives on eHealth use in the Czech Republic: a cross-sectional mixed-design survey study. BJGP Open. Oct 2019;3(3):bjgpopen19X101655. [doi: 10.3399/ bjgpopen19X101655] [Medline: 31344683]

47. Knop M, Mueller M, Niehaves B. Investigating the use of telemedicine for digitally mediated delegation in team-based primary care: mixed methods study. J Med Internet Res. Aug 26, 2021;23(8):e28151. [doi: 10.2196/28151] [Medline: 34435959]

68. Koch S, Guhres M. Physicians’ Experiences of patient-initiated online consultations in primary care using direct-to-consumer technology. Studies in Health Technology and Informatics. Jun 16, 2020;270:643-647. [doi: 10.3233/ SHTI200239] [Medline: 32570462]

80. Kofod FG, Hvidt EA, Arreskov AB, Guassora AD. Interpersonal contact and altered sensory conditions in video consultation – a qualitative interview study in Danish general practice. Scand J Prim Health Care. Oct 2024;42(4):650-658. [doi: 10.1080/02813432.2024.2376744] [Medline: 39012270]

81. Kollmann J, Sana S, Magnée T, et al. Patients’ and professionals’ experiences with remote care during COVID-19: a qualitative study in general practices in low-income neighborhoods. Prim Health Care Res Dev. Jun 3, 2024;25:e32. [doi: 10.1017/S1463423624000240] [Medline: 38826073]

35. Kriegel J, Tuttle-Weidinger L, Reckwitz L. Digital media for primary health care in Austria. Stud Health Technol Inform. 2017;236:282-289. [doi: 10.3233/978-1-61499-759-7-282] [Medline: 28508808]

53. Kujansivu K, Tolvanen E, Kautto M, Koskela TH. The use of digital tools by general practitioners in Finnish public health centres. Finn J EHealth EWelfare. 2023;15(1):40-51. [doi: 10.23996/fjhw.122703]

76. Lüchau EC, Atherton H, Olesen F, Søndergaard J, Hvidt EA. Interpreting technology: use and non-use of doctor-patient video consultations in Danish general practice. Soc Sci Med. Oct 2023;334:116215. [doi: 10.1016/j.socscimed.2023. 116215] [Medline: 37690154]

86. Mahlknecht A, Engl A, Piccoliori G, Wiedermann CJ. Supporting primary care through symptom checking artificial intelligence: a study of patient and physician attitudes in Italian general practice. BMC Prim Care. Sep 4, 2023;24(1):174. [doi: 10.1186/s12875-023-02143-0] [Medline: 37661285]

73. Meurs M, Keuper J, Sankatsing V, Batenburg R, van Tuyl L. “Get used to the fact that some of the care is really going to take place in a different way”: general practitioners’ experiences with e-Health during the COVID-19 pandemic. Int J Environ Res Public Health. Apr 22, 2022;19(9):5120. [doi: 10.3390/ijerph19095120] [Medline: 35564519]

30. Morrissey EC, Glynn LG, Casey M, Walsh JC, Molloy GJ. New self-management technologies for the treatment of hypertension: general practitioners’ perspectives. Fam Pract. May 23, 2018;35(3):318-322. [doi: 10.1093/fampra/ cmx100] [Medline: 29088438]

69. Müller M, Knop M, Ressing C. Constituting factors of a digitally influenced relationship between patients and primary care physicians in rural areas. Presented at: Hawaii International Conference on System Sciences; Jan 7-10, 2020; Grand Wailea Maui, Hawaii. [doi: 10.24251/HICSS.2020.447] 67. Waschkau A, Flägel K, Goetz K, Steinhäuser J. Evaluation

59. Nassehi D, Ramvi E. Care in the era of digital health: experiences from Norwegian general practitioners. Scand J Prim Health Care. Jun 2025;43(2):338-346. [doi: 10.1080/02813432.2024.2437417] [Medline: 39639473]

60. Newbould J, Hocking L, Sidhu M, Daniel K. Digital First Primary Care for those with multiple long-term conditions: a rapid review of the views of stakeholders. Health Soc Care Deliv Res. Jul 2024;12(21):1-68. [doi: 10.3310/AWBT4827] [Medline: 39056123]

32. Norberg BL, Austad B, Kristiansen E, Zanaboni P, Getz LO. The impact and wider implications of remote consultations for general practice in Norway: qualitative study among Norwegian contract general practitioners. JMIR Form Res. Dec 17, 2024;8:e63068. [doi: 10.2196/63068] [Medline: 39688890]

34. Peeters JM, Krijgsman JW, Brabers AE, Jong JDD, Friele RD. Use and uptake of eHealth in general practice: a crosssectional survey and focus group study among health care users and general practitioners. JMIR Med Inf. Apr 6, 2016;4(2):e11. [doi: 10.2196/medinform.4515] [Medline: 27052805]

77. Peeters K, Giroldi E, Veldhuizen L, Abdelaziz K, Muris D, Cals J. General practitioner use of e-consultation to consult hospital specialists: interview study to obtain physician’s perceptions about digital interprofessional communication. J Med Internet Res. 2023;25:e40318. [doi: 10.2196/40318] [Medline: 36976626]

48. Pikkemaat M, Thulesius H, Milos Nymberg V. Swedish primary care physicians’ intentions to use telemedicine: a survey using a new questionnaire - physician attitudes and intentions to use telemedicine (PAIT). Int J Gen Med. 2021;14:3445-3455. [doi: 10.2147/IJGM.S319497] [Medline: 34295177]

43. Poss-Doering R, Kuehn L, Kamradt M, Glassen K, Wensing M. Applying digital information delivery to convert habits of antibiotic use in primary care in Germany: mixed-methods study. J Med Internet Res. Oct 7, 2020;22(10):e18200. [doi: 10.2196/18200] [Medline: 32960773]

61. Posselt J, Lander J, Dierks ML. Digitale gesundheitsanwendungen in der hausärztlichen versorgung: eine diskussionsgrundlage zur förderung informierter nutzungsentscheidungen [Article in German]. Präv Gesundheitsf. Nov 2024;19(4):483-489. [doi: 10.1007/s11553-024-01126-y]

62. Potthoff S, Brendryen H, Bosnic H, Njå ALM, Finch T, Lid TG. Recommendations for implementing digital alcohol interventions in primary care: lessons learned from a Norwegian feasibility study. Front Health Serv. 2024;4:1343568. [doi: 10.3389/frhs.2024.1343568] [Medline: 39469438]

63. Preiser C, Radionova N, Ög E, et al. The doctors, their patients, and the symptom checker app: qualitative interview study with general practitioners in Germany. JMIR Hum Factors. Nov 18, 2024;11:e57360. [doi: 10.2196/57360] [Medline: 39556813]

64. Schendzielorz J, Harre K, Tarara M, Oess S, Holmberg C. Digitale technologien in der allgemeinmedizin: aktueller stand und zukünftige anforderungen [Article in German]. Z Für Allg. 2023;100(1):21-29. [doi: 10.1007/s44266-023- 00145-w]

87. Schütze D, Holtz S, Neff MC, et al. Requirements analysis for an AI-based clinical decision support system for general practitioners: a user-centered design process. BMC Med Inform Decis Mak. Jul 31, 2023;23(1):144. [doi: 10.1186/ s12911-023-02245-w] [Medline: 37525175]

54. Solberg Carlsson K, Øvretveit J, Ohrling M. Rapid implementation of remote digital primary care in Stockholm and implications for further system-wide implementation: practitioner’s and manager’s experience of the Always Open mobile application. Scand J Prim Health Care. Sep 2023;41(3):232-246. [doi: 10.1080/02813432.2023.2229387] [Medline: 37470469]

82. Stamer T, Traulsen P, Rieken J, Schmahl T, Menrath I, Steinhäuser J. Determinants of the implementation of eHealthbased long-term follow-up care for young cancer survivors: a qualitative study. BMC Cancer. Sep 18, 2024;24(1):1159. [doi: 10.1186/s12885-024-12910-6] [Medline: 39294671]

85. Tabla S, Calafiore M, Legrand B, et al. Artificial intelligence and clinical decision support systems or automated interpreters: what characteristics are expected by French general practitioners? Otero P, Scott P, Martin SZ, Huesing E, editors. Stud Health Technol Inform. Jun 6, 2022;290:887-891. [doi: 10.3233/SHTI220207] [Medline: 35673146]

78. Tensen E, Kuziemsky C, Jaspers MW, Peute LW. General practitioners’ perspectives about remote dermatology care during the COVID-19 pandemic in the Netherlands: questionnaire-based study. JMIR Dermatol. Jun 13, 2023;6:e46682. [doi: 10.2196/46682] [Medline: 37632975]

38. Torrent-Sellens J, Díaz-Chao Á, Soler-Ramos I, Saigí-Rubió F. Modeling and predicting outcomes of eHealth usage by European physicians: multidimensional approach from a survey of 9196 general practitioners. J Med Internet Res. Oct 22, 2018;20(10):e279. [doi: 10.2196/jmir.9253] [Medline: 30348628]

74. Turner A, Morris R, Rakhra D, et al. Unintended consequences of online consultations: a qualitative study in UK primary care. Br J Gen Pract. Feb 2022;72(715):e128-e137. [doi: 10.3399/BJGP.2021.0426] [Medline: 34903520]

93. Turner A, Morris R, McDonagh L, et al. Unintended consequences of patient online access to health records: a qualitative study in UK primary care. Br J Gen Pract. Jan 2023;73(726):e67-e74. [doi: 10.3399/BJGP.2021.0720] [Medline: 36316163]

51. van de Vijver S, Hummel D, van Dijk AH, et al. Evaluation of a digital self-management platform for patients with chronic illness in primary care: qualitative study of stakeholders’ perspectives. JMIR Form Res. Aug 3, 2022;6(8):e38424. [doi: 10.2196/38424] [Medline: 35921145]

55. Wangler J, Jansky M. Two years of approved digital health applications in Germany - perspectives and experiences of general practitioners with an affinity for their use. Eur J Gen Pract. Dec 2023;29(1):2186396. [doi: 10.1080/13814788. 2023.2186396] [Medline: 36919621]

65. Wangler J, Jansky M. How can primary care benefit from digital health applications? – A quantitative, explorative survey on attitudes and experiences of general practitioners in Germany. BMC Digit Health. 2024;2(1):14. [doi: 10. 1186/s44247-024-00068-x]

70. Waschkau A, Flägel K, Goetz K, Steinhäuser J. Evaluation of attitudes towards telemedicine as a basis for successful implementation: a cross-sectional survey among postgraduate trainees in family medicine in Germany. Z Evid Fortbild Qual Gesundhwes. Nov 2020;156:75-81. [doi: 10.1016/j.zefq.2020.07.001] [Medline: 32859557]

75. Waschkau A, Traulsen P, Steinhäuser J. Evaluation of synchronous and asynchronous telemedical applications in primary care in rural regions of northern Germany-results and lessons learned from a pilot study. Int J Environ Res Public Health. Nov 11, 2022;19(22):14860. [doi: 10.3390/ijerph192214860] [Medline: 36429577]

66. Weik L, Fehring L, Mortsiefer A, Meister S. Understanding inherent influencing factors to digital health adoption in general practices through a mixed-methods analysis. NPJ Digit Med. Feb 27, 2024;7(1):47. [doi: 10.1038/s41746-024- 01049-0] [Medline: 38413767]

88. Wewetzer L, Held LA, Goetz K, Steinhäuser J. Determinants of the implementation of artificial intelligence-based screening for diabetic retinopathy-a cross-sectional study with general practitioners in Germany. Digit Health. 2023;9:20552076231176644. [doi: 10.1177/20552076231176644] [Medline: 37274367]
