# Supplementary material for: The Impact of Digitalization on European General Practice From the Perspective of General Practitioners: Systematic Review
Source: J Med Internet Res. 2026 Aug 18;28:e83343. doi: 10.2196/83343 (PMC13485116; doi:10.2196/83343)
Supplement: Supplementary file 6 [file jmir-v28-e83343-s007.docx]

**TAM-NPT Full Percentage Breakdown and Sensitivity Analysis**

*Complete response distributions by technology type*

**Table S1.** Technology Acceptance Model (TAM) analysis.

Percentage of studies reporting each response category by TAM construct and technology type.

| **Construct / Response** | **YES** | **PARTIAL** | **UNCLEAR** | **NO** |
| --- | --- | --- | --- | --- |
| **Perceived Usefulness** | | | | |
| AI/CDSS (n=7) | 85.7% | 14.3% | 0.0% | 0.0% |
| DHT (n=36) | 83.3% | 13.9% | 2.8% | 0.0% |
| MR (n=4) | 100.0% | 0.0% | 0.0% | 0.0% |
| TM (n=18) | 88.9% | 11.1% | 0.0% | 0.0% |
| Overall (n=65) | 86.2% | 12.3% | 1.5% | 0.0% |
| **Perceived Ease of Use** | | | | |
| AI/CDSS (n=7) | 0.0% | 42.9% | 0.0% | 57.1% |
| DHT (n=36) | 0.0% | 11.1% | 13.9% | 75.0% |
| MR (n=4) | 0.0% | 25.0% | 25.0% | 50.0% |
| TM (n=18) | 0.0% | 27.8% | 16.7% | 55.6% |
| Overall (n=65) | 0.0% | 20.0% | 13.8% | 66.2% |
| **Behavioral Intention** | | | | |
| AI/CDSS (n=7) | 0.0% | 85.7% | 0.0% | 14.3% |
| DHT (n=36) | 2.8% | 88.9% | 0.0% | 8.3% |
| MR (n=4) | 0.0% | 100.0% | 0.0% | 0.0% |
| TM (n=18) | 11.1% | 72.2% | 0.0% | 16.7% |
| Overall (n=65) | 4.6% | 84.6% | 0.0% | 10.8% |
| **Actual Use** | | | | |
| AI/CDSS (n=7) | 100.0% | 0.0% | 0.0% | 0.0% |
| DHT (n=36) | 94.4% | 5.6% | 0.0% | 0.0% |
| MR (n=4) | 100.0% | 0.0% | 0.0% | 0.0% |
| TM (n=18) | 100.0% | 0.0% | 0.0% | 0.0% |
| Overall (n=65) | 96.9% | 3.1% | 0.0% | 0.0% |

**Table S2.** Normalization Process Theory (NPT) analysis.

Percentage of studies reporting each response category by NPT construct and technology type.

| **Construct / Response** | **YES** | **PARTIAL** | **UNCLEAR** | **NO** |
| --- | --- | --- | --- | --- |
| **Coherence** | | | | |
| AI/CDSS (n=7) | 28.6% | 28.6% | 42.9% | 0.0% |
| DHT (n=36) | 52.8% | 16.7% | 22.2% | 8.3% |
| MR (n=4) | 75.0% | 0.0% | 25.0% | 0.0% |
| TM (n=18) | 50.0% | 0.0% | 50.0% | 0.0% |
| Overall (n=65) | 50.8% | 12.3% | 32.3% | 4.6% |
| **Cognitive Participation** | | | | |
| AI/CDSS (n=7) | 0.0% | 28.6% | 0.0% | 71.4% |
| DHT (n=36) | 2.8% | 58.3% | 11.1% | 27.8% |
| MR (n=4) | 25.0% | 50.0% | 0.0% | 25.0% |
| TM (n=18) | 5.6% | 33.3% | 27.8% | 33.3% |
| Overall (n=65) | 4.6% | 47.7% | 13.8% | 33.9% |
| **Collective Action** | | | | |
| AI/CDSS (n=7) | 0.0% | 100.0% | 0.0% | 0.0% |
| DHT (n=36) | 0.0% | 100.0% | 0.0% | 0.0% |
| MR (n=4) | 0.0% | 100.0% | 0.0% | 0.0% |
| TM (n=18) | 0.0% | 100.0% | 0.0% | 0.0% |
| Overall (n=65) | 0.0% | 100.0% | 0.0% | 0.0% |
| **Reflexive Monitoring** | | | | |
| AI/CDSS (n=7) | 71.4% | 28.6% | 0.0% | 0.0% |
| DHT (n=36) | 63.9% | 36.1% | 0.0% | 0.0% |
| MR (n=4) | 100.0% | 0.0% | 0.0% | 0.0% |
| TM (n=18) | 61.1% | 38.9% | 0.0% | 0.0% |
| Overall (n=65) | 66.2% | 33.9% | 0.0% | 0.0% |

**Notes:**

• YES = Construct clearly fulfilled/positive direction

• PARTIAL = Construct partially fulfilled/mixed findings

• UNCLEAR = Insufficient information to determine

• NO = Construct not fulfilled/negative direction

Abbreviations: AI/CDSS = Artificial intelligence/clinical decision support systems; DHT = Digital health tools; MR = Medical records; TM = Telemedicine; UK = United Kingdom.

**Table S3.** Geographic distribution by technology type.

Country-level stratification of included studies by technology category.

| **Technology Type** | **Countries (n)** | **Country Distribution** |
| --- | --- | --- |
| AI/CDSS (n=7) | 4 | Germany (42.9%), UK (28.6%), France (14.3%), Italy (14.3%) |
| DHT (n=36) | 13 | Germany (30.6%), UK (19.4%), Sweden (11.1%), Netherlands (8.3%), Norway (8.3%), Austria (2.8%), Belgium (2.8%), Czech Republic (2.8%), Denmark (2.8%), Finland (2.8%), France (2.8%), Ireland (2.8%), Spain (Multi-country) (2.8%) |
| MR (n=4) | 2 | UK (75.0%), Italy (25.0%) |
| TM (n=18) | 6 | Denmark (27.8%), Germany (22.2%), Netherlands (22.2%), Sweden (11.1%), Norway (11.1%), UK (5.6%) |
| Overall (n=65) | 13 | Germany (27.7%), UK (20%), Netherlands (10.8%), Denmark (9.2%), Sweden (9.2%), Norway (7.7%), France (3.1%), Italy (3.1%), Austria (1.5%), Belgium (1.5%), Czech Republic (1.5%), Finland (1.5%), Ireland (1.5%), Spain (Multi-country) (1.5%) |

**Table S4.** Technology Acceptance Model (TAM) constructs by country.

**Note:** Single-country studies are presented for each country. The Torrent-Sellens et al. (2018) [38] multi-country study is listed separately at the end.
**Abbreviations:** PAR = Partially fulfilled; UNC = Unclear/insufficient information; AI = Artificial intelligence/clinical decision support systems; DHT = Digital health tools; MR = Medical records; TM = Telemedicine; n = number of studies.
**Interpretation:** YES = Construct clearly fulfilled; PARTIAL = Mixed or partially fulfilled; UNCLEAR = Insufficient information to determine; NO = Construct not fulfilled
**Caveat:** Country‑level percentages and effect‑direction arrows should be interpreted as illustrative rather than definitive. Several countries are represented by only one or two studies, sometimes older, and different countries contributed data on different types of technologies (e.g., patient portals vs. wearables or telemonitoring systems). Apparent differences between countries may therefore reflect variation in study period, sample size, and technology mix at least as much as true cross‑country contrasts.

| **Country** | **n** | **Technologies** | | | | **Perceived Usefulness (%)** | | | | | **Perceived Ease of Use (%)** | | | | | **Behavioral Intention (%)** | | | | | **Actual Use (%)** | | | | |
| --- | --- | --- | --- | --- | --- | --- | --- | --- | --- | --- | --- | --- | --- | --- | --- | --- | --- | --- | --- | --- | --- | --- | --- | --- | --- |
|  |  | **DHT** | **MR** | **AI** | **TM** | **YES** | **PAR** | **UNC** | **NO** | **Effect Direction** | **YES** | **PAR** | **UNC** | **NO** | **Effect Direction** | **YES** | **PAR** | **UNC** | **NO** | **Effect Direction** | **YES** | **PAR** | **UNC** | **NO** | **Effect Direction** |
| **Germany** | 18 | 11 | 0 | 3 | 4 | 83.3 | 11.1 | 5.6 | 0.0 | ⮅ | 0.0 | 22.2 | 22.2 | 55.6 | ⭣ | 0.0 | 88.9 | 0.0 | 11.1 | ⭤ | 94.4 | 5.6 | 0.0 | 0.0 | ⮅ |
| **UK** | 13 | 7 | 3 | 2 | 1 | 92.3 | 7.7 | 0.0 | 0.0 | ⮅ | 0.0 | 30.8 | 0.0 | 69.2 | ⮇ | 0.0 | 92.3 | 0.0 | 7.7 | ⭤ | 100.0 | 0.0 | 0.0 | 0.0 | ⮅ |
| **Netherlands** | 7 | 3 | 0 | 0 | 4 | 85.7 | 14.3 | 0.0 | 0.0 | ⮅ | 0.0 | 14.3 | 14.3 | 71.4 | ⮇ | 42.9 | 42.9 | 0.0 | 14.3 | ⭤ | 100.0 | 0.0 | 0.0 | 0.0 | ⮅ |
| **Sweden** | 6 | 4 | 0 | 0 | 2 | 83.3 | 16.7 | 0.0 | 0.0 | ⮅ | 0.0 | 0.0 | 16.7 | 83.3 | ⮇ | 0.0 | 100.0 | 0.0 | 0.0 | ⭤ | 83.3 | 16.7 | 0.0 | 0.0 | ⮅ |
| **Denmark** | 6 | 1 | 0 | 0 | 5 | 100.0 | 0.0 | 0.0 | 0.0 | ⮅ | 0.0 | 33.3 | 0.0 | 66.7 | ⮇ | 0.0 | 66.7 | 0.0 | 33.3 | ⭣ | 100.0 | 0.0 | 0.0 | 0.0 | ⮅ |
| **Norway** | 5 | 3 | 0 | 0 | 2 | 100.0 | 0.0 | 0.0 | 0.0 | ⮅ | 0.0 | 20.0 | 20.0 | 60.0 | ⭣ | 0.0 | 100.0 | 0.0 | 0.0 | ⭤ | 100.0 | 0.0 | 0.0 | 0.0 | ⮅ |
| **France** | 2 | 1 | 0 | 1 | 0 | 50.0 | 50.0 | 0.0 | 0.0 | ⭤ | 0.0 | 0.0 | 0.0 | 100.0 | ⮇ | 0.0 | 50.0 | 0.0 | 50.0 | ⭣ | 100.0 | 0.0 | 0.0 | 0.0 | ⮅ |
| **Italy** | 2 | 0 | 1 | 1 | 0 | 100.0 | 0.0 | 0.0 | 0.0 | ⮅ | 0.0 | 0.0 | 50.0 | 50.0 | ⭣ | 0.0 | 100.0 | 0.0 | 0.0 | ⭤ | 100.0 | 0.0 | 0.0 | 0.0 | ⮅ |
| **Austria** | 1 | 1 | 0 | 0 | 0 | 100.0 | 0.0 | 0.0 | 0.0 | ⮅ | 0.0 | 0.0 | 0.0 | 100.0 | ⮇ | 0.0 | 100.0 | 0.0 | 0.0 | ⭤ | 100.0 | 0.0 | 0.0 | 0.0 | ⮅ |
| **Finland** | 1 | 1 | 0 | 0 | 0 | 100.0 | 0.0 | 0.0 | 0.0 | ⮅ | 0.0 | 0.0 | 0.0 | 100.0 | ⮇ | 0.0 | 100.0 | 0.0 | 0.0 | ⭤ | 100.0 | 0.0 | 0.0 | 0.0 | ⮅ |
| **Czech Republic** | 1 | 1 | 0 | 0 | 0 | 100.0 | 0.0 | 0.0 | 0.0 | ⮅ | 0.0 | 0.0 | 0.0 | 100.0 | ⮇ | 0.0 | 100.0 | 0.0 | 0.0 | ⭤ | 100.0 | 0.0 | 0.0 | 0.0 | ⮅ |
| **Belgium** | 1 | 1 | 0 | 0 | 0 | 0.0 | 100.0 | 0.0 | 0.0 | ⭤ | 0.0 | 0.0 | 100.0 | 0.0 | ⭤ | 0.0 | 100.0 | 0.0 | 0.0 | ⭤ | 100.0 | 0.0 | 0.0 | 0.0 | ⮅ |
| **Ireland** | 1 | 1 | 0 | 0 | 0 | 100.0 | 0.0 | 0.0 | 0.0 | ⮅ | 0.0 | 0.0 | 0.0 | 100.0 | ⮇ | 0.0 | 100.0 | 0.0 | 0.0 | ⭤ | 100.0 | 0.0 | 0.0 | 0.0 | ⮅ |
| **Multi-country (Torrent-Sellens et al. 2018 [38])** | 1 | 1 | 0 | 0 | 0 | 0.0 | 100.0 | 0.0 | 0.0 | ⭤ | 0.0 | 100.0 | 0.0 | 0.0 | ⭤ | 0.0 | 100.0 | 0.0 | 0.0 | ⭤ | 100.0 | 0.0 | 0.0 | 0.0 | ⮅ |

**Table S5.** Normalization Process Theory (NPT) constructs by country.

**Note:** Single-country studies are presented for each country. The Torrent-Sellens et al. (2018) [38] multi-country study is listed separately at the end and not counted toward any single country.
**Abbreviations:** COH = Coherence; CP = Cognitive Participation; CA = Collective Action; RM = Reflexive Monitoring; PAR = Partially fulfilled; UNC = Unclear/insufficient information; AI = Artificial intelligence/clinical decision support systems; DHT = Digital health tools; MR = Medical records; TM = Telemedicine; n = number of studies.
**Interpretation:** YES = Construct clearly fulfilled; PARTIAL = Mixed or partially fulfilled; UNCLEAR = Insufficient information to determine; NO = Construct not fulfilled
**Caveat:** Country‑level percentages and effect‑direction arrows should be interpreted as illustrative rather than definitive. Several countries are represented by only one or two studies, sometimes older, and different countries contributed data on different types of technologies (e.g., patient portals vs. wearables or telemonitoring systems). Apparent differences between countries may therefore reflect variation in study period, sample size, and technology mix at least as much as true cross‑country contrasts.

| **Country** | **n** | **Technologies** | | | | **Coherence (%)** | | | | | **Cognitive Participation (%)** | | | | | **Collective Action (%)** | | | | | **Reflexive Monitoring (%)** | | | | |
| --- | --- | --- | --- | --- | --- | --- | --- | --- | --- | --- | --- | --- | --- | --- | --- | --- | --- | --- | --- | --- | --- | --- | --- | --- | --- |
|  |  | **DHT** | **MR** | **AI** | **TM** | **YES** | **PAR** | **UNC** | **NO** | **Effect Direction** | **YES** | **PAR** | **UNC** | **NO** | **Effect Direction** | **YES** | **PAR** | **UNC** | **NO** | **Effect Direction** | **YES** | **PAR** | **UNC** | **NO** | **Effect Direction** |
| **Germany** | 18 | 11 | 0 | 3 | 4 | 44.4 | 16.7 | 33.3 | 5.6 | ⭤ | 5.6 | 44.4 | 16.7 | 33.3 | ⮇ | 0.0 | 100.0 | 0.0 | 0.0 | ⭤ | 72.2 | 27.8 | 0.0 | 0.0 | ⭤ |
| **UK** | 13 | 7 | 3 | 2 | 1 | 61.5 | 30.8 | 7.7 | 0.0 | ⭤ | 0.0 | 69.2 | 15.4 | 15.4 | ⭤ | 0.0 | 100.0 | 0.0 | 0.0 | ⭤ | 61.5 | 38.5 | 0.0 | 0.0 | ⭤ |
| **Netherlands** | 7 | 3 | 0 | 0 | 4 | 57.1 | 14.3 | 28.6 | 0.0 | ⭤ | 0.0 | 71.4 | 14.3 | 14.3 | ⭤ | 0.0 | 100.0 | 0.0 | 0.0 | ⭤ | 71.4 | 28.6 | 0.0 | 0.0 | ⭤ |
| **Sweden** | 6 | 4 | 0 | 0 | 2 | 33.3 | 0.0 | 50.0 | 16.7 | ⭣ | 0.0 | 33.3 | 33.3 | 33.3 | ⮇ | 0.0 | 100.0 | 0.0 | 0.0 | ⭤ | 50.0 | 50.0 | 0.0 | 0.0 | ⮅ |
| **Denmark** | 6 | 1 | 0 | 0 | 5 | 66.7 | 0.0 | 33.3 | 0.0 | ⭤ | 16.7 | 33.3 | 16.7 | 33.3 | ⭤ | 0.0 | 100.0 | 0.0 | 0.0 | ⭤ | 50.0 | 50.0 | 0.0 | 0.0 | ⭤ |
| **Norway** | 5 | 3 | 0 | 0 | 2 | 33.3 | 0.0 | 50.0 | 16.7 | ⭤ | 0.0 | 16.7 | 16.7 | 66.7 | ⭣ | 0.0 | 100.0 | 0.0 | 0.0 | ⭤ | 100.0 | 0.0 | 0.0 | 0.0 | ⭤ |
| **France** | 2 | 1 | 0 | 1 | 0 | 0.0 | 0.0 | 100.0 | 0.0 | ⭣ | 0.0 | 0.0 | 0.0 | 100.0 | ⮇ | 0.0 | 100.0 | 0.0 | 0.0 | ⭤ | 0.0 | 100.0 | 0.0 | 0.0 | ⭤ |
| **Italy** | 2 | 0 | 1 | 1 | 0 | 0.0 | 0.0 | 100.0 | 0.0 | ⭣ | 50.0 | 50.0 | 0.0 | 0.0 | ⭤ | 0.0 | 100.0 | 0.0 | 0.0 | ⭤ | 100.0 | 0.0 | 0.0 | 0.0 | ⮅ |
| **Austria** | 1 | 1 | 0 | 0 | 0 | 100.0 | 0.0 | 0.0 | 0.0 | ⮅ | 0.0 | 100.0 | 0.0 | 0.0 | ⭤ | 0.0 | 100.0 | 0.0 | 0.0 | ⭤ | 0.0 | 100.0 | 0.0 | 0.0 | ⭤ |
| **Finland** | 1 | 1 | 0 | 0 | 0 | 100.0 | 0.0 | 0.0 | 0.0 | ⮅ | 0.0 | 0.0 | 0.0 | 100.0 | ⮇ | 0.0 | 100.0 | 0.0 | 0.0 | ⭤ | 0.0 | 100.0 | 0.0 | 0.0 | ⭤ |
| **Czech Republic** | 1 | 1 | 0 | 0 | 0 | 100.0 | 0.0 | 0.0 | 0.0 | ⮅ | 0.0 | 100.0 | 0.0 | 0.0 | ⭤ | 0.0 | 100.0 | 0.0 | 0.0 | ⭤ | 100.0 | 0.0 | 0.0 | 0.0 | ⮅ |
| **Belgium** | 1 | 1 | 0 | 0 | 0 | 100.0 | 0.0 | 0.0 | 0.0 | ⮅ | 0.0 | 0.0 | 0.0 | 100.0 | ⮇ | 0.0 | 100.0 | 0.0 | 0.0 | ⭤ | 100.0 | 0.0 | 0.0 | 0.0 | ⮅ |
| **Ireland** | 1 | 1 | 0 | 0 | 0 | 100.0 | 0.0 | 0.0 | 0.0 | ⮅ | 0.0 | 100.0 | 0.0 | 0.0 | ⭤ | 0.0 | 100.0 | 0.0 | 0.0 | ⭤ | 0.0 | 100.0 | 0.0 | 0.0 | ⮅ |
| **Multi-country (Torrent-Sellens et al. 2018 [38])** | 1 | 1 | 0 | 0 | 0 | 100.0 | 0.0 | 0.0 | 0.0 | ⮅ | 0.0 | 0.0 | 0.0 | 100.0 | ⮇ | 0.0 | 100.0 | 0.0 | 0.0 | ⭤ | 100.0 | 0.0 | 0.0 | 0.0 | ⮅ |

**Table S6.** Quantitative outcomes across eligible studies.

Table S6 was prepared in response to Reviewer W, Comment 6, which requested that quantitative ranges be provided for the subset of included studies reporting directly comparable discrete percentages. The TAM–NPT synthesis in Tables S1-S5 captures the direction of effects across all 65 studies but does not convey their magnitude. Table S6 addresses this gap by extracting observed percentage ranges from the subset of quantitative descriptive studies in which a single, clearly defined outcome was measured with a discrete denominator. No pooling or meta-analytic procedure is applied; the figures represent only the observed spread of reported values. Explicit caveats accompany each group to contextualize the heterogeneity in measurement instruments, denominators, time points, and technology types.

**Group 1 — Intention to Continue / Sustain e-Health Use Post-Pandemic**

*Outcome definition: % of GP practices or individual GPs intending to continue more intensive use of a specific e-health modality after the COVID-19 pandemic.*

| Study | Country | Technology | N (denominator) | % (value) |
| --- | --- | --- | --- | --- |
| Keuper et al., 2021 [46] | Netherlands | Video consultations | 1,083 practices (Apr); 718 (Jul) | Apr: 28%; Jul: 20% |
| Keuper et al., 2021 [46] | Netherlands | E-consultations | 1,083 practices (Apr); 718 (Jul) | Apr: 26%; Jul: 32% |
| Keuper et al., 2021 [46] | Netherlands | Teleconsultations (GP–specialist) | 1,083 practices (Apr); 718 (Jul) | Apr: 17%; Jul: 23% |
| Keuper et al., 2021 [46] | Netherlands | Online repeat prescriptions | 1,083 practices (Apr); 718 (Jul) | Apr: 14%; Jul: 23% |
| Keuper et al., 2021 [46] | Netherlands | Telemonitoring | 1,083 practices (Apr); 718 (Jul) | Apr: 7%; Jul: 5% |
| Johnsen et al., 2021 [72] | Norway | Video consultations (post-pandemic motivation) | 3,475 VC evaluations by 855 GPs | 49% (1,704/3,475) motivated to use VC for similar problem post-pandemic |

**Observed range: 5% – 49%**
Caveat: Keuper et al. denominators are GP practices, not individual GPs; the Johnsen figure is per-consultation, not per-GP. The two studies use different time points and measurement approaches. This range shows only the observed spread.

**Group 2 — GP Satisfaction with / Positive Assessment of Video Consultations and Telemedicine**

*Outcome definition: % of consultations or GPs rating a specific VC or telemedicine outcome positively.*

| Study | Country | Technology | N (denominator) | % (value) |
| --- | --- | --- | --- | --- |
| Johnsen et al., 2021 [72] | Norway | VCs: overall suitability equal to or better than face-to-face | 3,476 VCs (855 GPs) | 51% (1,766/3,476) |
| Johnsen et al., 2021 [72] | Norway | VCs with relational continuity: equal/better than F2F | 1,785 VCs | 57% (1,011/1,785) |
| Johnsen et al., 2021 [72] | Norway | VCs for follow-up (episodic continuity): equal/better | 1,919 VCs | 61% (1,165/1,919) |
| Johnsen et al., 2021 [72] | Norway | VCs for new problems: equal/better | 1,556 VCs | 35% (544/1,556) |
| Johnsen et al., 2021 [72] | Norway | GP perception: patient satisfied with VC | 3,475 VCs | 85% (2,967/3,475) |
| Johnsen et al., 2021 [72] | Norway | Technical quality rated "good" | 3,475 VCs | 90% (3,118/3,475) |
| Johnsen et al., 2021 [72] | Norway | VCs for mental illness/life stress: equal/better | 684 VCs | 74% (509/684) |
| Johnsen et al., 2021 [72] | Norway | VCs for administrative purposes: equal/better | 137 VCs | 78% (107/137) |
| Johnsen et al., 2021 [72] | Norway | VCs for skin disorders: equal/better | 300 VCs | 33% (98/300) |
| Johnsen et al., 2021 [72] | Norway | VCs for musculoskeletal problems: equal/better | 469 VCs | 40% (187/469) |
| Tensen et al., 2023 [78] | Netherlands | Digital dermatology pathway: positive/strongly positive experience | 66 GPs | 61% (40/66) |
| Tensen et al., 2023 [78] | Netherlands | Would use the platform again | 66 GPs | 92% (61/66) |
| Tensen et al., 2023 [78] | Netherlands | Would recommend platform to a colleague | 66 GPs | 80% (53/66) |
| Tensen et al., 2023 [78] | Netherlands | Received sufficient support during pandemic | 66 GPs | 91% (60/66) |
| Tensen et al., 2023 [78] | Netherlands | Digital dermatology home consultation: positive/strongly positive (among users only) | 10 GPs (15% used it) | 80% (8/10) |

**Observed range (GP/VC-level satisfaction or positive rating): 33% – 92%**

Caveat 1: Johnsen figures are per-consultation evaluations, not per-GP responses; one GP may contribute multiple data points. Caveat 2: Tensen's n = 66 is a low-response survey (response rate ~2% of invited GPs). Caveat 3: Tensen home consultation % (80%, n = 10) is based on a very small subset of users only.

**Group 3 — GP Adoption and Positive Attitude Rates for Digital Health Applications (DHAs / DiGA)**

*Outcome definition: % of GPs who have adopted a DHT, prescribed a digital application, or rate their attitude as positive.*

| Study | Country | Technology | N (denominator) | % (value) |
| --- | --- | --- | --- | --- |
| Breedvelt et al., 2019 [39] | UK | e-Mental health interventions: currently using | 1,044 GPs | 72% |
| Breedvelt et al., 2019 [39] | UK | Untrained in e-mental health | 1,044 GPs | 91.5% |
| Brecher et al., 2024 [56] | Germany | Positive attitude toward DiGA (prescribable digital health apps) | 97 family doctors | 61.1% |
| Brecher et al., 2024 [56] | Germany | Ever prescribed a DiGA | 97 family doctors | 59.4% |
| Brecher et al., 2024 [56] | Germany | Intention to prescribe DiGA within 12 months | 97 family doctors | 38.5% |
| Brecher et al., 2024 [56] | Germany | Aware of DiGA | 97 family doctors | 80.4% |
| Brecher et al., 2024 [56] | Germany | Feel sufficiently informed to prescribe DiGA | 97 family doctors | 38.1% |
| Wangler & Jansky, 2024 [65] | Germany | Positive about DHAs overall | 5,868 GPs | 68% |
| Wangler & Jansky, 2024 [65] | Germany | Experienced users rating DHAs as "very useful" | Subset with DHA experience (n not specified) | 85% |

**Observed range (adoption/positive attitude): 38.1% – 85% (72% if restricted to use-only outcomes)**

Caveat: Breedvelt (UK, 2019) covers e-mental health specifically; Brecher covers German DiGA only; Wangler & Jansky (2024) covers a broad DHA category in Germany. These are not identical constructs.

**Group 4 — GP Adoption of AI Tools**

*Outcome definition: % of GPs currently using AI tools in clinical practice.*

| Study | Country | Technology | N (denominator) | % (value) |
| --- | --- | --- | --- | --- |
| Blease et al., 2024 [89] | UK | Generative AI tools (ChatGPT etc.) in clinical practice | 1,006 GPs | 20% (205/1,006) |
| Blease et al., 2024 [89] | UK | Among AI users: using for documentation | 162 who answered follow-up | 29% (47/162) |
| Blease et al., 2024 [89] | UK | Among AI users: using for differential diagnosis | 162 who answered follow-up | 28% (45/162) |
| Mahlknecht et al., 2023 [86] | Italy | GP satisfaction with AI symptom checker (high satisfaction) | 10 GPs | 27% |
| Mahlknecht et al., 2023 [86] | Italy | GP rating clinical quality of AI tool as "appropriate" | 10 GPs | 31.1% |

**Observed range (AI adoption/satisfaction): 20% – 31%**

Caveat: Mahlknecht n = 10 is a very small feasibility study; results carry low statistical weight. Blease's 20% reflects use of generative AI broadly, not a satisfaction rate. These figures are not directly comparable to each other.

**Group 5 — EMR Adoption and Perceived Impact**

*Outcome definition: % of GPs who have adopted EMRs or perceive a specific work-related impact.*

| Study | Country | Technology | N (denominator) | % (value) |
| --- | --- | --- | --- | --- |
| Cabitza et al., 2014 [90] | Italy | EMR adoption (currently using) | 800 GPs | 98% |
| Cabitza et al., 2014 [90] | Italy | Work perceived as more documented/appropriate with EMR | 800 GPs | 90% |
| Cabitza et al., 2014 [90] | Italy | Work perceived as more stressful with EMR | 800 GPs | 43% |

**Observed range (adoption/positive impact): 43% – 98%**

*Sensitivity analysis regarding respondent overlap*

To assess the impact of potential respondent overlap across large multi-country and national surveys (Torrent-Sellens et al., 2018 [38]; Keuper et al., 2021 [46]; Wangler and Jansky, 2024 [65]), we repeated the TAM–NPT effect‑direction analysis after excluding the 3 largest studies (n = 62 instead of 65; DHT n = 33 instead of 36). These studies appeared to be from the DHT category only, therefore only results for this category and the overall domain are displayed and compared in the tables below, as other technology groups were not affected by the exclusion of studies. As shown in the sensitivity rows, changes in category percentages for both the overall sample and the affected DHT subgroup were below 2% in all TAM and NPT domains, and the qualitative patterns remained the same: perceived usefulness and actual use stayed predominantly positive, perceived ease of use remained predominantly negative, and NPT domains continued to indicate only partial normalization. This suggests that the observed implementation paradox is robust to plausible levels of double-counting in large survey studies.

**Table S7.** Technology Acceptance Model (TAM) analysis.

Percentage of studies reporting each response category by TAM construct and technology type. Values for DHT (n=33) and Overall (n=62) show the results after excluding the 3 largest studies. Below are the original values, including all studies displayed in grey for better comparability.

| **Construct / Response** | **YES** | **PARTIAL** | **UNCLEAR** | **NO** | **Effect direction** |
| --- | --- | --- | --- | --- | --- |
| **Perceived Usefulness** | | | | |  |
| DHT (n=33) | 84.9% | 12.1% | 3% | 0.0% | ⮅ |
| DHT (n=36) | 83.3% | 13.9% | 2.8% | 0.0% | ⮅ |
| Overall (n=62) | 87.1% | 11.3% | 1.6% | 0.0% | ⮅ |
| Overall (n=65) | 86.2% | 12.3% | 1.5% | 0.0% | ⮅ |
| **Perceived Ease of Use** | | | | |  |
| DHT (n=33) | 0.0% | 9.1% | 15.2% | 75.8% | ⮇ |
| DHT (n=36) | 0.0% | 11.1% | 13.9% | 75.0% | ⮇ |
| Overall (n=62) | 0.0% | 19.4% | 14.5% | 66.1% | ⮇ |
| Overall (n=65) | 0.0% | 20.0% | 13.8% | 66.2% | ⮇ |
| **Behavioral Intention** | | | | |  |
| DHT (n=33) | 0% | 90.9% | 0.0% | 9.1% | ⭤ |
| DHT (n=36) | 2.8% | 88.9% | 0.0% | 8.3% | ⭤ |
| Overall (n=62) | 3.2% | 85.5% | 0.0% | 11.3% | ⭤ |
| Overall (n=65) | 4.6% | 84.6% | 0.0% | 10.8% | ⭤ |
| **Actual Use** | | | | |  |
| DHT (n=33) | 93.9% | 5.1% | 0.0% | 0.0% | ⮅ |
| DHT (n=36) | 94.4% | 5.6% | 0.0% | 0.0% | ⮅ |
| Overall (n=62) | 96.8% | 3.2% | 0.0% | 0.0% | ⮅ |
| Overall (n=65) | 96.9% | 3.1% | 0.0% | 0.0% | ⮅ |

**Table S8.** Normalization Process Theory (NPT) analysis.

Percentage of studies reporting each response category by NPT construct and technology type. Values for DHT (n=33) and Overall (n=62) show the results after excluding the 3 largest studies. Below are the original values, including all studies displayed in grey for better comparability.

| **Construct / Response** | **YES** | **PARTIAL** | **UNCLEAR** | **NO** | **Effect direction** |
| --- | --- | --- | --- | --- | --- |
| **Coherence** | | | | |  |
| DHT (n=33) | 51.5% | 15.2% | 24.2% | 9.1% | ⭤ |
| DHT (n=36) | 52.8% | 16.7% | 22.2% | 8.3% | ⭤ |
| Overall (n=62) | 50.0% | 11.3% | 33.9% | 4.8% | ⭤ |
| Overall (n=65) | 50.8% | 12.3% | 32.3% | 4.6% | ⭤ |
| **Cognitive Participation** | | | | |  |
| DHT (n=33) | 3.0% | 57.6% | 12.1% | 27.3% | ⭤ |
| DHT (n=36) | 2.8% | 58.3% | 11.1% | 27.8% | ⭤ |
| Overall (n=62) | 4.8% | 46.8% | 14.5% | 33.9% | ⭤ |
| Overall (n=65) | 4.6% | 47.7% | 13.8% | 33.9% | ⭤ |
| **Collective Action** | | | | |  |
| DHT (n=33) | 0.0% | 100.0% | 0.0% | 0.0% | ⭤ |
| DHT (n=36) | 0.0% | 100.0% | 0.0% | 0.0% | ⭤ |
| Overall (n=62) | 0.0% | 100.0% | 0.0% | 0.0% | ⭤ |
| Overall (n=65) | 0.0% | 100.0% | 0.0% | 0.0% | ⭤ |
| **Reflexive Monitoring** | | | | |  |
| DHT (n=33) | 60.6% | 39.4% | 0.0% | 0.0% | ⭡ |
| DHT (n=36) | 63.9% | 36.1% | 0.0% | 0.0% | ⭡ |
| Overall (n=62) | 64.5% | 35.5% | 0.0% | 0.0% | ⭡ |
| Overall (n=65) | 66.2% | 33.9% | 0.0% | 0.0% | ⭡ |

*MMAT sensitivity analysis*
To assess the impact of the 4 studies that scored 60% in the MMAT rating, we repeated the TAM–NPT effect‑direction analysis after excluding these 4 studies (n = 61 instead of 65; DHT n = 33 instead of 36; TM n = 17 instead of 18). These studies appeared to be from the DHT and TM category only, therefore only results for these categories and the overall domain are displayed and compared in the tables below, as other technology groups were not affected by the exclusion of studies. The sensitivity analysis showed no change in the direction of conclusions across outcome domains; therefore, the summary patterns reported in the main text remained unchanged.

**Table S9.** Technology Acceptance Model (TAM) analysis.

Percentage of studies reporting each response category by TAM construct and technology type. Values for DHT (n=33), TM (n=17), and Overall (n=61) show the results after excluding the 4 studies. Below are the original values, including all studies displayed in grey for better comparability.

| **Construct / Response** | **YES** | **PARTIAL** | **UNCLEAR** | **NO** | **Effect direction** |
| --- | --- | --- | --- | --- | --- |
| **Perceived Usefulness** | | | | |  |
| DHT (n=33) | 84.9% | 15.2% | 0.0% | 0.0% | ⮅ |
| DHT (n=36) | 83.3% | 13.9% | 2.8% | 0.0% | ⮅ |
| TM (n=17) | 88.2% | 11.8% | 0.0% | 0.0% | ⮅ |
| TM (n=18) | 88.9% | 11.1% | 0.0% | 0.0% | ⮅ |
| Overall (n=61) | 86.9% | 13.1% | 0% | 0.0% | ⮅ |
| Overall (n=65) | 86.2% | 12.3% | 1.5% | 0.0% | ⮅ |
| **Perceived Ease of Use** | | | | |  |
| DHT (n=33) | 0.0% | 12.1% | 12.1% | 75.8% | ⮇ |
| DHT (n=36) | 0.0% | 11.1% | 13.9% | 75.0% | ⮇ |
| TM (n=17) | 0.0% | 29.4% | 11.8% | 58.8% | ⮇ |
| TM (n=18) | 0.0% | 27.8% | 16.7% | 55.6% |  |
| Overall (n=61) | 0.0% | 21.3% | 11.5% | 67.2% | ⮇ |
| Overall (n=65) | 0.0% | 20.0% | 13.8% | 66.2% | ⮇ |
| **Behavioral Intention** | | | | |  |
| DHT (n=33) | 3.0% | 87.9% | 0.0% | 9.1% | ⭤ |
| DHT (n=36) | 2.8% | 88.9% | 0.0% | 8.3% | ⭤ |
| TM (n=17) | 11.8% | 70.6% | 0.0% | 17.7% | ⭤ |
| TM (n=18) | 11.1% | 72.2% | 0.0% | 16.7% | ⭤ |
| Overall (n=61) | 4.9% | 83.6% | 0.0% | 11.5% | ⭤ |
| Overall (n=65) | 4.6% | 84.6% | 0.0% | 10.8% | ⮇ |
| **Actual Use** | | | | |  |
| DHT (n=33) | 93.8% | 6.3% | 0.0% | 0.0% | ⮅ |
| DHT (n=36) | 94.4% | 5.6% | 0.0% | 0.0% | ⮅ |
| TM (n=17) | 100.0% | 0.0% | 0.0% | 0.0% | ⮅ |
| TM (n=18) | 100.0% | 0.0% | 0.0% | 0.0% | ⮅ |
| Overall (n=61) | 96.7% | 3.3% | 0.0% | 0.0% | ⮅ |
| Overall (n=65) | 96.9% | 3.1% | 0.0% | 0.0% | ⮅ |

**Table S10.** Normalization Process Theory (NPT) analysis.

Percentage of studies reporting each response category by NPT construct and technology type. Values for DHT (n=33), TM (n=17), and Overall (n=61) show the results after excluding the 4 studies. Below are the original values, including all studies displayed in grey for better comparability.

| **Construct / Response** | **YES** | **PARTIAL** | **UNCLEAR** | **NO** | **Effect direction** |
| --- | --- | --- | --- | --- | --- |
| **Coherence** | | | | |  |
| DHT (n=33) | 48.4% | 18.2% | 24.2% | 9.1% | ⭤ |
| DHT (n=36) | 52.8% | 16.7% | 22.2% | 8.3% | ⭤ |
| TM (n=17) | 52.9% | 0.0% | 47.1% | 0.0% | ⭤ |
| TM (n=18) | 50.0% | 0.0% | 50.0% | 0.0% | ⭤ |
| Overall (n=61) | 49.2% | 13.1% | 32.8% | 4.9% | ⭡ |
| Overall (n=65) | 50.8% | 12.3% | 32.3% | 4.6% | ⭡ |
| **Cognitive Participation** | | | | |  |
| DHT (n=33) | 3.0% | 60.6% | 9.1% | 27.3% | ⭤ |
| DHT (n=36) | 2.8% | 58.3% | 11.1% | 27.8% | ⭤ |
| TM (n=17) | 5.9% | 35.1% | 23.5% | 35.3% | ⭤ |
| TM (n=18) | 5.6% | 33.3% | 27.8% | 33.3% | ⭤ |
| Overall (n=61) | 4.9% | 49.2% | 11.5% | 34.4% | ⭤ |
| Overall (n=65) | 4.6% | 47.7% | 13.8% | 33.9% | ⭤ |
| **Collective Action** | | | | |  |
| DHT (n=33) | 0.0% | 100.0% | 0.0% | 0.0% | ⭤ |
| DHT (n=36) | 0.0% | 100.0% | 0.0% | 0.0% | ⭤ |
| TM (n=17) | 0.0% | 100.0% | 0.0% | 0.0% | ⭤ |
| TM (n=18) | 0.0% | 100.0% | 0.0% | 0.0% | ⭤ |
| Overall (n=61) | 0.0% | 100.0% | 0.0% | 0.0% | ⭤ |
| Overall (n=65) | 0.0% | 100.0% | 0.0% | 0.0% | ⭤ |
| **Reflexive Monitoring** | | | | |  |
| DHT (n=33) | 63.6% | 36.4% | 0.0% | 0.0% | ⭡ |
| DHT (n=36) | 63.9% | 36.1% | 0.0% | 0.0% | ⭡ |
| TM (n=17) | 58.8% | 41.2% | 0.0% | 0.0% | ⭡ |
| TM (n=18) | 61.1% | 38.9% | 0.0% | 0.0% | ⭡ |
| Overall (n=61) | 65.6% | 34.4% | 0.0% | 0.0% | ⭡ |
| Overall (n=65) | 66.2% | 33.9% | 0.0% | 0.0% | ⭡ |

**Abbreviations**

AI: Artificial intelligence

CDSS: Clinical decision support system

DHA: Digital health application

DHT: Digital health tool

EMR: Electronic medical record

GP: General practitioner

MMAT: Mixed Methods Appraisal Tool

NPT: Normalization Process Theory

TAM: Technology Acceptance Model

TM: Telemedicine

UK: United Kingdom

VC: Video consultation

**References**

38. Torrent-Sellens J, Díaz-Chao Á, Soler-Ramos I, Saigí-Rubió F. Modeling and predicting outcomes of eHealth usage by European physicians: multidimensional approach from a survey of 9196 general practitioners. J Med Internet Res. Oct 22, 2018;20(10):e279. [doi: 10.2196/jmir.9253] [Medline: 30348628]

46. Keuper J, Batenburg R, Verheij R, van Tuyl L. Use of e-Health in Dutch general practice during the COVID-19 pandemic. Int J Environ Res Public Health. Nov 26, 2021;18(23):12479. [doi: 10.3390/ijerph182312479] [Medline: 34886204]

72. Johnsen TM, Norberg BL, Kristiansen E, et al. Suitability of video consultations during the COVID-19 pandemic lockdown: cross-sectional survey among Norwegian general practitioners. J Med Internet Res. Feb 8, 2021;23(2):e26433. [doi: 10.2196/26433] [Medline: 33465037]

78. Tensen E, Kuziemsky C, Jaspers MW, Peute LW. General practitioners’ perspectives about remote dermatology care during the COVID-19 pandemic in the Netherlands: questionnaire-based study. JMIR Dermatol. Jun 13, 2023;6:e46682. [doi: 10.2196/46682] [Medline: 37632975]

39. Breedvelt JJ, Zamperoni V, Kessler D, et al. GPs’ attitudes towards digital technologies for depression: an online survey in primary care. Br J Gen Pract. Mar 2019;69(680):e164-e170. [doi: 10.3399/bjgp18X700721] [Medline: 30559111]

56. Brecher AL, Herrgen J, Mueller T. DiGA in primary care and the influence of patient-related factors - results of a survey of family doctors. Hayn D, Pfeifer B, Schreier G, Baumgartner M, editors. Studies in Health Technology and Informatics. Apr 26, 2024;313:55-61. [doi: 10.3233/SHTI240012] [Medline: 38682505]

65. Wangler J, Jansky M. How can primary care benefit from digital health applications? – A quantitative, explorative survey on attitudes and experiences of general practitioners in Germany. BMC Digit Health. 2024;2(1):14. [doi: 10. 1186/s44247-024-00068-x]

89. Blease CR, Locher C, Gaab J, Hägglund M, Mandl KD. Generative artificial intelligence in primary care: an online survey of UK general practitioners. BMJ Health Care Inform. Aug 2024;31(1):e101102. [doi: 10.1136/bmjhci-2024- 101102] [Medline: 39288998]

86. Mahlknecht A, Engl A, Piccoliori G, Wiedermann CJ. Supporting primary care through symptom checking artificial intelligence: a study of patient and physician attitudes in Italian general practice. BMC Prim Care. Sep 4, 2023;24(1):174. [doi: 10.1186/s12875-023-02143-0] [Medline: 37661285]

90. Cabitza F, Misericordia P, Del Zotti F. Electronic records for general practice - where we are, where we should head to improve them. Presented at: International Conference on Health Informatics; Mar 3-4, 2014:535-542; ESEO, Angers, Loire Valley, France. [doi: 10.5220/0004924805350542]
