# Supplementary material for: The Impact of Digitalization on European General Practice From the Perspective of General Practitioners: Systematic Review
Source: J Med Internet Res. 2026 Aug 18;28:e83343. doi: 10.2196/83343 (PMC13485116; doi:10.2196/83343)
Supplement: Supplementary file 7 [file jmir-v28-e83343-s008.docx]

**PRISMA 2020 Checklist**

| **Section and Topic** | **Item #** | **Checklist item** | **Location where item is reported** | **Exact Content/Section** |
| --- | --- | --- | --- | --- |
| **TITLE** | | |  |  |
| Title | 1 | Identify the report as a systematic review. | **Page 1, Title** | "The impact of digitalization on European general practice from the perspective of general practitioners - Systematic review" |
| **ABSTRACT** | | |  |  |
| Abstract | 2 | See the PRISMA 2020 for Abstracts checklist. | **Pages 2-4, Abstract section** | Structured abstract with Background, Objective, Methods, Results, Conclusion sections |
| **INTRODUCTION** | | |  |  |
| Rationale | 3 | Describe the rationale for the review in the context of existing knowledge. | **Pages 4-5, Introduction > Background** | "Several reviews have examined digital health in primary care..." through entire gap analysis paragraph ending "...this review offers a dedicated synthesis of European GPs' subjective experiences..." |
| Objectives | 4 | Provide an explicit statement of the objective(s) or question(s) the review addresses. | **Page 4, Study aims and objective** | "This systematic review explores GPs' perspectives on digital health integration, focusing on: Adoption trends... GPs' experiences... Impacts on workload... Barriers... Policy recommendations..." |
| **METHODS** | | |  |  |
| Eligibility criteria | 5 | Specify the inclusion and exclusion criteria for the review and how studies were grouped for the syntheses. | **Page 5, Methods > Eligibility criteria** | Complete PICO criteria: Population (GPs in Europe), Phenomenon (digital health), Language (English/German), Study design, Outcomes, Time frame (2014-2024) |
| Information sources | 6 | Specify all databases, registers, websites, organisations, reference lists and other sources searched or consulted to identify studies. Specify the date when each source was last searched or consulted. | **Page 5, Methods > Search strategy** | "A comprehensive search was conducted across 8 electronic databases: PubMed, Scopus, SpringerLink, Wiley Online Library, Cochrane Library, Web of Science, Google Scholar, and Ovid... Elicit AI... primary search completed January 5, 2025... search update October 7, 2025" |
| Search strategy | 7 | Present the full search strategies for all databases, registers and websites, including any filters and limits used. | **Page 5 + MA 1** | Boolean operators described on page 5; full database-specific strings in **Multimedia Appendix 1** |
| Selection process | 8 | Specify the methods used to decide whether a study met the inclusion criteria of the review, including how many reviewers screened each record and each report retrieved, whether they worked independently, and if applicable, details of automation tools used in the process. | **Page 6, Methods > Study selection** | "One primary reviewer (JF) conducted screening. A second reviewer (LN) independently screened 20%... Inter-rater agreement: 95.86% (title), 95% (abstract), 99% (full-text)... κ values 0.84, 0.88, 0.92... Rayyan web application" |
| Data collection process | 9 | Specify the methods used to collect data from reports, including how many reviewers collected data from each report, whether they worked independently, any processes for obtaining or confirming data from study investigators, and if applicable, details of automation tools used in the process. | **Page 8, Methods > Data extraction** | "Elicit AI and JF performed data extraction... training on 5 studies... 100% overlap... JF cross-checked all Elicit extractions... No authors contacted" |
| Data items | 10a | List and define all outcomes for which data were sought. Specify whether all results that were compatible with each outcome domain in each study were sought (e.g. for all measures, time points, analyses), and if not, the methods used to decide which results to collect. | **Page 8, Methods > Data extraction + MA 1, MA 3** | Bulleted list: "Reported perceptions... Impact on clinical practice... workflow efficiency... patient relationships... Perceived benefits... challenges... Policy implications" |
|  | 10b | List and define all other variables for which data were sought (e.g. participant and intervention characteristics, funding sources). Describe any assumptions made about any missing or unclear information. | **Page 8, Methods > Data extraction + MA 1, MA 3** | "Author(s), publication year, country... Study design... Participant characteristics... Type of digital health intervention... No authors contacted for missing data" |
| Study risk of bias assessment | 11 | Specify the methods used to assess risk of bias in the included studies, including details of the tool(s) used, how many reviewers assessed each study and whether they worked independently, and if applicable, details of automation tools used in the process. | **Pages 8-9, Methods > Quality assessment** | "Mixed Methods Appraisal Tool (MMAT) 2018... assessed manually by JF and checked by other authors... MMAT ratings in Multimedia Appendix 4" |
| Effect measures | 12 | Specify for each outcome the effect measure(s) (e.g. risk ratio, mean difference) used in the synthesis or presentation of results. | **Page 9, Methods > Quality assessment** | "Effect-direction table... direction-of-effect categories (strong positive, positive, mixed, negative, strong negative) using pre-specified percentage thresholds" |
| Synthesis methods | 13a | Describe the processes used to decide which studies were eligible for each synthesis (e.g. tabulating the study intervention characteristics and comparing against the planned groups for each synthesis (item #5)). | **Page 8, Methods > Quality assessment** | "Studies grouped into four mutually exclusive categories (AI, DHTs, EHRs, telemedicine)... No changes made to planned groupings after protocol registration" |
|  | 13b | Describe any methods required to prepare the data for presentation or synthesis, such as handling of missing summary statistics, or data conversions. | **Study protocol (OSF)** | Referenced in manuscript page 5: "protocol registered... OSF... DOI: 10.17605/OSF.IO/KE25C" |
|  | 13c | Describe any methods used to tabulate or visually display results of individual studies and syntheses. | **Pages 8–9 (Methods, data presentation), 10–12 (Tables 1–2, Figure 2), ~20–22 (TAM–NPT effect‑direction table), MA 3 and MA 5.** |  |
|  | 13d | Describe any methods used to synthesize results and provide a rationale for the choice(s). If meta-analysis was performed, describe the model(s), method(s) to identify the presence and extent of statistical heterogeneity, and software package(s) used. | **Pages 8-9, Methods > Quality assessment** | "Narrative synthesis... Popay et al. (2006) framework... TAM and NPT... effect-direction mapping... heterogeneity precluded meta-analysis... quantitative meta-analysis would risk 'fruit-salad' summary" |
|  | 13e | Describe any methods used to explore possible causes of heterogeneity among study results (e.g. subgroup analysis, meta-regression). | **Page 9, Methods > Quality assessment** | "Studies grouped... by technology type... geographic distribution analyzed descriptively" |
|  | 13f | Describe any sensitivity analyses conducted to assess robustness of the synthesized results. | **Page 9, Methods > Quality assessment + MA 7** | "Limited sensitivity analyses for respondent overlap and study quality (MMAT) reported in Multimedia Appendix 7 as side-by-side comparison tables... changes below 2%, effect-direction arrows unchanged" |
| Reporting bias assessment | 14 | Describe any methods used to assess risk of bias due to missing results in a synthesis (arising from reporting biases). | **Page 9, Methods > Quality assessment** | "No formal reporting-bias assessment... because evidence was highly heterogeneous, largely non-comparative, and synthesis was qualitative and descriptive" |
| Certainty assessment | 15 | Describe any methods used to assess certainty (or confidence) in the body of evidence for an outcome. | **Page 9, Methods > Quality assessment** | "We did not apply a formal GRADE approach... used MMAT as pragmatic indicator... certainty ratings should be interpreted cautiously" |
| **RESULTS** | | |  |  |
| Study selection | 16a | Describe the results of the search and selection process, from the number of records identified in the search to the number of studies included in the review, ideally using a flow diagram. | **Page 7, Results (before Study characteristics) + Figure 1** | "The initial search yielded 1,580 references... 251 duplicates removed... 1,329 unique articles... 929 excluded at title... 400 abstracts assessed... 118 full-text... 65 included" + **PRISMA flow diagram Figure 1** |
|  | 16b | Cite studies that might appear to meet the inclusion criteria, but which were excluded, and explain why they were excluded. | **Page 7 + MA 2** | "53 full-text articles were excluded... detailed list in Multimedia Appendix 2" |
| Study characteristics | 17 | Cite each included study and present its characteristics. | **Pages 10-12, Results > Study characteristics + MA 5** | Sample sizes, study design/methods, temporal distribution (Table 1), geographic distribution (Table 2, Figure 2) + **Evidence Table in MA 5** |
| Risk of bias in studies | 18 | Present assessments of risk of bias for each included study. | **MA 4** | "MMAT ratings... 4 studies scored 60%, remaining 61 scored ≥80%... vote-counting analysis for 60% studies... Multimedia Appendix 4" |
| Results of individual studies | 19 | For all outcomes, present, for each study: (a) summary statistics for each group (where appropriate) and (b) an effect estimate and its precision (e.g. confidence/credible interval), ideally using structured tables or plots. | **Pages 8-9 + MA 3, MA 5** | AM-NPT coding for each study detailed in **Multimedia Appendix 3** (topic clustering template) and **MA 5** (Evidence Table) |
| Results of syntheses | 20a | For each synthesis, briefly summarise the characteristics and risk of bias among contributing studies. | **Pages 9-24 + MA 4** | Narrative synthesis sections 1-5 (Adoption trends, GP experiences, Impacts, Barriers, Policy recommendations) + TAM-NPT analysis + "MMAT scores... no study below 60%" |
|  | 20b | Present results of all statistical syntheses conducted. If meta-analysis was done, present for each the summary estimate and its precision (e.g. confidence/credible interval) and measures of statistical heterogeneity. If comparing groups, describe the direction of the effect. | **Pages 9-24, Table 3 + MA 7** | AM-NPT Effect Direction Plot (Table 3) with percentages; geographic patterns; technology-specific variations; **MA 7** full breakdown |
|  | 20c | Present results of all investigations of possible causes of heterogeneity among study results. | **Page 9 (NA explicitly stated)** | "Given marked heterogeneity... did not undertake formal meta-analysis" + descriptive country/technology groupings |
|  | 20d | Present results of all sensitivity analyses conducted to assess the robustness of the synthesized results. | **Page 9 (referenced in MA 7)** | "Sensitivity analyses... excluding three large surveys and four 60%-MMAT studies... changes <2%... patterns robust... Multimedia Appendix 7" |
| Reporting biases | 21 | Present assessments of risk of bias due to missing results (arising from reporting biases) for each synthesis assessed. | **Page 9 (referenced in MA 7)** | "No formal reporting-bias assessment... highly heterogeneous, non-comparative, qualitative synthesis" |
| Certainty of evidence | 22 | Present assessments of certainty (or confidence) in the body of evidence for each outcome assessed. | **Page 9 (referenced in MA 7)** | "We did not apply formal GRADE... MMAT used descriptively to characterize quality" |
| **DISCUSSION** | | |  |  |
| Discussion | 23a | Provide a general interpretation of the results in the context of other evidence. | **Pages 24-26, Discussion** | Entire Discussion section compares findings to external literature: workload paradoxes compared to Keuper et al., Fletcher et al.; inequities compared to Badr et al.; AI barriers compared to trust literature, etc. |
|  | 23b | Discuss any limitations of the evidence included in the review. | **Pages 23-24, Strengths and limitations** | "Eurocentric focus... language bias (6 excluded for language)... no RCTs... self-report bias... low response rates... MMAT without GRADE" |
|  | 23c | Discuss any limitations of the review processes used. | **Pages 23-24, Strengths and limitations** | "Search strategy not formally peer-reviewed... selection/extraction not fully independent... Elicit AI use limits exact replication" |
|  | 23d | Discuss implications of the results for practice, policy, and future research. | **Pages 24-26, Discussion > Implications + Future research** | "For policy and practice... interoperable systems... reimbursement... training... governance" + "Future research should... longitudinal studies... expand to Eastern/Southern Europe... evaluate hybrid models" |
| **OTHER INFORMATION** | | |  |  |
| Registration and protocol | 24a | Provide registration information for the review, including register name and registration number, or state that the review was not registered. | **Page 5, Methods > Study design** | "Protocol registered retrospectively with Open Science Framework (DOI: 10.17605/OSF.IO/KE25C) on May 31, 2025, after initial search but before extraction, synthesis, analysis" |
|  | 24b | Indicate where the review protocol can be accessed, or state that a protocol was not prepared. | **Page 5, Methods > Study design** | OSF link: https://osf.io/ke25c/ |
|  | 24c | Describe and explain any amendments to information provided at registration or in the protocol. | **Page 5, Methods > Study design** | "Registered retrospectively... after initial search, but before data extraction, synthesis, and analysis" |
| Support | 25 | Describe sources of financial or non-financial support for the review, and the role of the funders or sponsors in the review. | **Page 26, Funding** | "No funding grants... APF payment financed by Johannes Kepler University, Linz" |
| Competing interests | 26 | Declare any competing interests of review authors. | **Page 26, Conflicts of Interest** | "Non-declared" |
| Availability of data, code and other materials | 27 | Report which of the following are publicly available and where they can be found: template data collection forms; data extracted from included studies; data used for all analyses; analytic code; any other materials used in the review. | **Page 26, Data Availability** | "All data... publicly available through databases... complete list of included studies in Multimedia Appendices 3 and 5" |

*From:*  Page MJ, McKenzie JE, Bossuyt PM, Boutron I, Hoffmann TC, Mulrow CD, et al. The PRISMA 2020 statement: an updated guideline for reporting systematic reviews. BMJ 2021;372:n71. doi: 10.1136/bmj.n71. This work is licensed under CC BY 4.0. To view a copy of this license, visit <https://creativecommons.org/licenses/by/4.0/>

**PRISMA 2020 for Abstracts Checklist**

| **Section and Topic** | **Item #** | **Checklist item** | **Reported (Yes/No)** |
| --- | --- | --- | --- |
| **TITLE** | | |  |
| Title | 1 | Identify the report as a systematic review. | Yes |
| **BACKGROUND** | | |  |
| Objectives | 2 | Provide an explicit statement of the main objective(s) or question(s) the review addresses. | Yes |
| **METHODS** | | |  |
| Eligibility criteria | 3 | Specify the inclusion and exclusion criteria for the review. | Yes |
| Information sources | 4 | Specify the information sources (e.g. databases, registers) used to identify studies and the date when each was last searched. | Yes |
| Risk of bias | 5 | Specify the methods used to assess risk of bias in the included studies. | Yes |
| Synthesis of results | 6 | Specify the methods used to present and synthesise results. | Yes |
| **RESULTS** | | |  |
| Included studies | 7 | Give the total number of included studies and participants and summarise relevant characteristics of studies. | Yes |
| Synthesis of results | 8 | Present results for main outcomes, preferably indicating the number of included studies and participants for each. If meta-analysis was done, report the summary estimate and confidence/credible interval. If comparing groups, indicate the direction of the effect (i.e. which group is favoured). | Yes |
| **DISCUSSION** | | |  |
| Limitations of evidence | 9 | Provide a brief summary of the limitations of the evidence included in the review (e.g. study risk of bias, inconsistency and imprecision). | Yes |
| Interpretation | 10 | Provide a general interpretation of the results and important implications. | Yes |
| **OTHER** | | |  |
| Funding | 11 | Specify the primary source of funding for the review. | Yes |
| Registration | 12 | Provide the register name and registration number. | Yes |

*From:*  Page MJ, McKenzie JE, Bossuyt PM, Boutron I, Hoffmann TC, Mulrow CD, et al. The PRISMA 2020 statement: an updated guideline for reporting systematic reviews. BMJ 2021;372:n71. doi: 10.1136/bmj.n71. This work is licensed under CC BY 4.0. To view a copy of this license, visit <https://creativecommons.org/licenses/by/4.0/>

**PRISMA-S Checklist**

| **Section/topic** | **#** | **Checklist item** | **Location(s) Reported** | **Exact Content/Section** |
| --- | --- | --- | --- | --- |
| **INFORMATION SOURCES AND METHODS** | | | |  |
| Database name | 1 | Name each individual database searched, stating the platform for each. | **Page 5, Methods > Search strategy + MA 1 + Study protocol** | "8 electronic databases: PubMed, Scopus, SpringerLink, Wiley Online Library, Cochrane Library, Web of Science, Google Scholar, Ovid" (platforms implied by database names) |
| Multi-database searching | 2 | If databases were searched simultaneously on a single platform, state the name of the platform, listing all of the databases searched. | **Page 5 Methods: Search strategy, Study protocol** | Not applicable - each database searched individually |
| Study registries | 3 | List any study registries searched. | **Page 5 + Study protocol** | **No registries searched** - explicitly stated: "No additional sources... were used to identify studies beyond the specified databases and Elicit AI" |
| Online resources and browsing | 4 | Describe any online or print source purposefully searched or browsed (e.g., tables of contents, print conference proceedings, web sites), and how this was done. | **Page 5, Methods > Search strategy + Study protocol** | "Elicit AI... browser-based AI-powered literature review assistant... to identify additional gray literature" |
| Citation searching | 5 | Indicate whether cited references or citing references were examined, and describe any methods used for locating cited/citing references (e.g., browsing reference lists, using a citation index, setting up email alerts for references citing included studies). | **Page 5, Methods > Search strategy** | **NOT performed** - explicitly stated: "No additional sources, such as reference lists of included studies, manual journal browsing, or direct contact... were used" |
| Contacts | 6 | Indicate whether additional studies or data were sought by contacting authors, experts, manufacturers, or others. | **Page 5, Methods > Search strategy + Page 8** | **NOT performed** - "No additional sources... direct contact with authors or organizations... No authors were contacted for missing or unclear data" |
| Other methods | 7 | Describe any additional information sources or search methods used. | **Page 5 + Study protocol** | Elicit AI described in detail |
| **SEARCH STRATEGIES** | | | |  |
| Full search strategies | 8 | Include the search strategies for each database and information source, copied and pasted exactly as run. | **Page 5, Methods > Search strategy + MA 1** | Boolean operators on page 5: "("digital" OR "digitalization" OR "eHealth"...) AND ("primary care" OR "general practice"...)". **Full database-specific strings in Multimedia Appendix 1** |
| Limits and restrictions | 9 | Specify that no limits were used, or describe any limits or restrictions applied to a search (e.g., date or time period, language, study design) and provide justification for their use. | **Pages 5-6, Methods > Eligibility criteria + Search strategy + Pages 30, MA 1, 2** | "Language: English or German... based on review team's linguistic capabilities... Time frame: January 2014–December 2024... captures recent advances... 6 records excluded solely due to language (Spanish, French, Italian, Dutch) summarized in MA 2" |
| Search filters | 10 | Indicate whether published search filters were used (as originally designed or modified), and if so, cite the filter(s) used. | **Pages 5-6, Methods > Search strategy + MA 1, 2** | Boolean operators described; no published validated filters used |
| Prior work | 11 | Indicate when search strategies from other literature reviews were adapted or reused for a substantive part or all of the search, citing the previous review(s). | **Pages 5-6, MA 1** | "Search strategy developed iteratively by JF... PRESS 2015 retrospective assessment... PRESS analysis table in MA 1" |
| Updates | 12 | Report the methods used to update the search(es) (e.g., rerunning searches, email alerts). | **Pages 5-6, Methods > Search strategy** | "Search update performed October 7, 2025, by rerunning entire search using identical search strings and databases... January 1–October 7, 2025... updated results in Multimedia Appendix 8, but not included in present synthesis" |
| Dates of searches | 13 | For each search strategy, provide the date when the last search occurred. | **Pages 5-6, MA 1** | "Primary search completed January 5, 2025... search update October 7, 2025" |
| **PEER REVIEW** | | | |  |
| Peer review | 14 | Describe any search peer review process. | **Pages 5-6, Methods > Search strategy + Page 30 + MA 1** | **NOT prospectively peer-reviewed** - explicitly stated: "Search strategy developed iteratively by JF and was not prospectively peer-reviewed using a standardized checklist. After completing searches, retrospective PRESS 2015 assessment confirmed strategy was appropriate and thorough. PRESS analysis table in Multimedia Appendix 1." |
| **MANAGING RECORDS** | | | |  |
| Total Records | 15 | Document the total number of records identified from each database and other information sources. | **Page 7, Results + MA 1** | "1,580 references... 251 duplicates removed... 1,329 unique articles" - breakdown by database would be in MA 1 or PRISMA diagram |
| Deduplication | 16 | Describe the processes and any software used to deduplicate records from multiple database searches and other information sources. | **Page 6, Methods > Study selection + Study protocol** | "Automatic and manual deduplication in Rayyan web application, 251 duplicate entries removed" |
|  |  |  |  |  |
| PRISMA-S: An Extension to the PRISMA Statement for Reporting Literature Searches in Systematic Reviews | | |  |  |
| Rethlefsen ML, Kirtley S, Waffenschmidt S, Ayala AP, Moher D, Page MJ, Koffel JB, PRISMA-S Group. | | |  |  |
| Last updated February 27, 2020. | |  |  |  |

**Abbreviations**

AI: Artificial intelligence

DHT: Digital health tool

EHR: Electronic health record

GP: General practitioner

GRADE: Grading of Recommendations Assessment, Development, and Evaluation

MMAT: Mixed Methods Appraisal Tool

NPT: Normalization Process Theory

OSF: Open Science Framework

PRESS: Peer Review of Electronic Search Strategies

PRISMA: Preferred Reporting Items for Systematic Reviews and Meta-Analyses

RCT: Randomized controlled trial

TAM: Technology Acceptance Model
