# Supplementary material for: The Impact of Digitalization on European General Practice From the Perspective of General Practitioners: Systematic Review
Source: J Med Internet Res. 2026 Aug 18;28:e83343. doi: 10.2196/83343 (PMC13485116; doi:10.2196/83343)
Supplement: Supplementary file 8 [file jmir-v28-e83343-s009.docx]

The citation for the Synthesis Without Meta-analysis explanation and elaboration article is: Campbell M, McKenzie JE, Sowden A, Katikireddi SV, Brennan SE, Ellis S, Hartmann-Boyce J, Ryan R, Shepperd S, Thomas J, Welch V, Thomson H. Synthesis without meta-analysis (SWiM) in systematic reviews: reporting guideline BMJ 2020;368:l6890 <http://dx.doi.org/10.1136/bmj.l6890>

| **SWiM is intended to complement and be used as an extension to PRISMA** | | | |
| --- | --- | --- | --- |
| **SWiM reporting item** | **Item description** | **Page in manuscript where item is reported** | **Other*** |
| *Methods* | | | |
| **1** Grouping studies for synthesis | 1a) Provide a description of, and rationale for, the groups used in the synthesis (e.g., groupings of populations, interventions, outcomes, study design) | **Page 8, Methods > Quality assessment, paragraph 3; Results → “Study characteristics” (subsections “Study design and methods”, “Temporal distribution”, “Geographic distribution”)**  **Pages 10–12; Results → “Summary of main findings”**  **Pages 12–24: all subsections consistently refer back to the four technology groups (AI, DHTs, EHRs, telemedicine).** | The main synthesis groups are the four technology categories (AI, digital health tools [DHTs], electronic health records/electronic medical records [EHRs/EMRs], and telemedicine). These are described and justified in the methods (page 8, Methods > Quality assessment and data synthesis, paragraph 3), where we explain that each study was assigned to exactly one category, with subcategories providing additional granularity, and that studies covering multiple technologies without a specific focus on AI, EMRs, or telemedicine were classified as DHTs. The same groupings are then used consistently in the Results (Study characteristics and Summary of main findings, pages 10–24) and in Multimedia Appendix 3 (topic coding and extracted data). |
|  | 1b) Detail and provide rationale for any changes made subsequent to the protocol in the groups used in the synthesis | **Page 8, Methods > Quality assessment and data synthesis, paragraph 3** | The methods (page 8, Methods > Quality assessment and data synthesis) explicitly state that ‘No changes were made to the planned groupings after protocol registration.’ The planned groupings are documented in the OSF protocol (DOI: 10.17605/OSF.IO/KE25C), and the manuscript confirms that the four technology categories (AI, DHTs, EHRs, telemedicine) were used as originally specified. |
| **2** Describe the standardised metric and transformation methods used | Describe the standardised metric for each outcome. Explain why the metric(s) was chosen, and describe any methods used to transform the intervention effects, as reported in the study, to the standardised metric, citing any methodological guidance consulted | **Page 9, Methods > Quality assessment, paragraph 1** | The standardised metric used for synthesis is an effect‑direction categorisation across Technology Acceptance Model (TAM) and Normalization Process Theory (NPT) constructs. As described on page 9 (Methods > Quality assessment and data synthesis, first paragraph), study‑level results were transformed into five direction‑of‑effect categories (strong positive, positive, mixed, negative, strong negative) using pre‑specified percentage thresholds, and the proportion of studies in each category was then summarised. The manuscript states that ‘No further statistical transformations of effect estimates were undertaken.’ These summaries are presented in the TAM–NPT effect‑direction analysis and Table 3 in the Results (approximately pages 20–22) and in more detail in Multimedia Appendix 7. |
| **3** Describe the synthesis methods | Describe and justify the methods used to synthesise the effects for each outcome when it was not possible to undertake a meta-analysis of effect estimates | **Page 2, Abstract > Methods;**  **Pages 8-9, Methods > Quality assessment, paragraphs 4-6** | The synthesis methods are summarised in the Abstract (page 2, Methods: ‘Data were synthesized using Popay et al.'s [24] narrative synthesis framework and a combined Technology Acceptance Model (TAM) and Normalization Process Theory (NPT) effect‑direction approach’) and described in full in the methods section (pages 8–9, Methods > Quality assessment and data synthesis). Because the evidence base is highly heterogeneous, largely non‑comparative, and spans qualitative, quantitative, and mixed‑methods studies with non‑comparable outcome measures, the authors did not conduct a meta‑analysis. Instead, they used a structured narrative synthesis following Popay et al. (2006) [24], combined with vote counting and effect‑direction mapping across TAM and NPT constructs. The rationale for this choice (risk of a misleading ‘fruit‑salad’ meta‑analysis) and the stepwise approach (grouping by technology, computing descriptive statistics, transforming study‑level findings into effect‑direction categories, and visualising them in an effect‑direction table) are explicitly described on pages 8–9. |
| **4** Criteria used to prioritise results for summary and synthesis | Where applicable, provide the criteria used, with supporting justification, to select the particular studies, or a particular study, for the main synthesis or to draw conclusions from the synthesis (e.g., based on study design, risk of bias assessments, directness in relation to the review question) | **Page 8, paragraph 3–4, Methods → “Quality assessment and data synthesis”**  **Pages 12–24, Results → “Summary of main findings” (all subsections)** | The review does not privilege individual studies but instead prioritises patterns across pre‑specified strata. As outlined on page 8 (Methods > Quality assessment and data synthesis), studies are grouped into four mutually exclusive technology categories (AI, DHTs, EHRs, telemedicine), and descriptive statistics (frequencies and percentages) are used to characterise methodological approaches, temporal and geographic distributions, and quantitative findings. In the Results (pages 12–24, Summary of main findings), the authors structure their narrative by outcome domain (adoption, GP experiences, workload and ethical impacts, barriers, and policy implications) and consistently summarise findings within and across the four technology groups, and where relevant by country and time period (e.g. pre‑ vs post‑COVID‑19). Study quality (MMAT) is used descriptively (pages 8–9 and Multimedia Appendix 4) and is reflected at group level in the TAM–NPT effect‑direction table (Table 3, Ø MMAT per technology group), but no individual study is singled out as uniquely decisive for the conclusions. |
| **5** Investigation of heterogeneity in reported effects | State the method(s) used to examine heterogeneity in reported effects when it was not possible to undertake a meta-analysis of effect estimates and its extensions to investigate heterogeneity | **Pages 8–9, Methods → “Quality assessment and data synthesis”**  **Pages 12–17, Results → “Summary of main findings” → Section 1 “Adoption trends of digital health technologies”**  **Later Results pages 20–22, Results → TAM‑NPT effect‑direction analysis and Table 3** | Heterogeneity in reported effects is addressed both methodologically and in the substantive synthesis. Methodologically, pages 8–9 (Methods > Quality assessment and data synthesis) explain that the included studies report different but partly overlapping constructs across TAM and NPT domains, use mixed designs (qualitative, quantitative, mixed‑methods), and employ non‑comparable, study‑specific measurement scales, with only small numbers of directly comparable studies per technology–country combination. Under these conditions, the authors did not perform meta‑analysis but instead used narrative synthesis, vote counting, and effect‑direction mapping to preserve conceptual distinctions between technologies and constructs. Substantively, the Results (especially ‘Study characteristics’ and ‘Summary of main findings’, pages 10–24) examine heterogeneity by technology type (AI, DHTs, EHRs, telemedicine), geographical region (e.g. Northern/Western vs Eastern Europe), and temporal context (pre‑ vs post‑COVID‑19). The TAM–NPT effect‑direction analysis and Table 3 further explore heterogeneity in effect patterns across constructs and technology groups, while Multimedia Appendices 3, 5, and 7 provide stratified tables that allow readers to see how individual studies contribute to these heterogeneous patterns. |
| **6** Certainty of evidence | Describe the methods used to assess certainty of the synthesis findings | **Pages 8–9, Methods → “Quality assessment and data synthesis”**  **Results section pages 20–22, Results → TAM‑NPT effect‑direction analysis, Table 3**  **Pages 23–24, Discussion → “Strengths and limitations”** | Certainty of the synthesis findings is addressed using study‑level quality assessment and a transparent description of limitations. The methods section (pages 8–9, Methods > Quality assessment and data synthesis) reports that all 65 included studies were appraised with the Mixed Methods Appraisal Tool (MMAT) 2018, that MMAT ratings were used descriptively to characterise overall study quality and to avoid over‑interpreting isolated findings from lower‑scoring studies, and that 4 studies scored 60% while the remaining 61 scored ≥80%. The authors explicitly state that they did not apply a formal GRADE approach and that no formal outcome‑level certainty assessment was conducted because the evidence is highly heterogeneous and largely non‑comparative. In the Results, average MMAT scores by technology group are shown in the TAM–NPT effect‑direction table (Table 3, approximately pages 20–22), linking quality to synthesised patterns. The Discussion (pages 23–24, Strengths and limitations) reiterates that the predominance of qualitative and observational studies, potential self‑report and non‑response bias, and the absence of GRADE mean that the certainty of synthesised findings is limited and should be interpreted cautiously. |
| **7** Data presentation methods | Describe the graphical and tabular methods used to present the effects (e.g., tables, forest plots, harvest plots).  Specify key study characteristics (e.g., study design, risk of bias) used to order the studies, in the text and any tables or graphs, clearly referencing the studies included | **Page 8–9**, final paragraph, Methods → “Quality assessment and data synthesis”   - **Table 1:** Temporal distribution of studies – Results → “Study characteristics” → “Temporal distribution” (**page 11**) - **Table 2 & Figure 2:** Geographic distribution – Results → “Geographic distribution” (**page 12**) - **Effect‑direction table (Table 3):** TAM–NPT effect‑direction plot with percentages and Ø‑MMAT – later in Results, around **pages 20–22**. - **Multimedia Appendix 3:** Topic coding and extracted data. - **Multimedia Appendix 5:** Evidence table with characteristics and findings of individual studies.   Ordering and grouping of studies in presentations:   - Table 1 is ordered by **year of publication**. - Table 2 and Figure 2 order by **country**. - Table 3 orders by **technology group** and NPT/TAM constructs, with average MMAT per group. - MA 3 & MA 5 order studies by **technology group, design, and country**. | The methods describe how data are presented using descriptive tables and graphs. On pages 8–9 (Methods > Quality assessment and data synthesis), the authors state that descriptive statistics (frequencies and percentages) are used to characterise the included studies and to summarise quantitative findings, and that an effect‑direction table is used to visualise heterogeneous findings from TAM and NPT analyses. In the Results, these presentation methods are implemented as follows: Table 1 (page 11) presents the temporal distribution of studies by publication year; Table 2 and Figure 2 (page 12) present the geographic distribution by country; and the TAM–NPT effect‑direction table (Table 3, approximately pages 20–22) shows the proportion of studies in each effect‑direction category by technology group and construct, alongside average MMAT scores. Multimedia Appendices 3 and 5 provide detailed tabular presentations of study characteristics and findings (including technology group, country, study design, and MMAT rating). Across these tables and figures, studies and results are ordered and stratified by publication year, country, technology group (AI, DHTs, EHRs, telemedicine), and, where relevant, MMAT score or design, thereby making the structure of the synthesis transparent. |
| *Results* | | | |
| **8** Reporting results | For each comparison and outcome, provide a description of the synthesised findings, and the certainty of the findings. Describe the result in language that is consistent with the question the synthesis addresses, and indicate which studies contribute to the synthesis | **Pages 12–24, Results → “Summary of main findings”**  **Pages 20–22, Results → TAM–NPT effect‑direction analysis + Table 3** | Synthesised findings are reported in the Results section, primarily under ‘Summary of main findings’ (pages 12–24) and in the TAM–NPT effect‑direction analysis. For each outcome domain—(1) adoption trends of digital health technologies, (2) GP experiences (benefits, workflow changes, patient interactions), (3) impacts on workload, efficiency, and ethical concerns, (4) barriers to implementation, and (5) policy and practice implications—the authors provide narrative summaries of the synthesised results, with explicit reference to the contributing studies via in‑text citations. These summaries are structured by technology group and, where relevant, by country and time period. In addition, the TAM–NPT effect‑direction analysis and Table 3 (approximately pages 20–22) report synthesised percentages for each construct (e.g. perceived usefulness, ease of use, coherence, cognitive participation, collective action, reflexive monitoring) within each technology group, explicitly quantifying patterns such as ‘Perceived usefulness was high (86.2% positive)’ and ‘NPT constructs showed partial normalization…’. Multimedia Appendices 3, 5, and 7 provide the underlying study‑level data and mappings that show which studies contribute to each synthesised pattern. |
| *Discussion* |  |  |  |
| **9** Limitations of the synthesis | Report the limitations of the synthesis methods used and/or the groupings used in the synthesis, and how these affect the conclusions that can be drawn in relation to the original review question | **Pages 23-24, Strengths and limitations, paragraph 2** | Limitations of the synthesis methods and groupings, and their implications for the conclusions, are discussed in the Discussion section under ‘Strengths and limitations’ (pages 23–24). The authors note that the evidence base is highly heterogeneous and predominantly qualitative or observational, which limits causal inference and constrains the types of synthesis methods that are appropriate. In this context, they explain that they did not conduct a formal meta‑analysis but relied on a structured narrative synthesis with vote counting and effect‑direction mapping, and that this approach, while preserving conceptual distinctions between technologies and constructs, remains sensitive to how effect‑direction categories and thresholds are defined and cannot fully overcome publication bias or selective outcome reporting. They further highlight that most studies come from Western and Northern Europe, with sparse evidence from Eastern and Southern Europe, which may limit the generalisability of synthesised patterns, and that, although MMAT scores were generally high, the absence of a formal GRADE assessment means that the certainty of synthesised findings is limited and should be interpreted cautiously. |

**PRISMA=Preferred Reporting Items for Systematic Reviews and Meta-Analyses.**

*If the information is not provided in the systematic review, give details of where this information is available (e.g., protocol, other published papers (provide citation details), or website (provide the URL)).

**Abbreviations**

AI: Artificial intelligence

DHT: Digital health tool

EHR: Electronic health record

EMR: Electronic medical record

GP: General practitioner

GRADE: Grading of Recommendations Assessment, Development, and Evaluation

MMAT: Mixed Methods Appraisal Tool

NPT: Normalization Process Theory

OSF: Open Science Framework

PRISMA: Preferred Reporting Items for Systematic Reviews and Meta-Analyses

SWiM: Synthesis Without Meta-analysis

TAM: Technology Acceptance Model

**References**

24. Popay J, Roberts H, Sowden A, et al. Guidance on the Conduct of Narrative Synthesis in Systematic Reviews: A Product from the ESRC Methods Programme. Lancaster University; 2006. [doi: 10.13140/2.1.1018.4643]
