# Supplementary material for: The Impact of Digitalization on European General Practice From the Perspective of General Practitioners: Systematic Review
Source: J Med Internet Res. 2026 Aug 18;28:e83343. doi: 10.2196/83343 (PMC13485116; doi:10.2196/83343)
Supplement: Supplementary file 9 [file jmir-v28-e83343-s010.docx]

**PRESS 2015 Evidence-Based Checklist**

| **PRESS Element** | **Our Search Strategy Implementation** | **Assessment** | **Comments/Justification** |
| --- | --- | --- | --- |
| **1. TRANSLATION OF RESEARCH QUESTION** |  |  |  |
| **Match to PICO/Research Question** |  |  | Search terms appropriately reflect all PICO elements. |
| Population | GPs/family physicians in EU member states, UK, Norway, Switzerland. Search included country names: "Albania" through "Vatican City" (all European countries) | ✓ Comprehensive | Explicitly included all relevant European countries. |
| Intervention | Digital health concepts: "digital*", "digitalization", "eHealth", "telemedicine", "artificial intelligence" | ✓ Well-translated | Broad terms to capture emerging technologies. |
| Context | "primary care", "general practice", "general practitioner", "family doctor" | ✓ Appropriate | Multiple synonyms for the primary care setting. |
| Outcomes | Captured through broad search without outcome restrictions | ✓ Inclusive approach | Appropriate for scoping all impacts/attitudes. |
| **Search concept clarity** | Core Boolean: (digital OR digitalization OR eHealth OR telemedicine OR AI) AND (primary care OR general practice OR GP) | ✓ Clear structure | Consistent across databases, Broad enough to capture relevant literature. |
| **2. BOOLEAN AND PROXIMITY OPERATORS** |  |  |  |
| **Correct use of AND/OR** | - OR used within concept groups<br>- AND used between concepts<br>- Example from PubMed: ((general practice [Title]) OR (primary care[Title])) AND (digital*[Title]) | ✓ Correctly applied | Logical operators are used throughout. Database-specific syntax adaptations were necessary. |
| **Nesting with brackets** | Properly nested throughout, e.g., Scopus: TITLE-ABS ("digital health" OR ehealth OR "digital technology") | ✓ Appropriate nesting | Maintained search logic integrity, simplified for some databases with limited functionality. |
| **Proximity operators** | Not used - relied on phrase searching ("digital health", "general practice") | ⚠️ Not utilized | Phrase searching was used instead to keep the strategy manageable across databases; it may have missed variants. |
| **3. SUBJECT HEADINGS** |  |  |  |
| **Database-specific headings** | Adapted to each database's available indexing | ✓ Database-appropriate | Heterogeneous indexing required database-specific strategies, suitable subject headings for digital health were scarce/heterogeneous, so a primarily text-word–based approach was chosen. |
| PubMed | Used field tags [Title] and [pdat] for publication date | ⚠️ Limited MeSH use | Relied primarily on title/keyword searching. |
| Scopus | TITLE-ABS-KEY fields utilized | ✓ Appropriate for database | Used Scopus-specific field codes. |
| Cochrane | Used: ti,ab,kw field indicators | ✓ Database-appropriate | Cochrane-specific syntax applied. |
| **Explosion of terms** | Not explicitly documented | ⚠️ Not reported | May have missed narrower terms. |
| **Relevant MeSH terms** | Used where available (e.g., PubMed: general practice[Title]) | ⚠️ Limited use | Many databases lacked appropriate subject headings for digital health concepts. |
| **4. TEXT WORD SEARCHING** |  |  | Term selection validated against known papers |
| **Spelling variants** | Both "digitalization" and "digitalisation" could be considered | ⚠️ Partially addressed | UK/US spelling not explicitly shown for all terms, truncation and broad terms were expected to capture the main spelling variants, and any residual loss was considered acceptable. |
| **Synonyms/Related terms** | Comprehensive synonyms:<br>- Digital concepts: digital, digitalization, eHealth, telemedicine, artificial intelligence<br>- Primary care: primary care, general practice, general practitioner, family doctor/physician | ✓ Extensive synonyms | Good coverage of concept variations. |
| **Truncation** | Used appropriately: "digital*" to capture digital, digitalization, digitally, etc. | ✓ Correctly applied | Efficient use of truncation. |
| **Acronyms** | "AI" not used in search (used full "artificial intelligence") | ✓ Good practice | Avoided ambiguous abbreviations. |
| **Field specification** | - PubMed: [Title] fields<br>- Scopus: TITLE-ABS-KEY<br>- WoS: TS (Topic) | ✓ Appropriate fields | Database-specific field selection. |
| **5. SPELLING, SYNTAX, AND LINE NUMBERS** |  |  |  |
| **Spelling errors** | No spelling errors identified in documented searches | ✓ Error-free |  |
| **Database syntax** | Each database shows adapted syntax:<br>- PubMed: [Title], [pdat]<br>- Scopus: TITLE-ABS, PUBYEAR<br>- Cochrane: :ti,ab,kw<br>- Web of Science: TS= | ✓ Correctly adapted | Proper syntax for each platform. Each database required syntax adaptation. |
| **Line combinations** | Not shown in line-by-line format but Boolean structure clear | N/A, ✓ Properly documented | Searches presented as complete strings in MA 1. |
| **6. LIMITS AND FILTERS** |  |  |  |
| **Date limits** | 2014-2024 (past 10 years) | ✓ Justified | Captures recent digitalization trends |
| **Language limits** | English and German | ⚠️ Potential limitation | Acknowledged as a limitation, it may have missed other European languages. |
| **Geographic limits** | Comprehensive list of European countries (EU, UK, Norway, Switzerland) | ✓ Appropriate | Matches study scope. |
| **Publication type** | Peer-reviewed articles; no study design restrictions | ✓ Inclusive | Appropriate for comprehensive review. |
| **Quantitative filters** | Scopus specifically included: "quantitative OR outcomes OR effectiveness OR impact OR evaluation" | ✓ Relevant additions | Helped focus on empirical studies. |

| **Quality Element** | **Implementation** | **Evidence** |
| --- | --- | --- |
| **Known papers validation** | Search strategy validated against a predefined list of known relevant studies. | Mentioned in MA 1 as "validation markers". |
| **Database coverage** | 8 databases searched: PubMed, Wiley, Scopus, Cochrane, Web of Science, Google Scholar, Springer Link, Ovid. | Comprehensive coverage, including gray literature. |
| **Gray literature** | Elicit AI is explicitly used to identify gray literature. | Innovative approach to find unpublished work. |
| **Search documentation** | Full search strings are provided for each database. | Complete transparency in MA 1. |
| **Reproducibility** | Database-specific searches are documented with exact syntax. | Allows replication despite platform differences. |

| **Database** | **Search Adaptation** | **Rationale** |
| --- | --- | --- |
| PubMed | Used Title field restrictions and date limits. | Most comprehensive indexing for medical literature. |
| Wiley | Keyword search with publication title field. | Limited advanced search functionality. |
| Scopus | TITLE-ABS-KEY fields with geographic filters. | Broader interdisciplinary coverage. |
| Cochrane | Combined terms with Cochrane-specific syntax. | Specialized for systematic reviews. |
| Web of Science | Topic search with citation indexing. | Cross-disciplinary coverage. |
| Google Scholar | Simplified Boolean search. | Limited advanced search options. |
| Springer Link | Keyword search using complete Boolean logic. | Interdisciplinary coverage and supports field-restricted queries, interface constraints. |
| Ovid | Medical subject headings with explosion, no proximity operators. | Comprehensive medical database. |
| Elicit AI | Natural language queries with validation markers. | AI-assisted identification of gray literature. |

# References

1.	Kairouz P, McMahan HB. Getting started with elicit: which workflow or tool should I use? Accessed July 23, 2025. https://support.elicit.com/en/articles/1418881

**Abbreviations**

AI: Artificial intelligence

EU: European Union

GP: General practitioner

MeSH: Medical Subject Headings

PRESS: Peer Review of Electronic Search Strategies

UK: United Kingdom
